# Supplementary material for: Stereoselective Synthesis of Multisubstituted Cyclohexanes by Reaction of Conjugated Enynones with Malononitrile in the Presence of LDA
Source: Molecules. 2020 Dec 14;25(24):5920. doi: 10.3390/molecules25245920 (PMC7765106; doi:10.3390/molecules25245920)

Stereoselective synthesis of multisubstituted cyclohexanes by reaction of conjugated enynones with malononitrile and LDA

Anastasiya V. Igushkina,[a] Alexander A. Golovanov,[b] Irina A. Boyarskaya,[a] Ilya E. Kolesnikov,[c] Aleksander V. Vasilyev*[a,d]

[a]*Department of Organic Chemistry, Institute of Chemistry, Saint Petersburg State University, Universitetskaya nab., 7/9, Saint Petersburg, 199034, Russia.*

[b]*Department of Chemistry, Chemical Processes and Technologies, Togliatti State University, Belorusskaya ul., 14, Togliatti, 445667, Russia.*

[c]*Center for Optical and Laser Materials Research, St. Petersburg State University, Ulyanovskaya ul., 5, Saint Petersburg, Petrodvoretz, 198504, Russia*

[d]*Department of Chemistry, Saint Petersburg State Forest Technical University, Institutsky per., 5, Saint Petersburg, 194021, Russia*

*Corresponding author: A.V. Vasilyev; e-mails: *aleksvasil@mail.ru; a.vasilyev@spbu.ru*

Contents

1. ^{1}H, NOESY H-H, ^{13}C, NMR and IR spectra of compounds **2**, **3** and **4**..……………………..S2

2. X-Ray data for compounds **2b**, **2e**, **3a**……….…………………………………………....S31

3. HPLC data for mixture of **3a** and **4a,b**……………………….…….…………………..……S58

4. Photoluminescent spectra of cyclohexanes **2a**, **2b**, **2c**, **2e**…………………………………..…..S59

5. Data of DFT calculations of stereoisomers of **2a**……………………………………………S64

1. ^{1}H, NOESY H-H, ^{13}C, NMR and IR spectra of compounds **2**, **3** and **4**

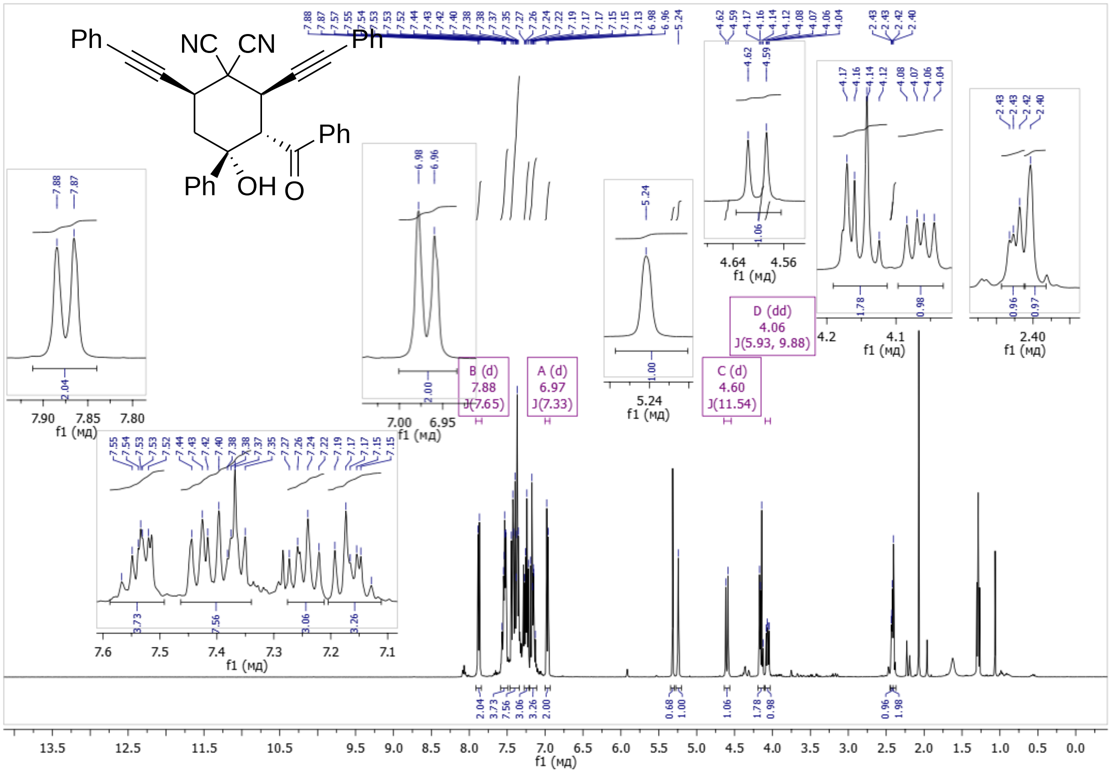

Figure S1. ^{1}H NMR spectrum of the compound **2a** ($CDCl_3$, 400 MHz).

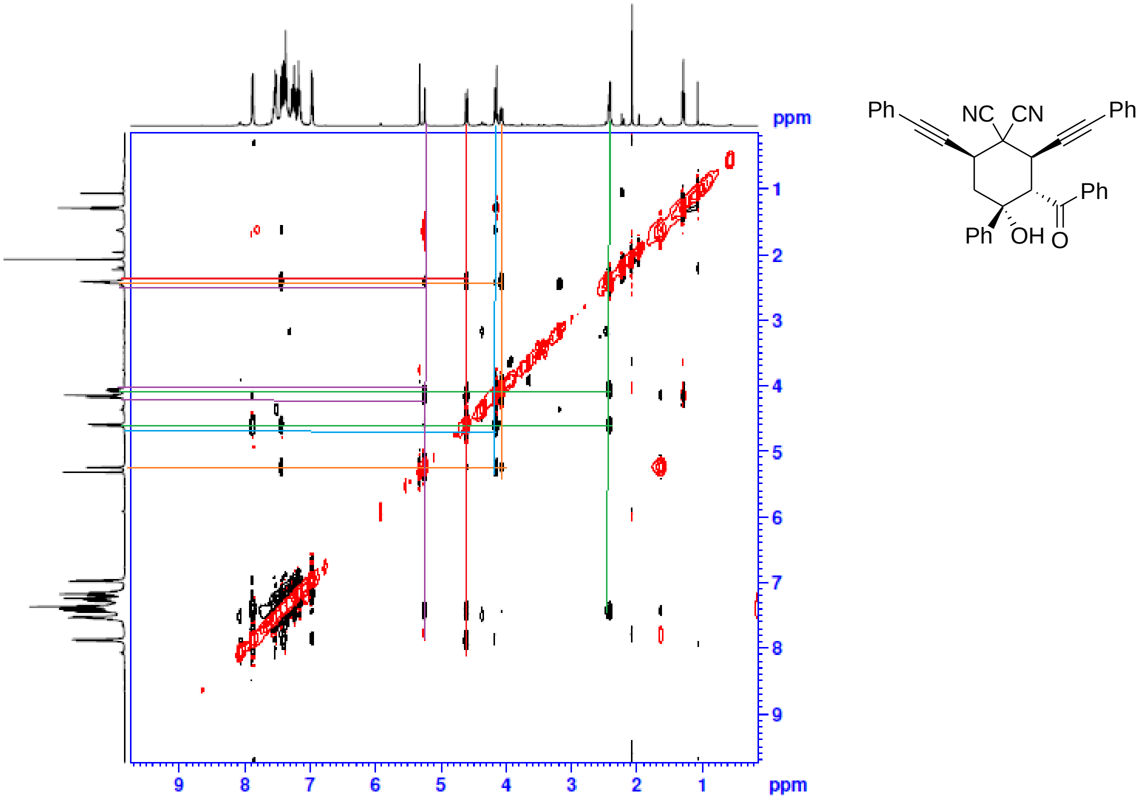

Figure S2. NOESY H-H NMR spectrum of the compound **2a** ($CDCl_3$).

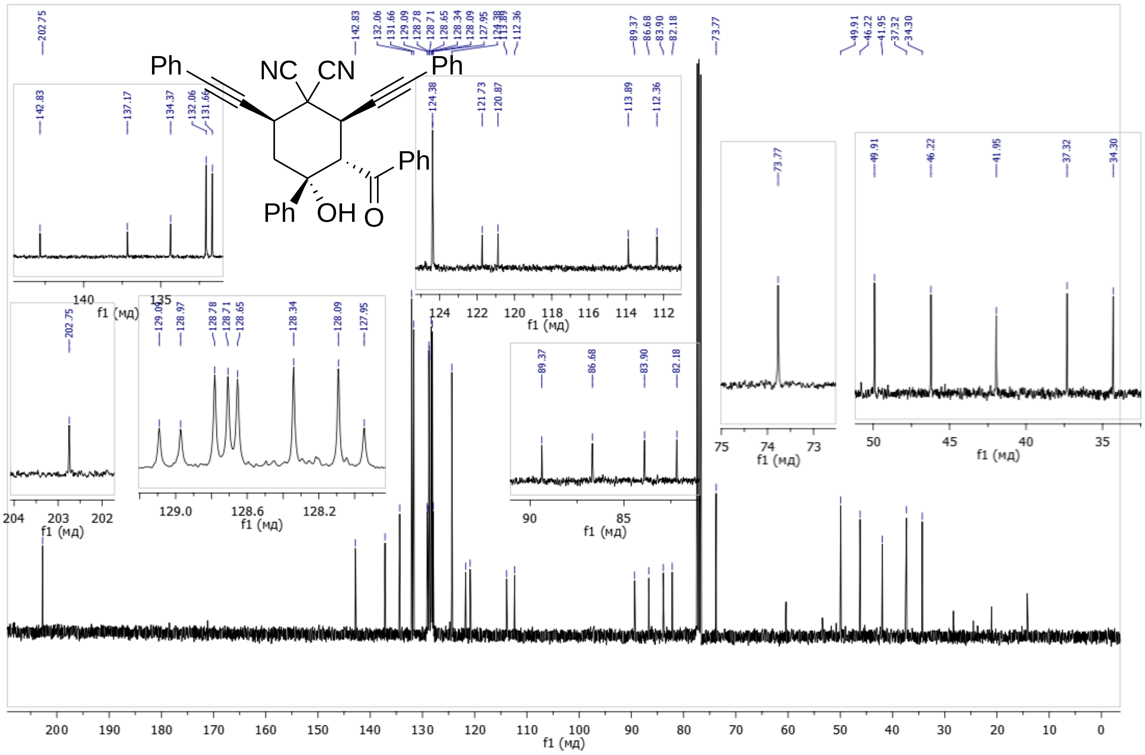

Figure S3. ^{13}C NMR spectrum of the compound **2a** ($CDCl_3$, 100 MHz).

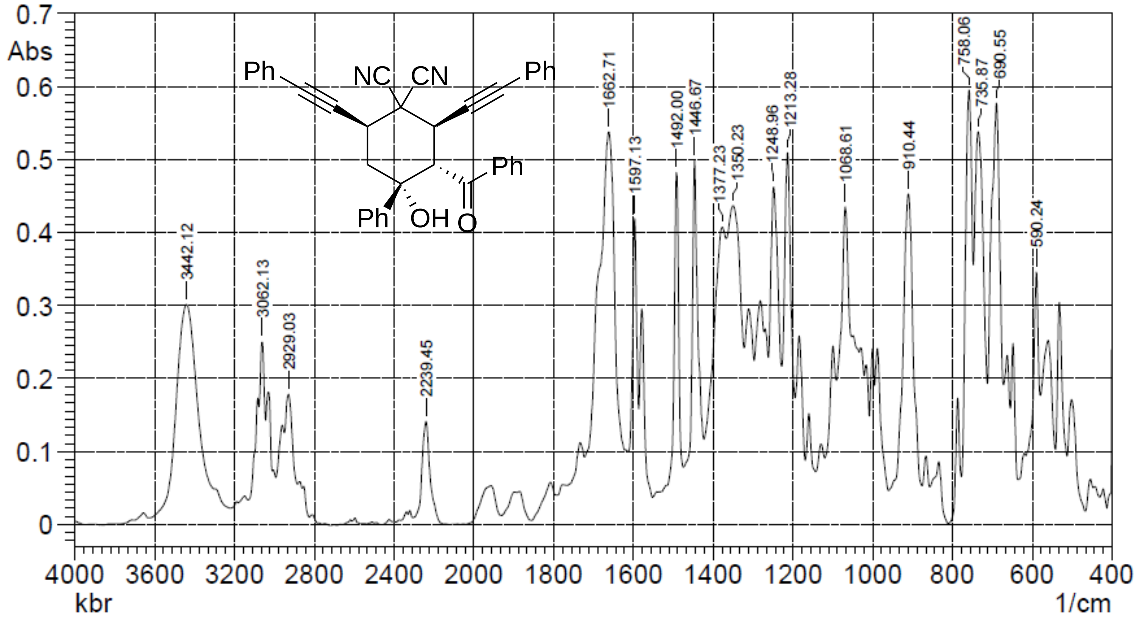

Figure S4. IR of the compound **2a** (KBr).

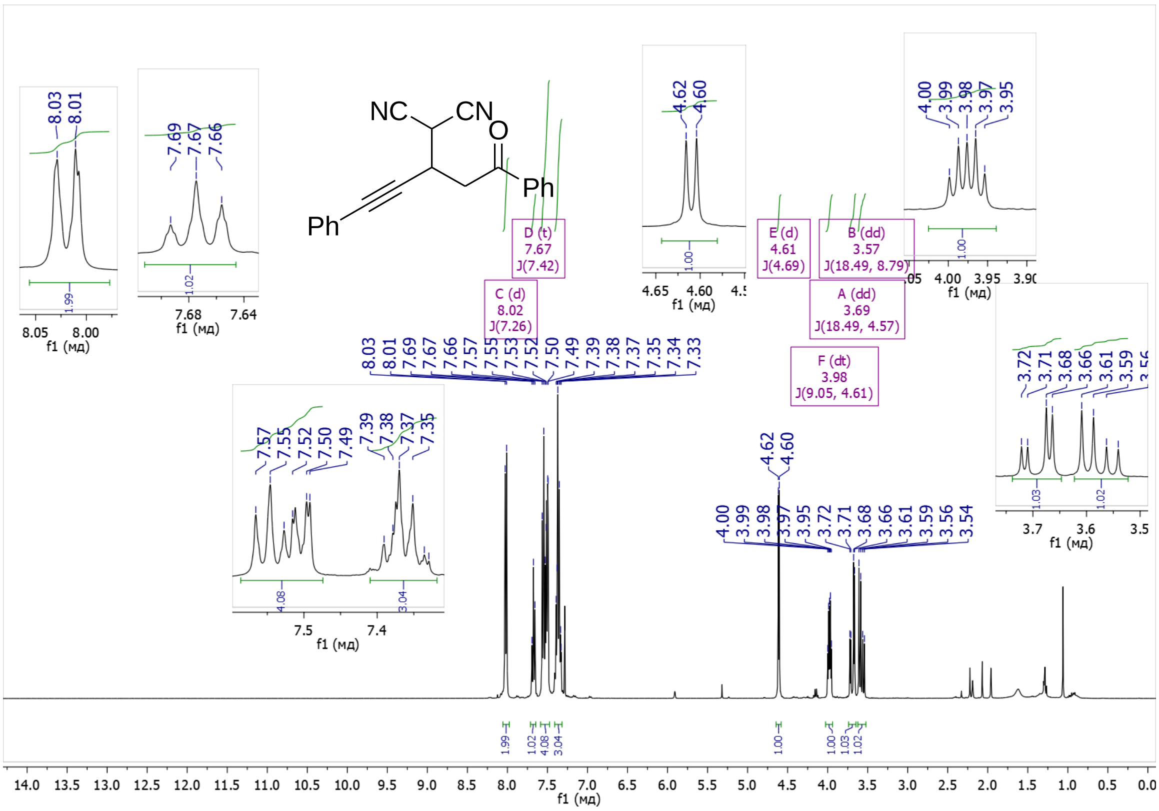

Figure S5. ^{1}H NMR spectrum of the compound **3a** ($CDCl_3$, 400 MHz).

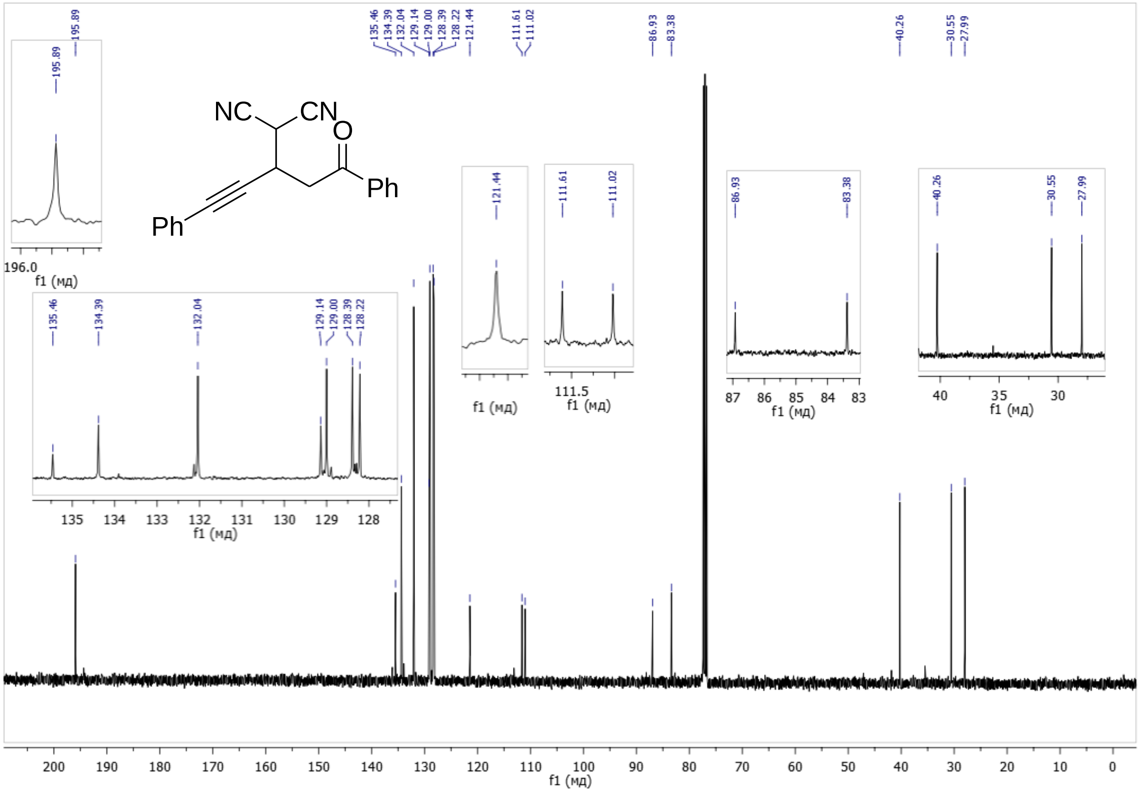

Figure S6. ^{13}C NMR spectrum of the compound **3a** ($CDCl_3$, 100 MHz).

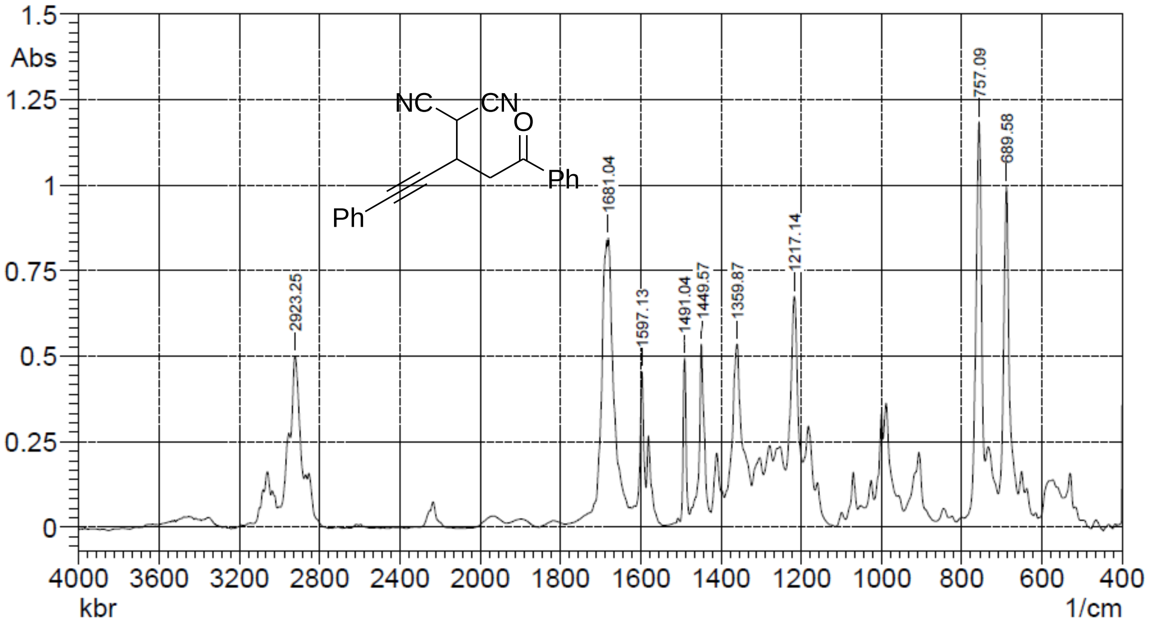

Figure S7. IR of the compound **3a** (KBr).

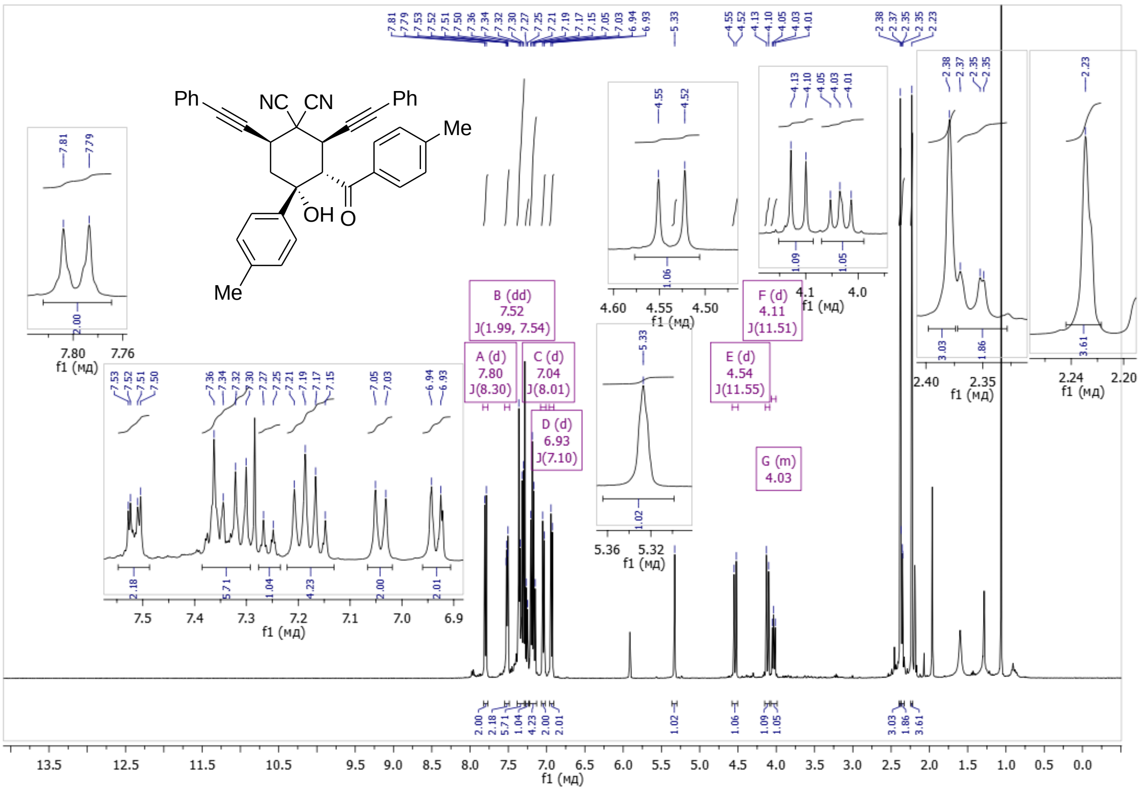

Figure S8. ^{1}H NMR spectrum of the compound **2b** ($CDCl_3$, 400 MHz).

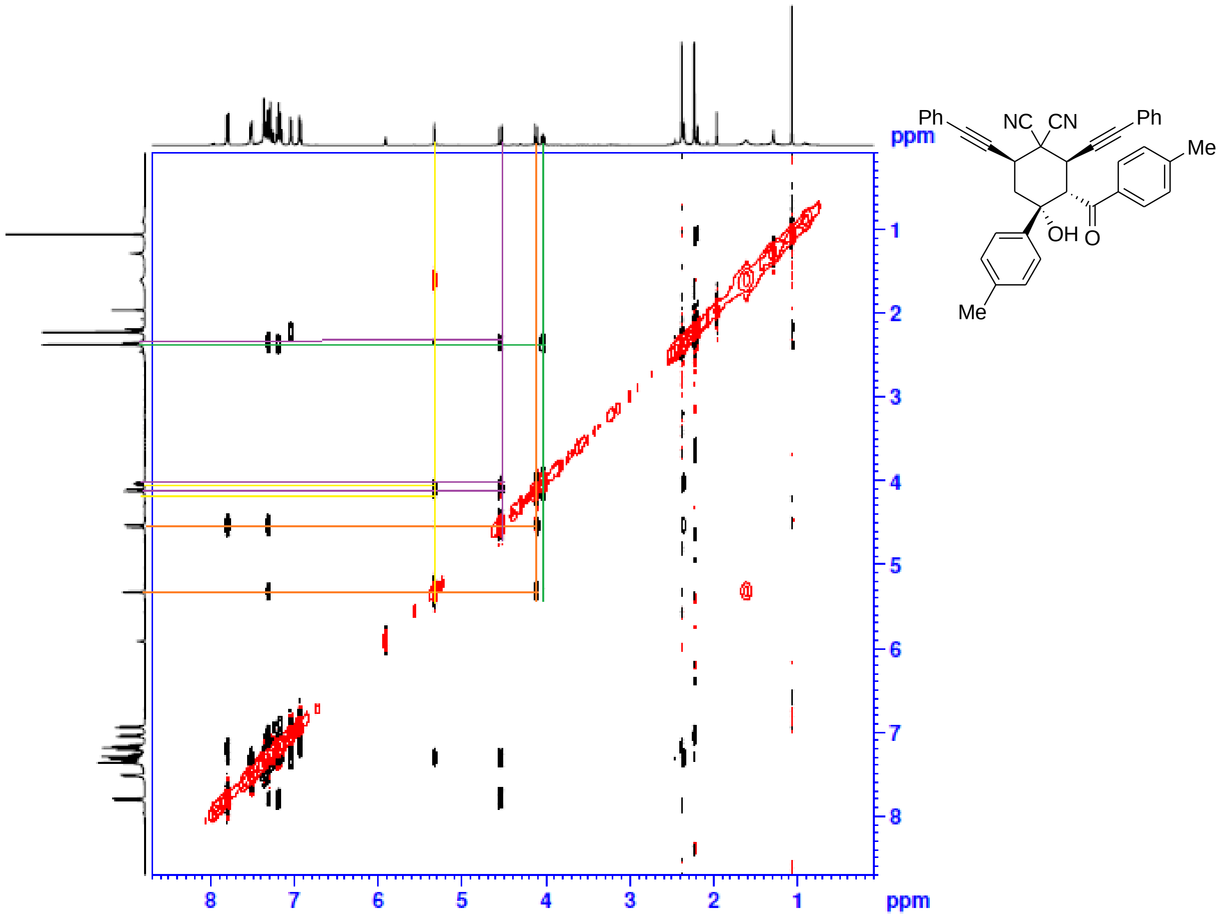

Figure S9. NOESY H-H NMR spectrum of the compound **2b** ($CDCl_3$).

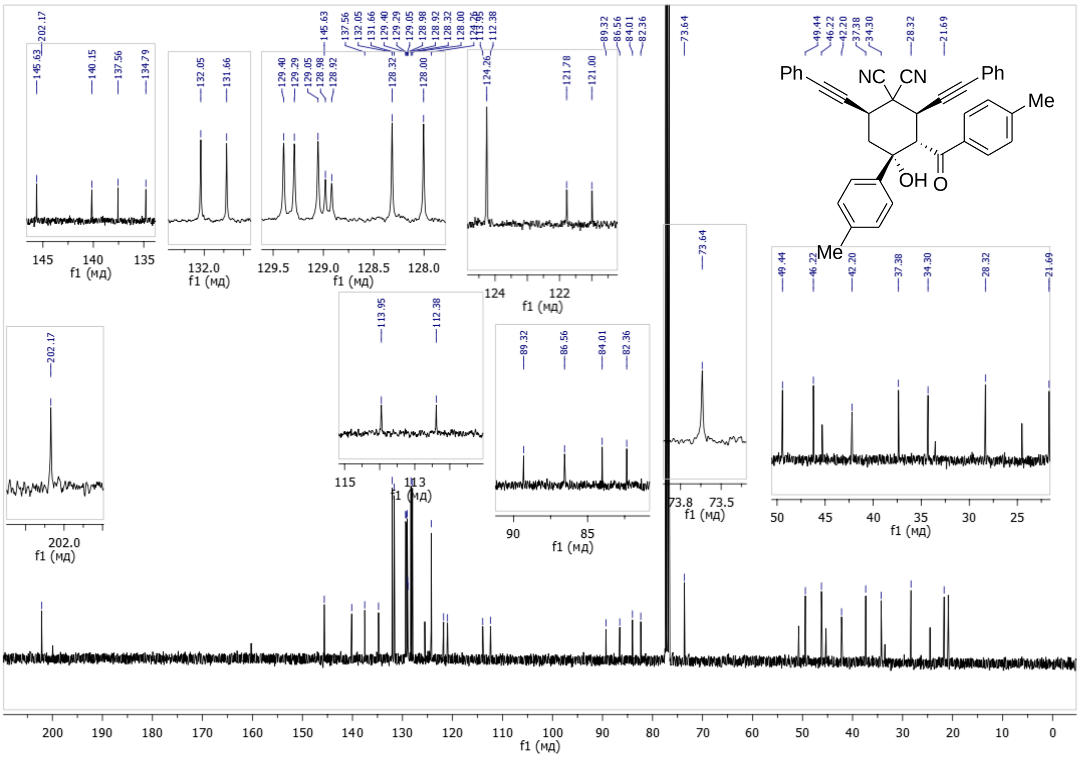

Figure S10. ^{13}C NMR spectrum of the compound **2b** ($CDCl_3$, 100 MHz).

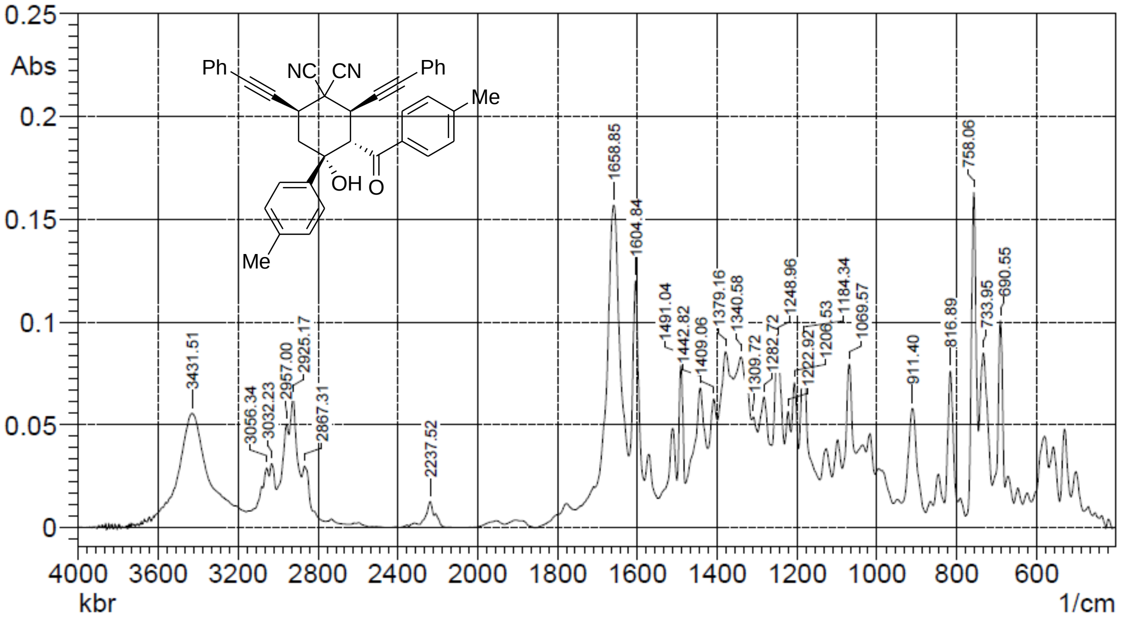

Figure S11. IR of the compound **2b** (KBr).

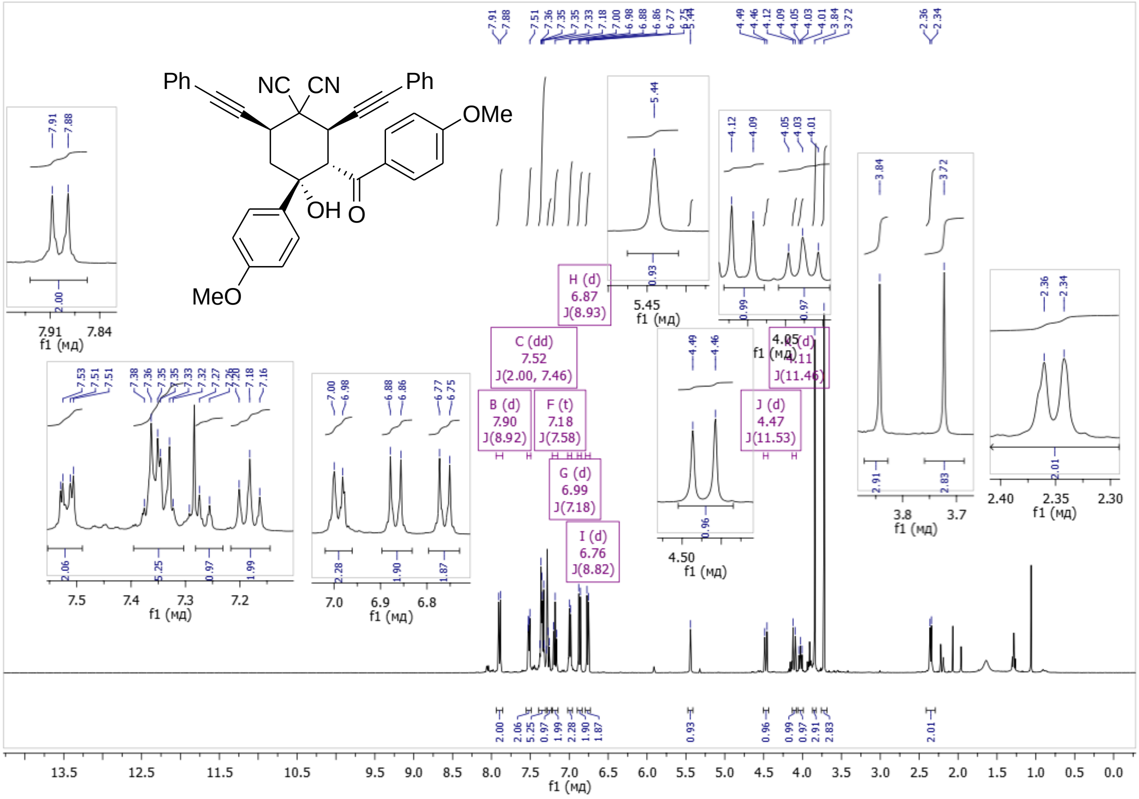

Figure S12. ^{1}H NMR spectrum of the compound **2c** ($CDCl_3$, 400 MHz).

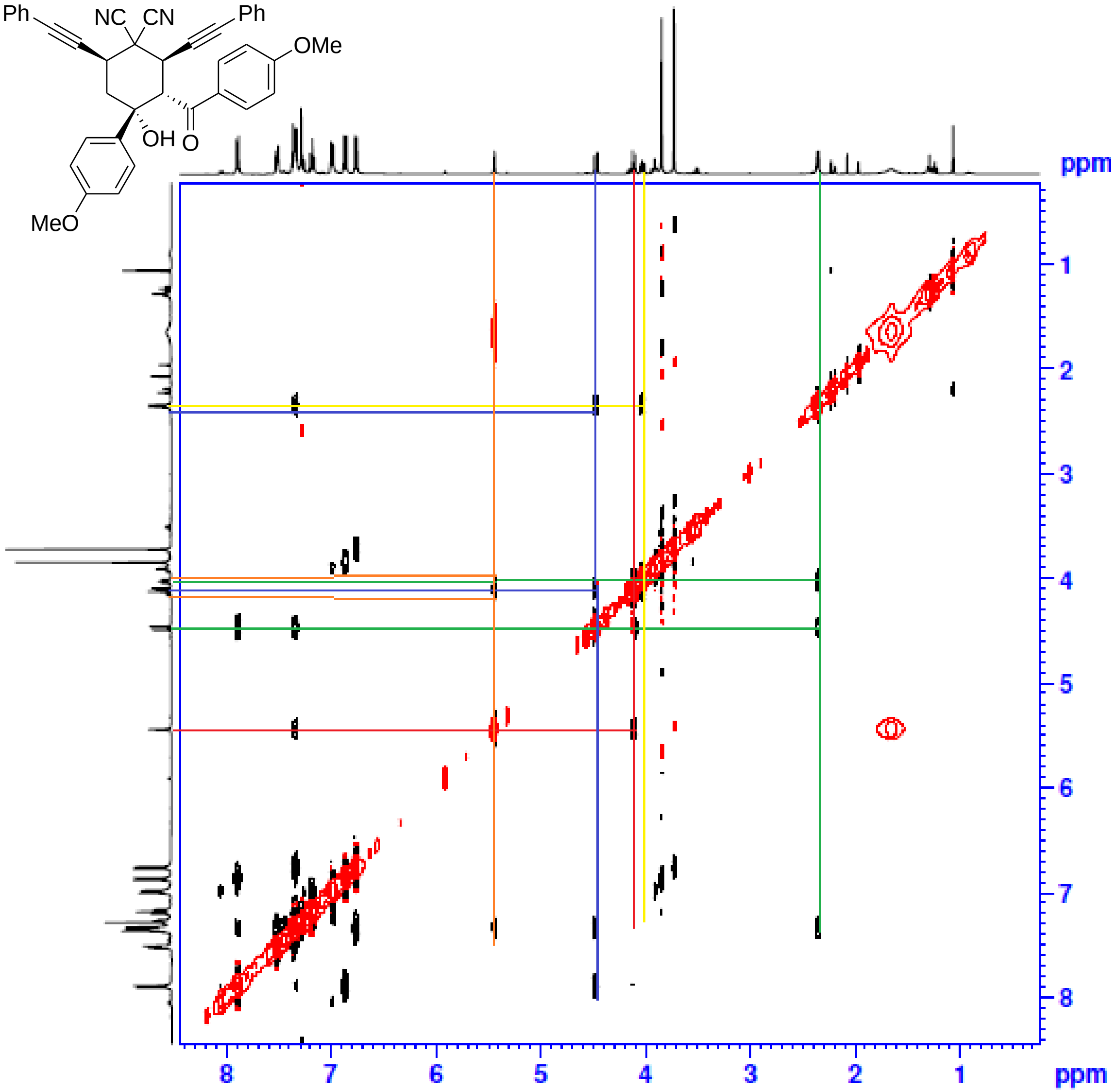

Figure S13. NOESY H-H NMR spectrum of the compound **2c** ($CDCl_3$).

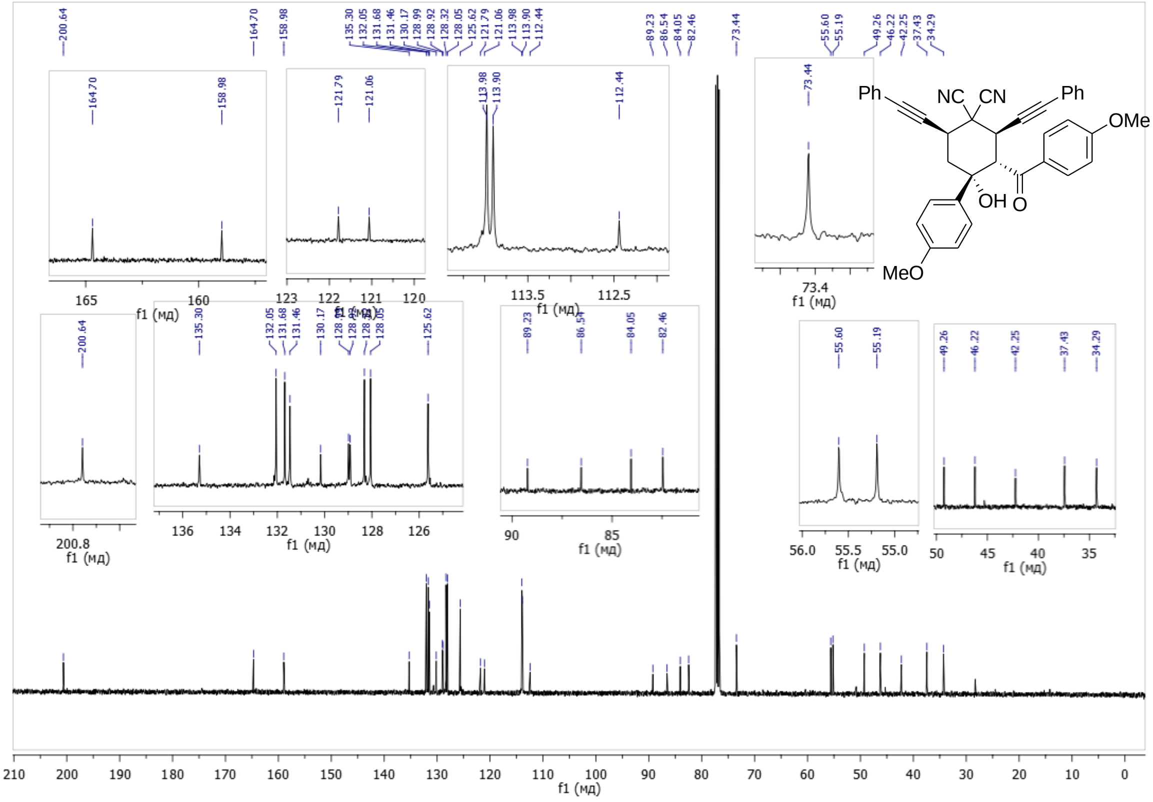

Figure S14. ^{13}C NMR spectrum of the compound **2c** ($CDCl_3$, 100 MHz).

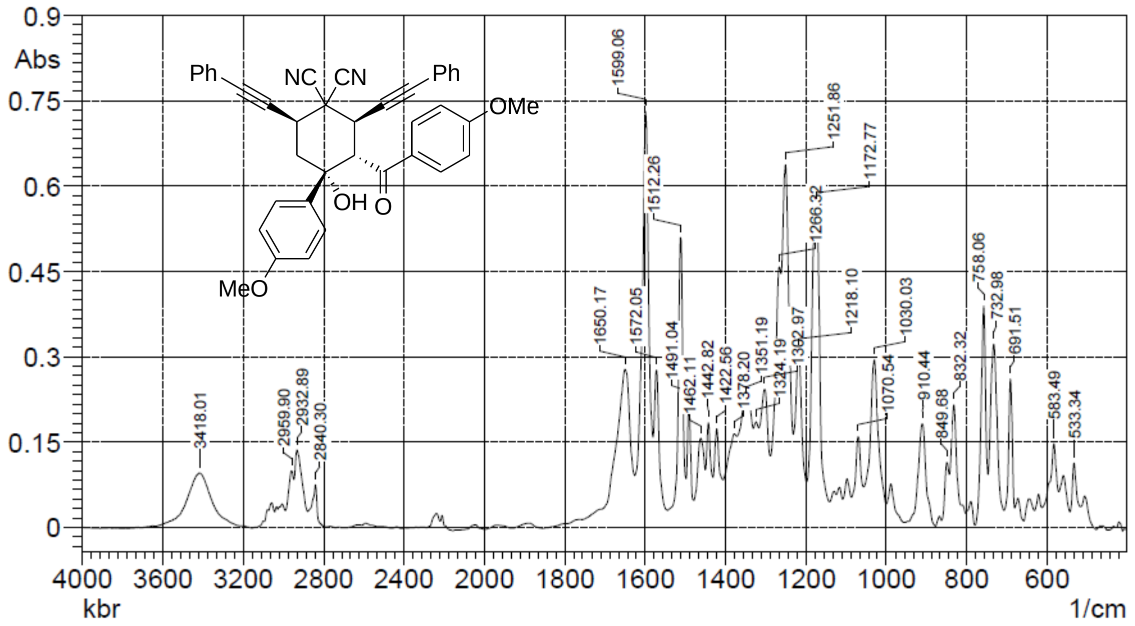

Figure S15. IR of the compound **2c** (KBr).

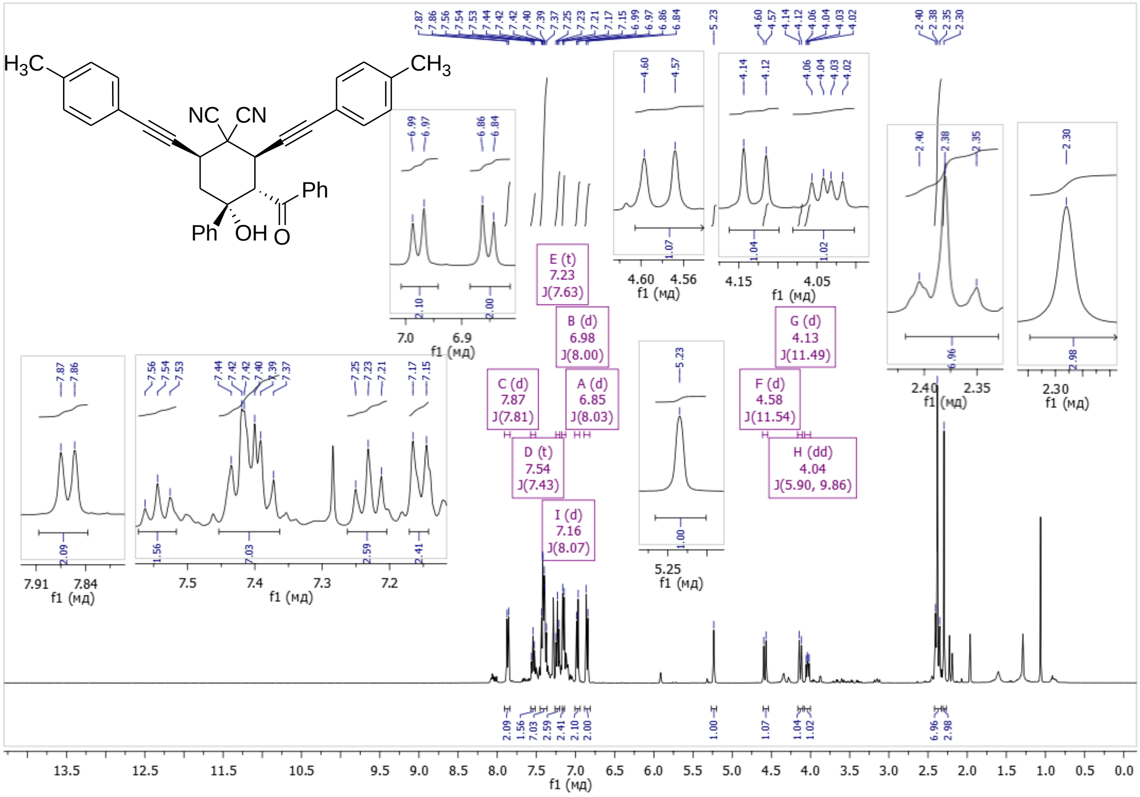

Figure S16. ^{1}H NMR spectrum of the compound **2d** ($CDCl_3$, 400 MHz).

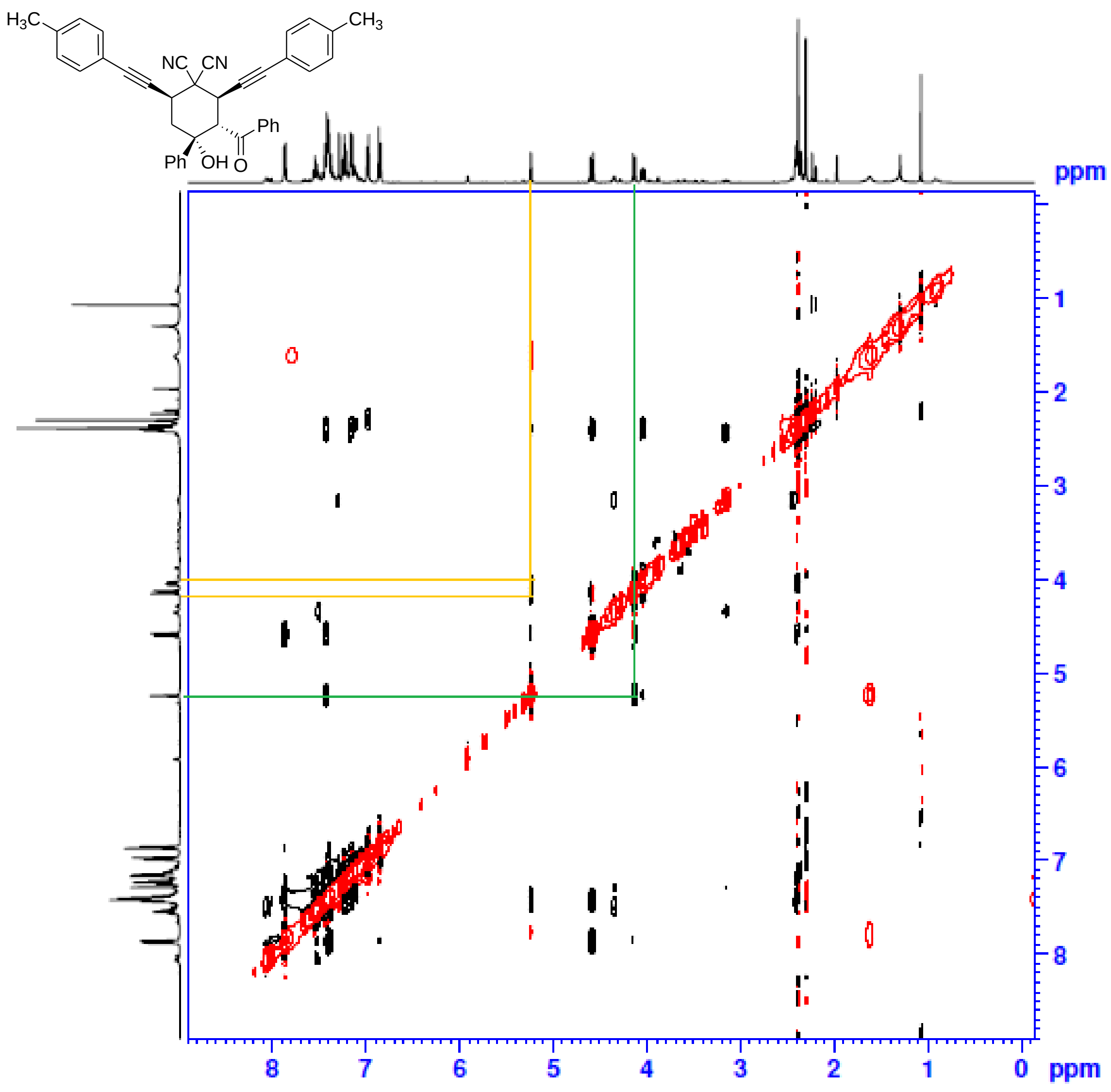

Figure S17. NOESY H-H NMR spectrum of the compound **2d** ($CDCl_3$).

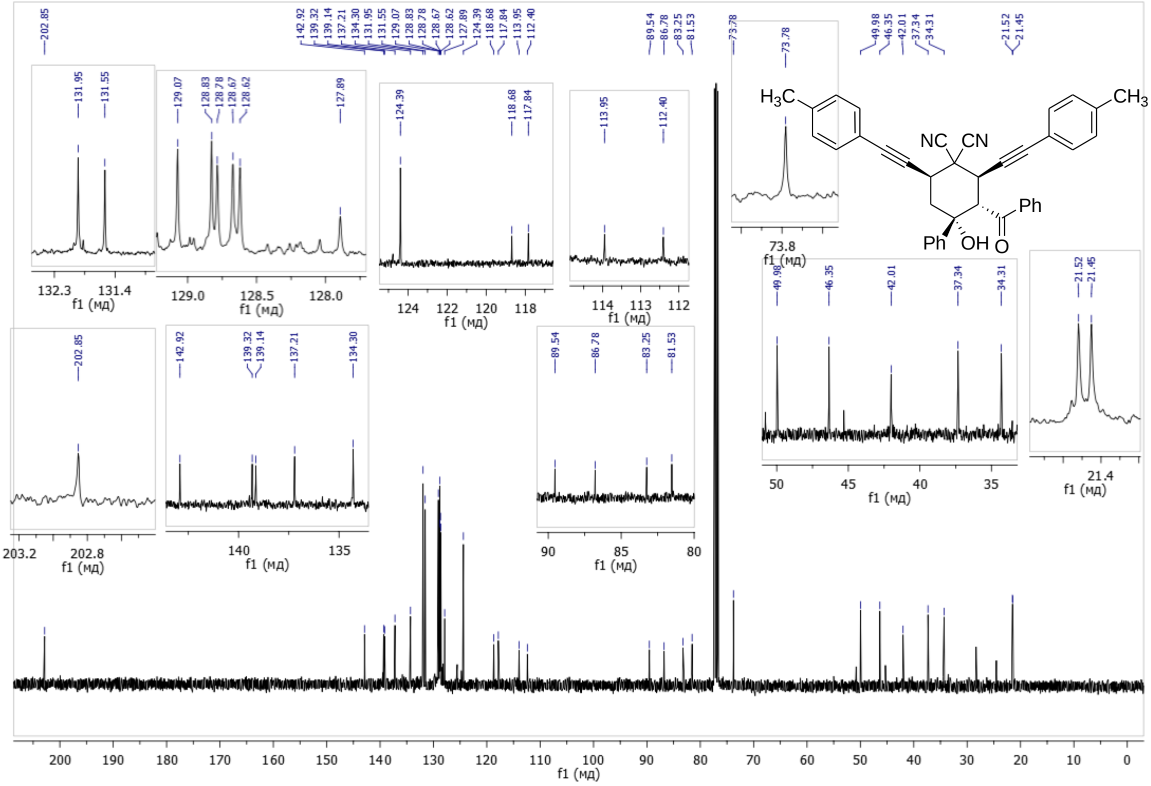

Figure S18. ^{13}C NMR spectrum of the compound **2d** ($CDCl_3$, 100 MHz).

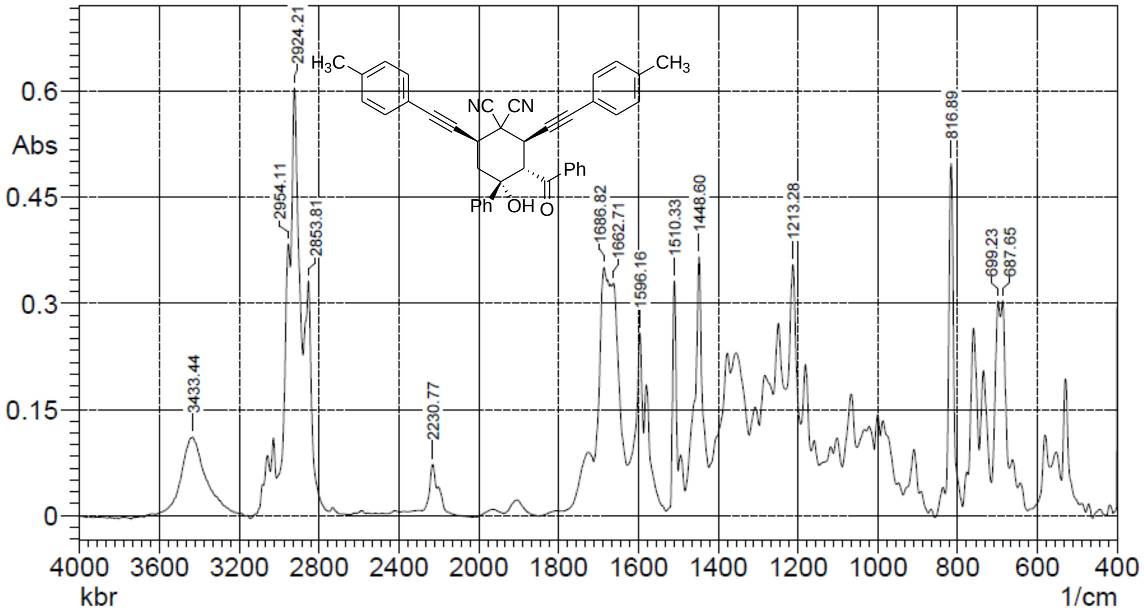

Figure S19. IR of the compound **2d** (KBr).

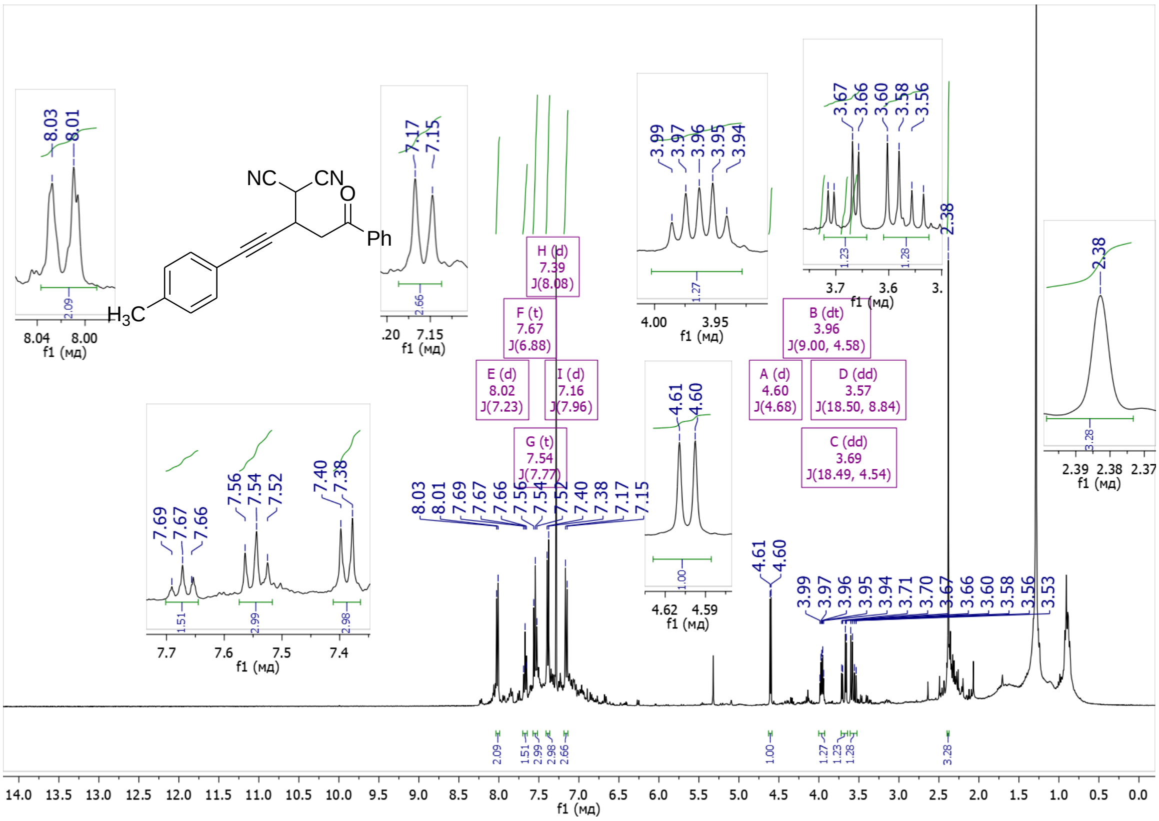

Figure S20. ^{1}H NMR spectrum of the compound **3d** ($CDCl_3$, 400 MHz).

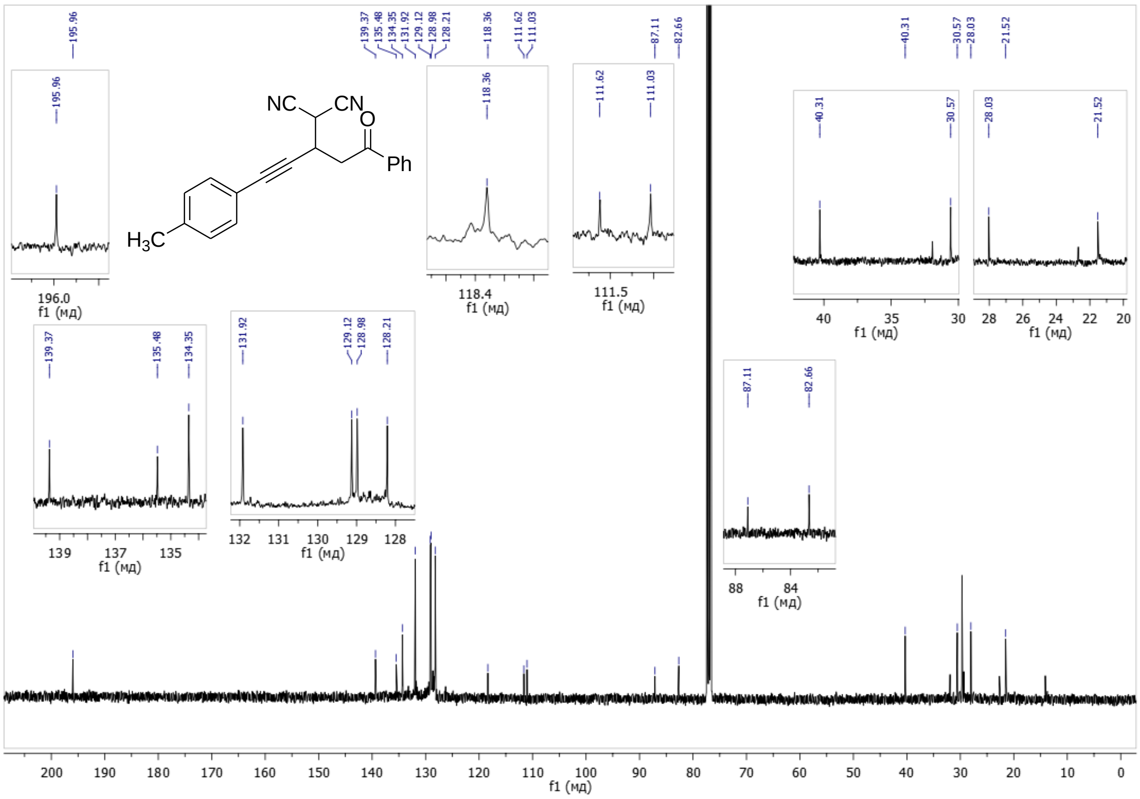

Figure S21. ^{13}C NMR spectrum of the compound **3d** ($CDCl_3$, 100 MHz).

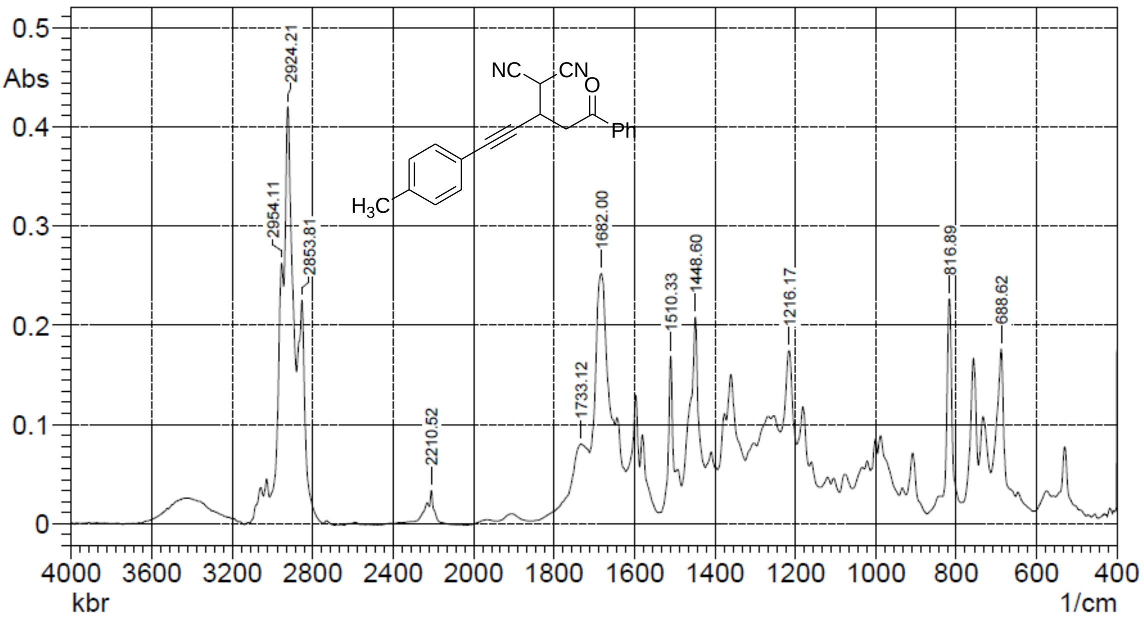

Figure S22. IR of the compound **3d** (KBr).

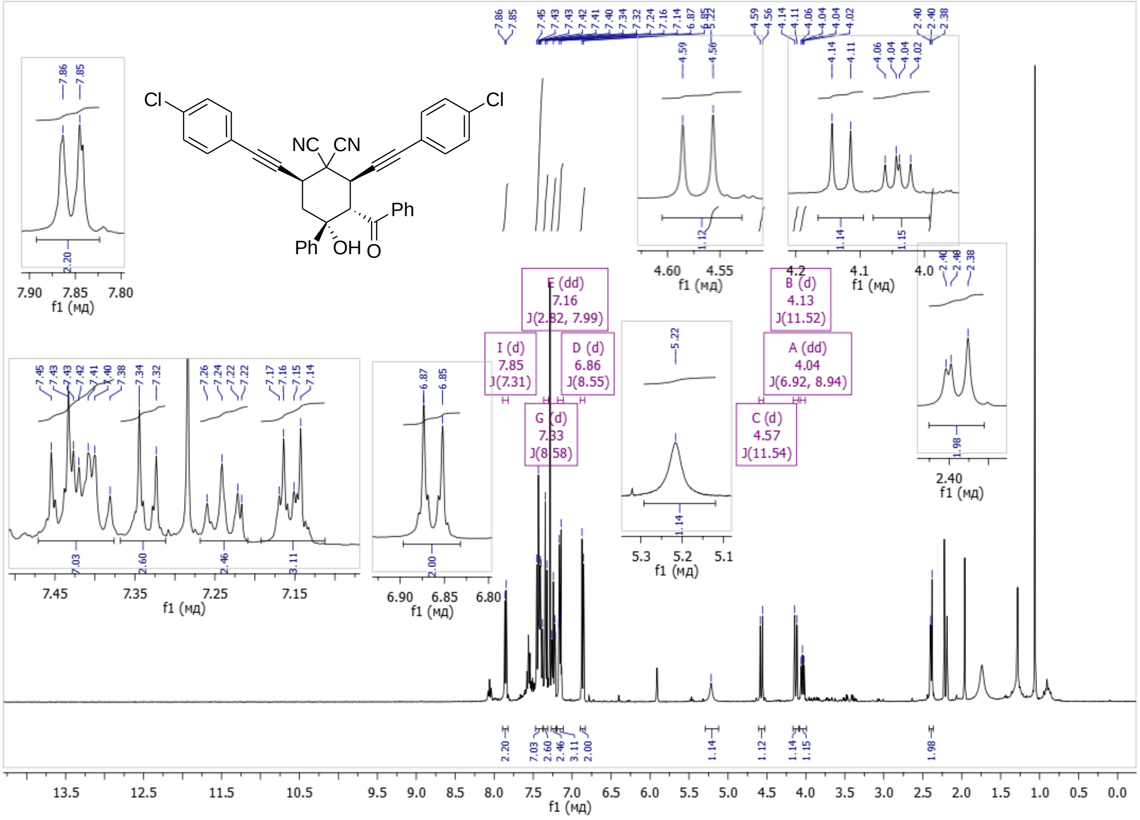

Figure S23. ^{1}H NMR spectrum of the compound **2e** ($CDCl_3$, 400 MHz).

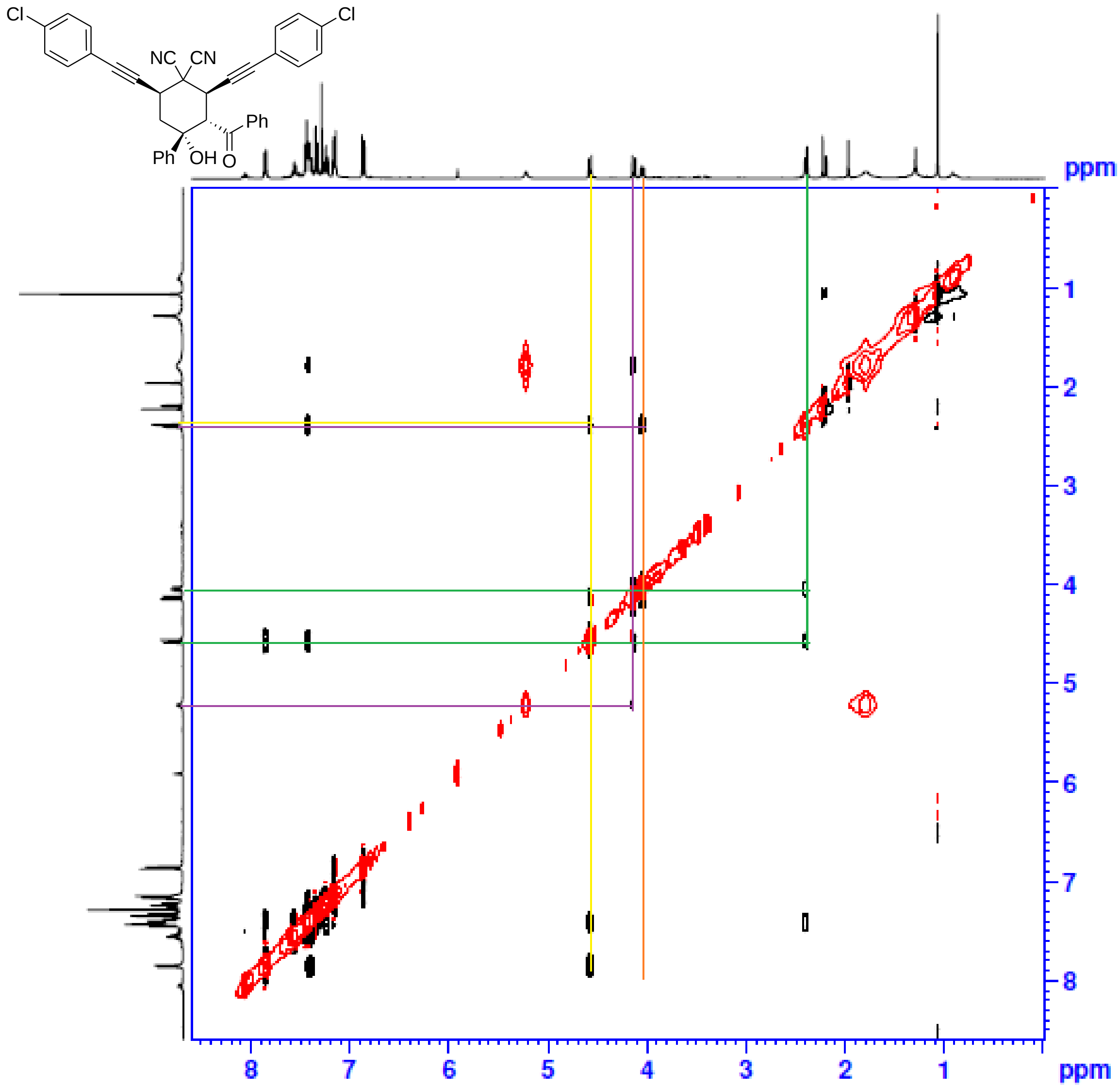

Figure S24. NOESY H-H NMR spectrum of the compound **2e** ($CDCl_3$).

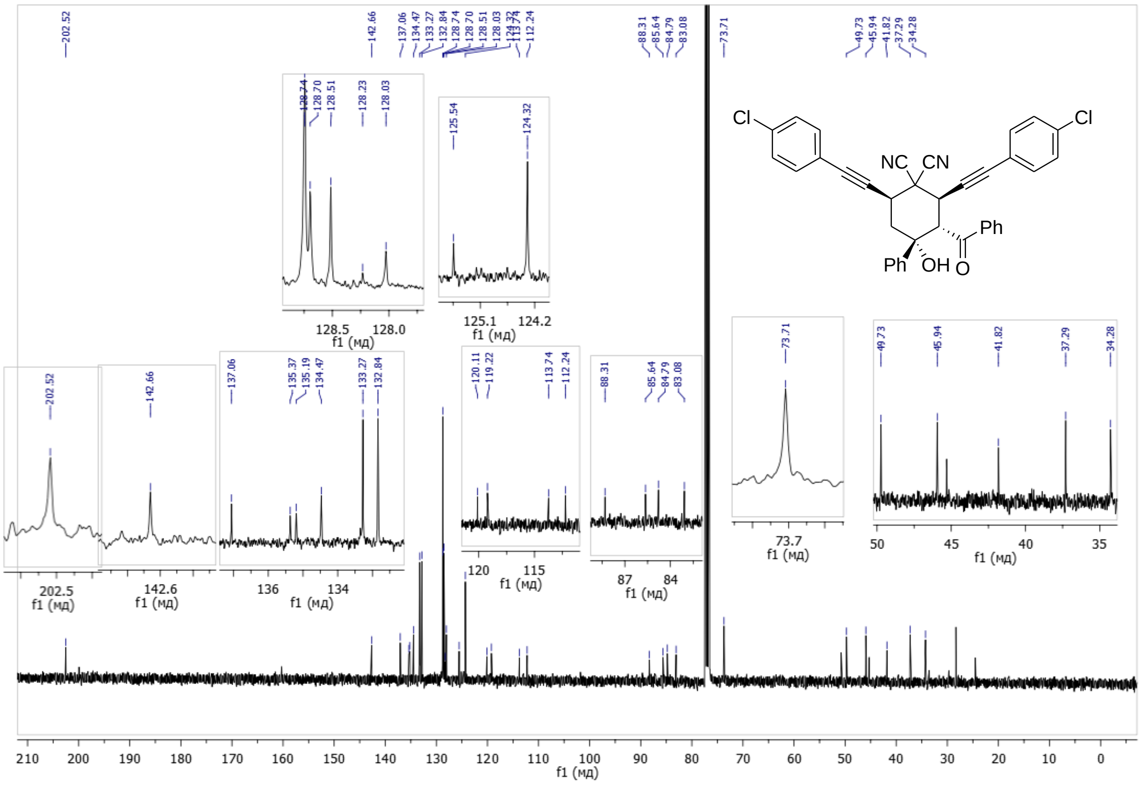

Figure S25. ^{13}C NMR spectrum of the compound **2e** ($CDCl_3$, 100 MHz).

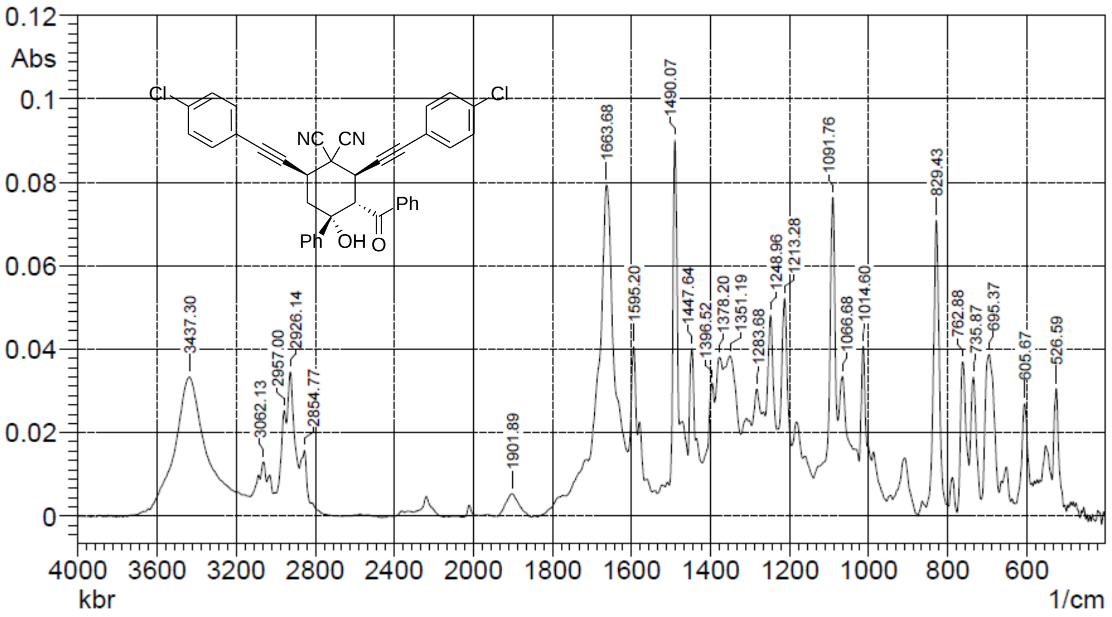

Figure S26. IR of the compound **2e** (KBr).

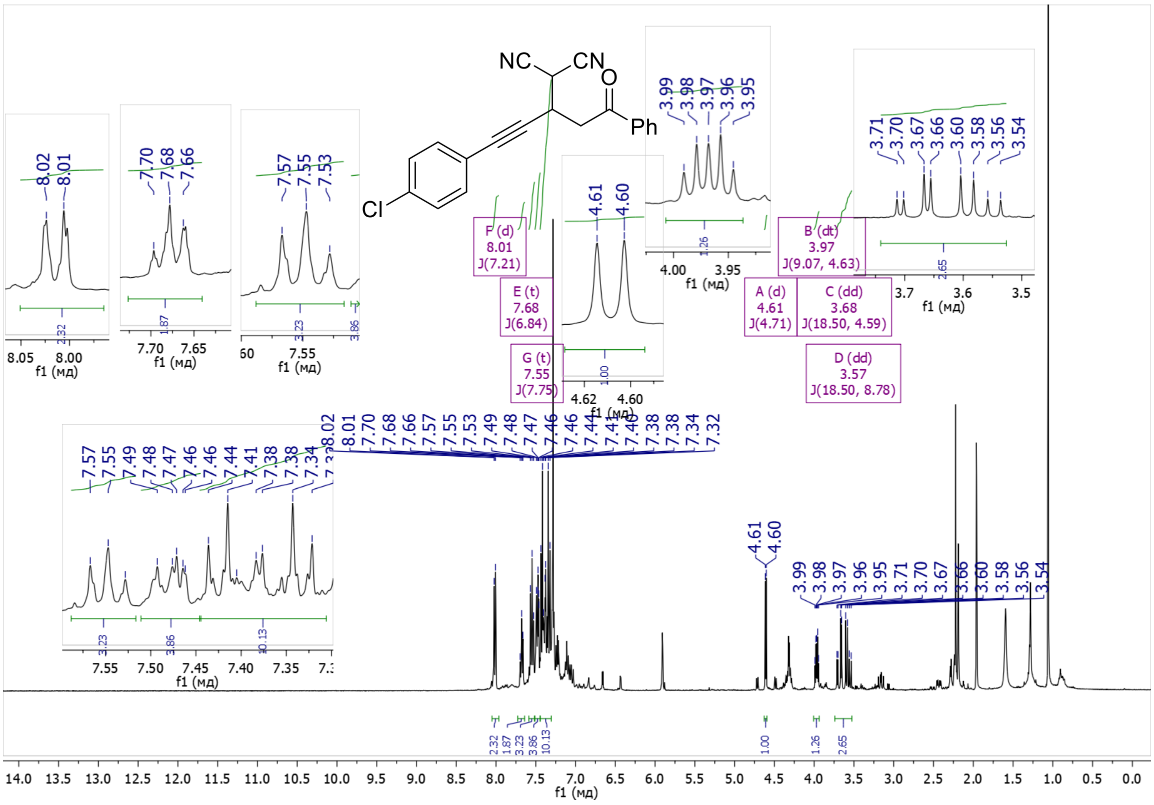

Figure S27. ^{1}H NMR spectrum of the compound **3e** ($CDCl_3$, 400 MHz).

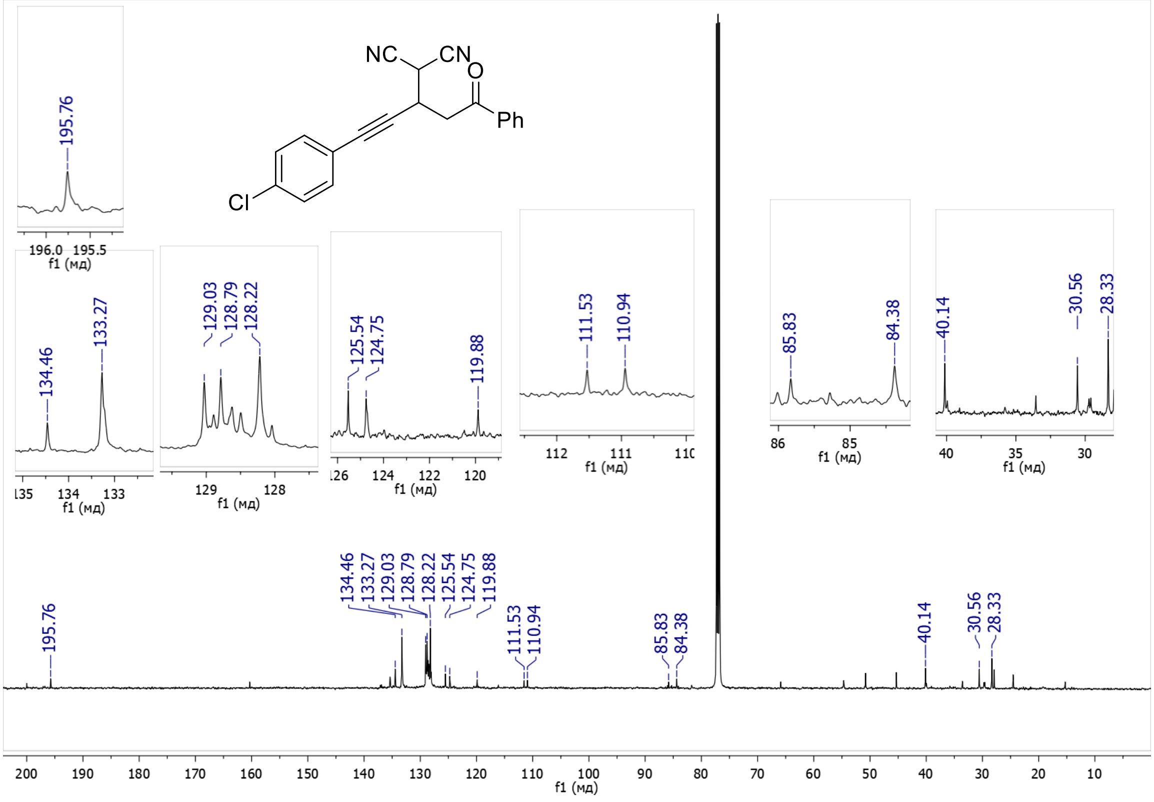

Figure S28. ^{13}C NMR spectrum of the compound **3e** ($CDCl_3$, 100 MHz).

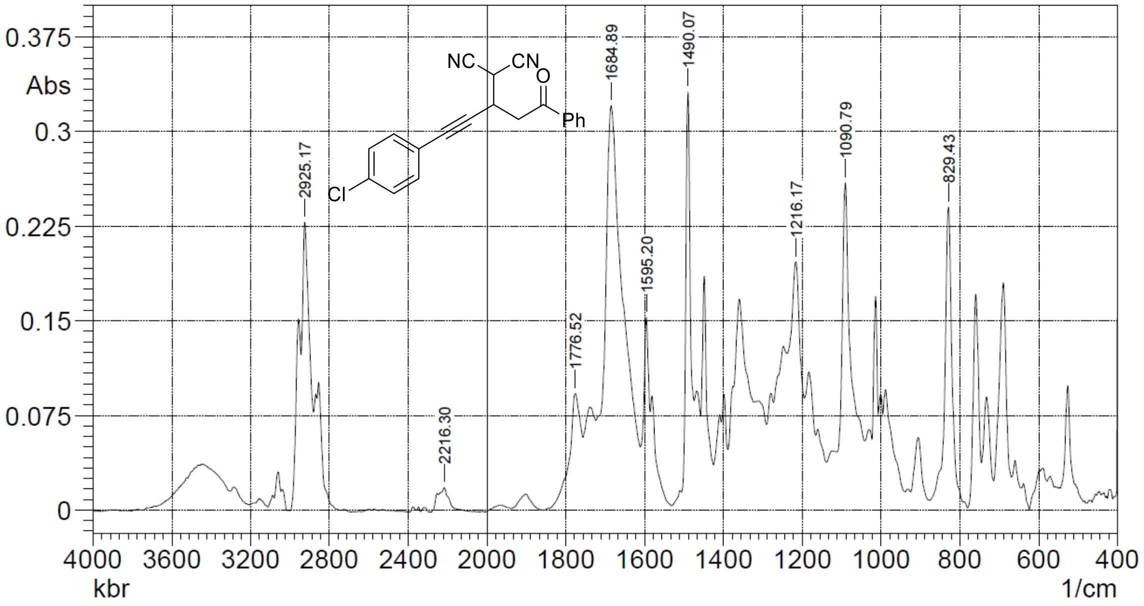

Figure S29. IR of the compound **3e** (KBr).

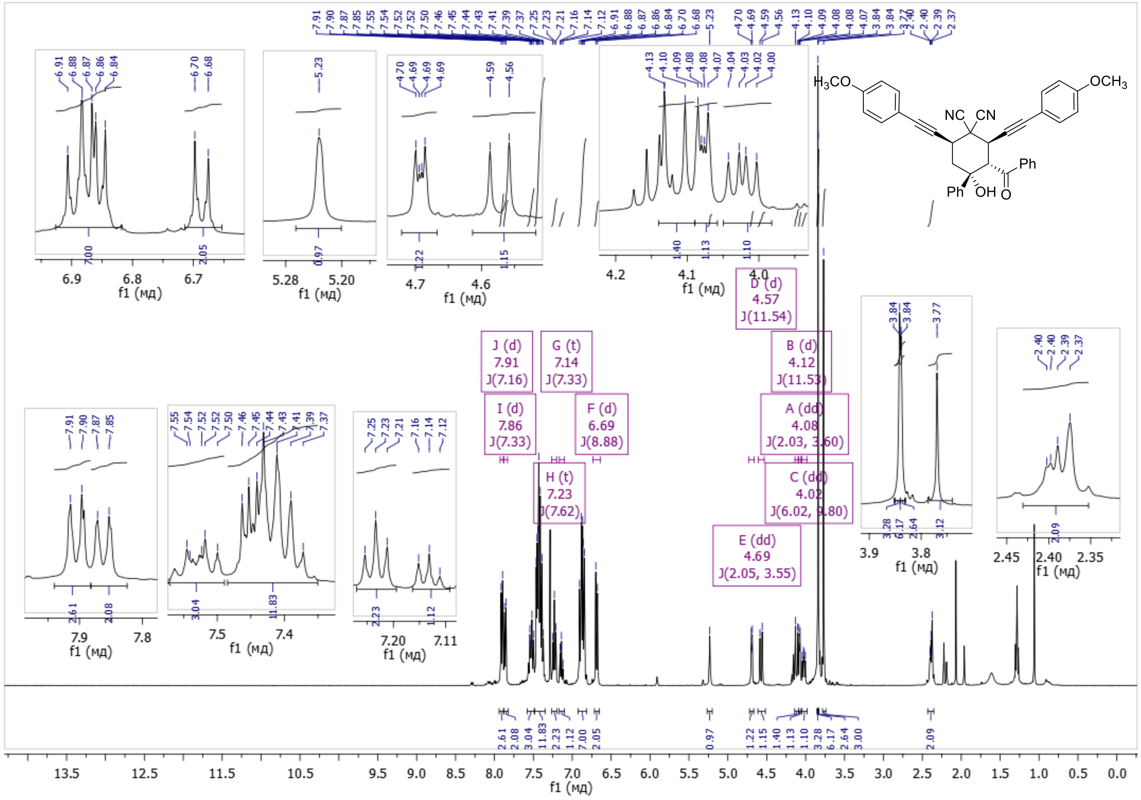

Figure S30. ^{1}H NMR spectrum of the compound **2f** ($CDCl_3$, 400 MHz).

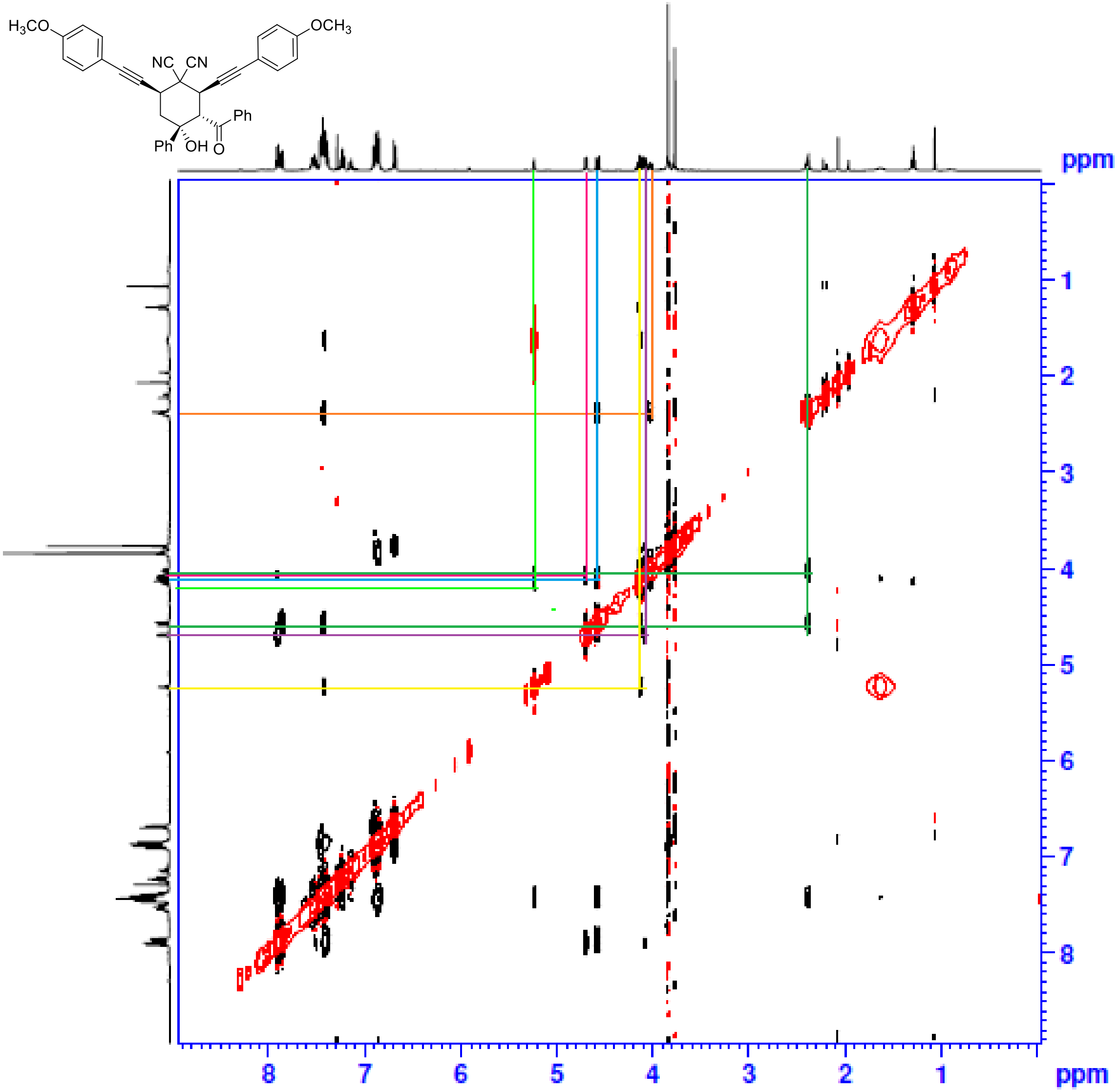

Figure S31. NOESY H-H NMR spectrum of the compound **2f** ($CDCl_3$).

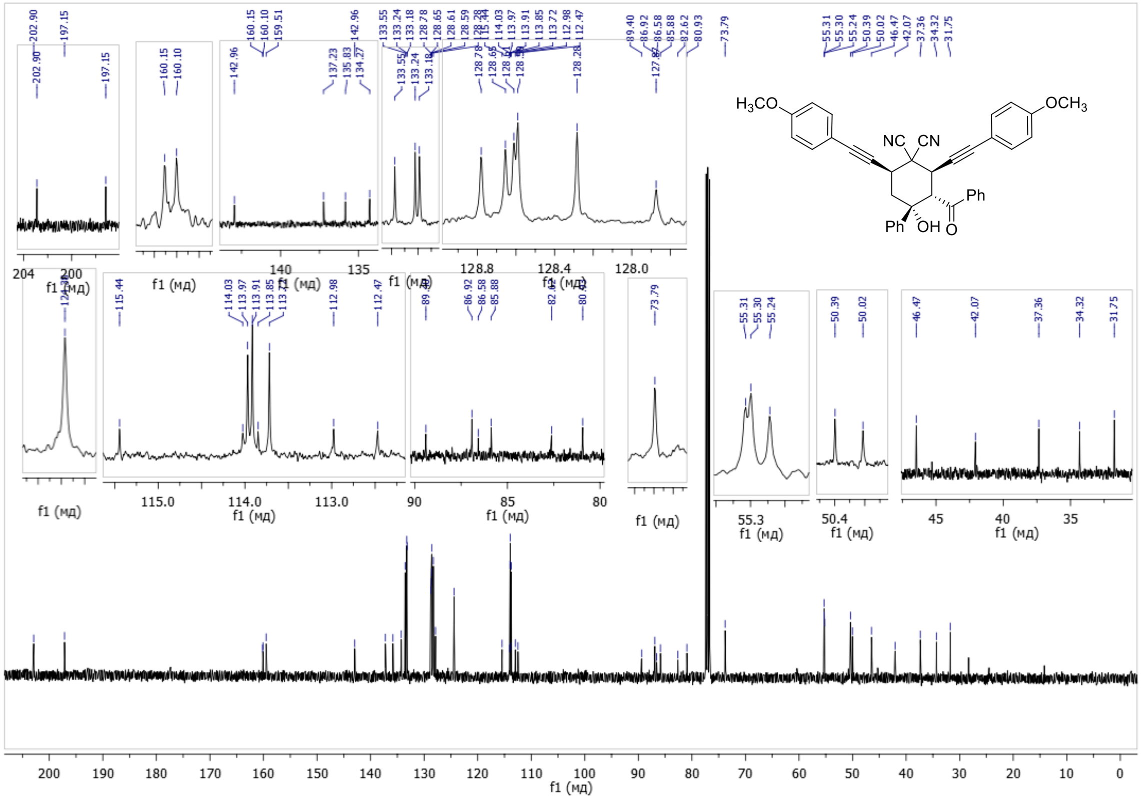

Figure S32. ^{13}C NMR spectrum of the compound **2f** ($CDCl_3$, 100 MHz).

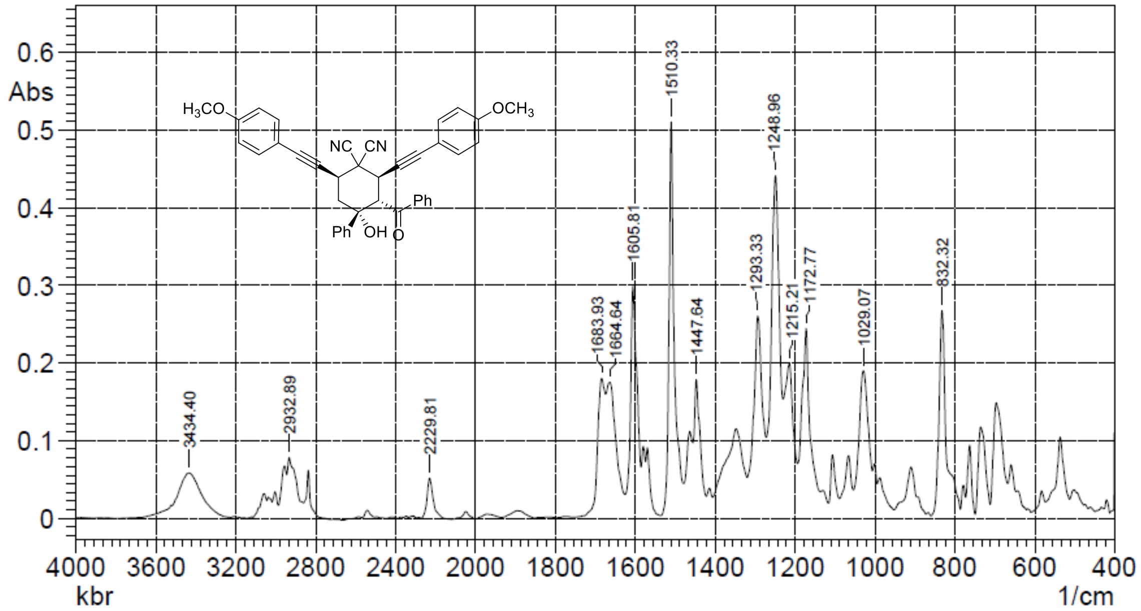

Figure S33. IR of the compound **2f** (KBr).

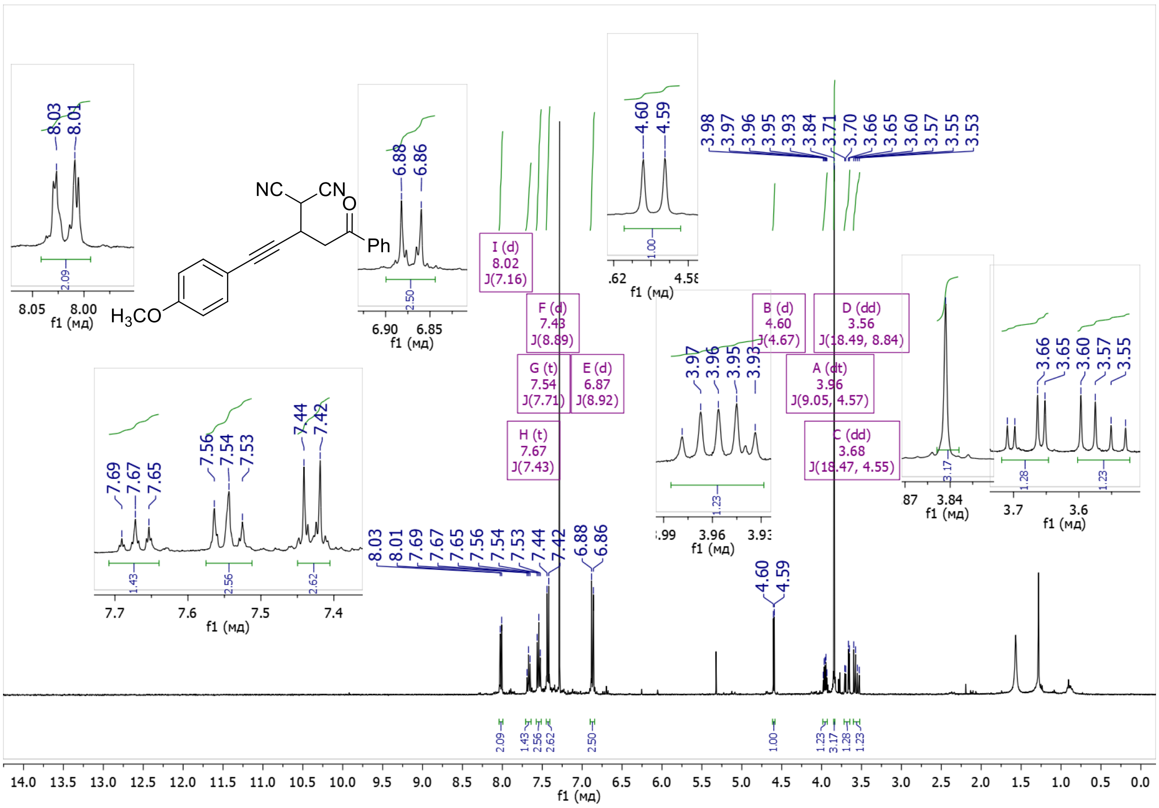

Figure S34. ^{1}H NMR spectrum of the compound **3f** ($CDCl_3$, 400 MHz).

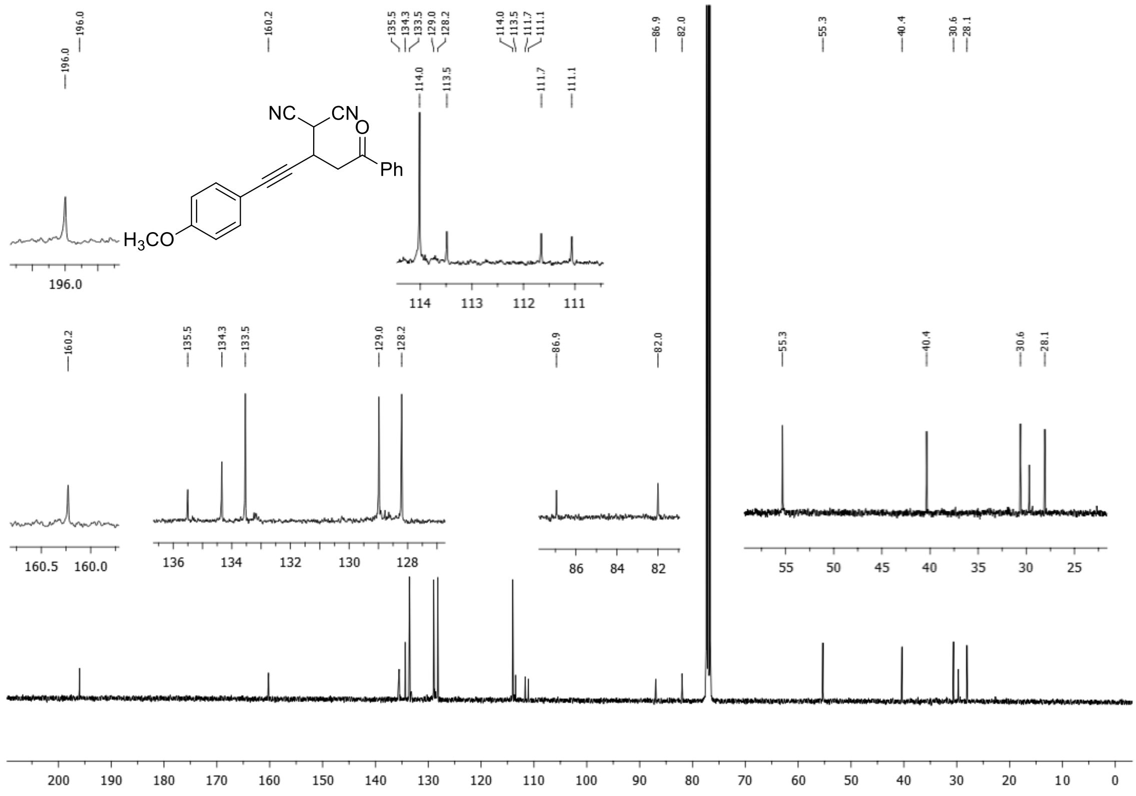

Figure S35. ^{13}C NMR spectrum of the compound **3f** ($CDCl_3$, 100 MHz).

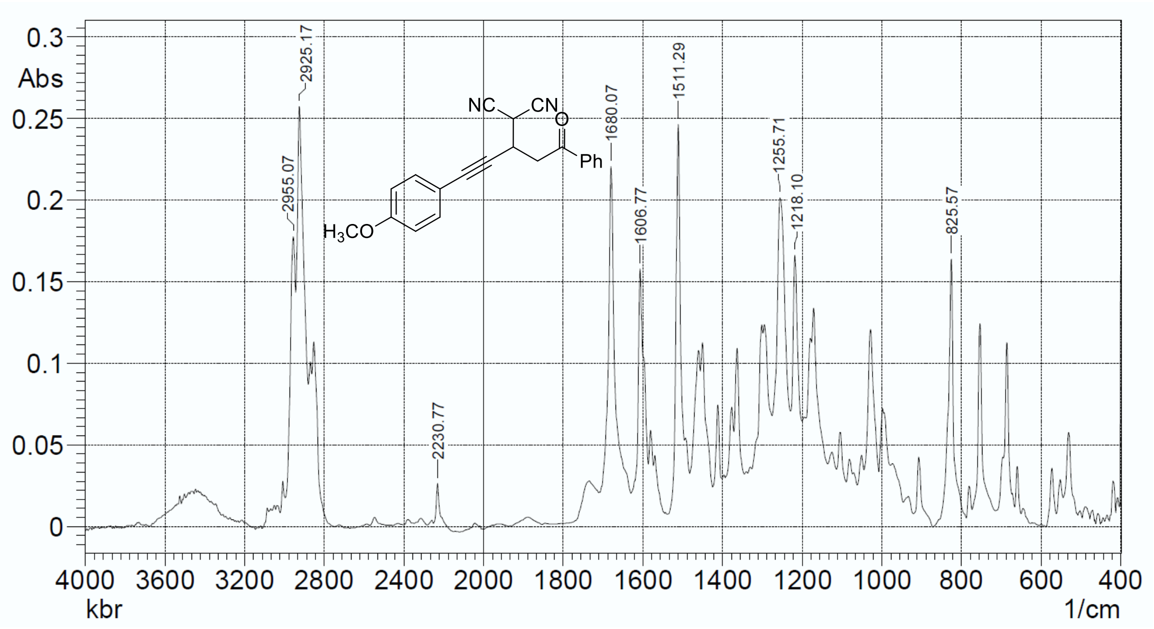

Figure S36. IR of the compound **3f** (KBr).

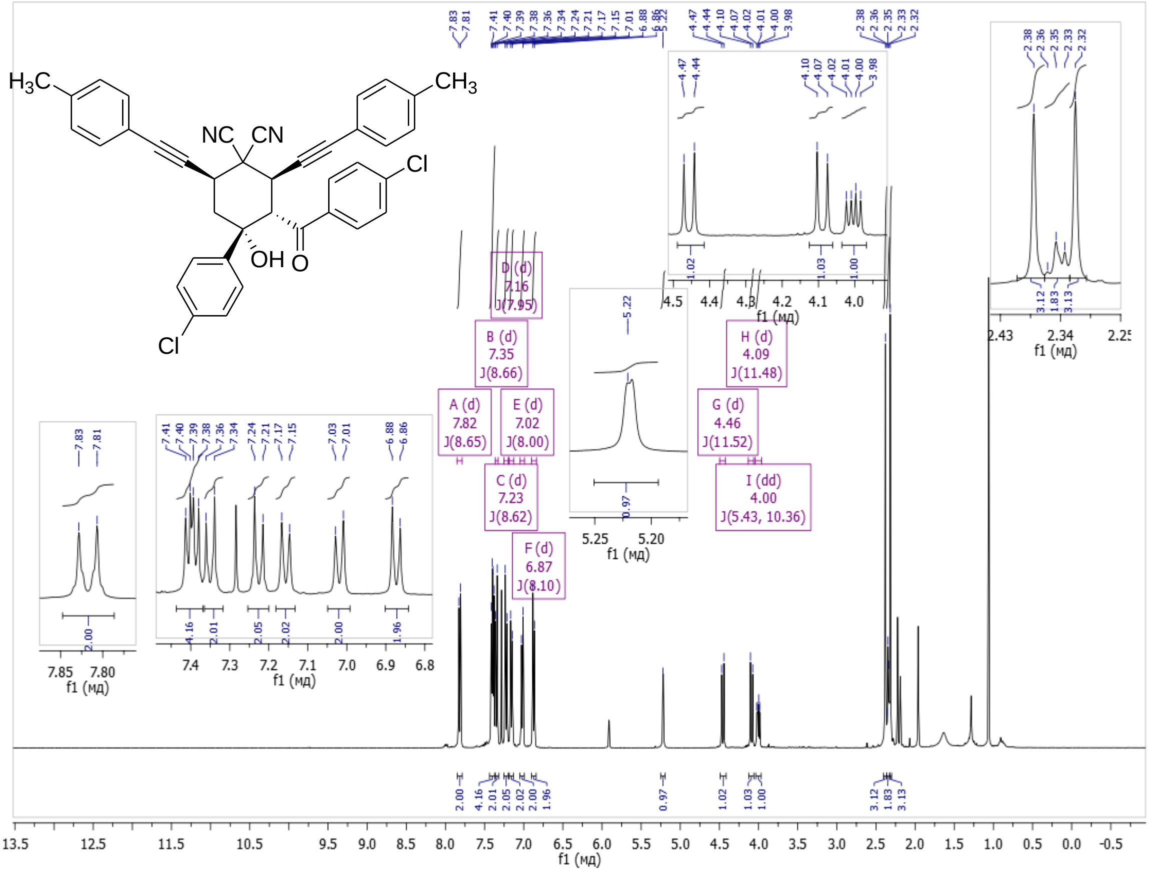

Figure S37. ^{1}H NMR spectrum of the compound **2g** ($CDCl_3$, 400 MHz).

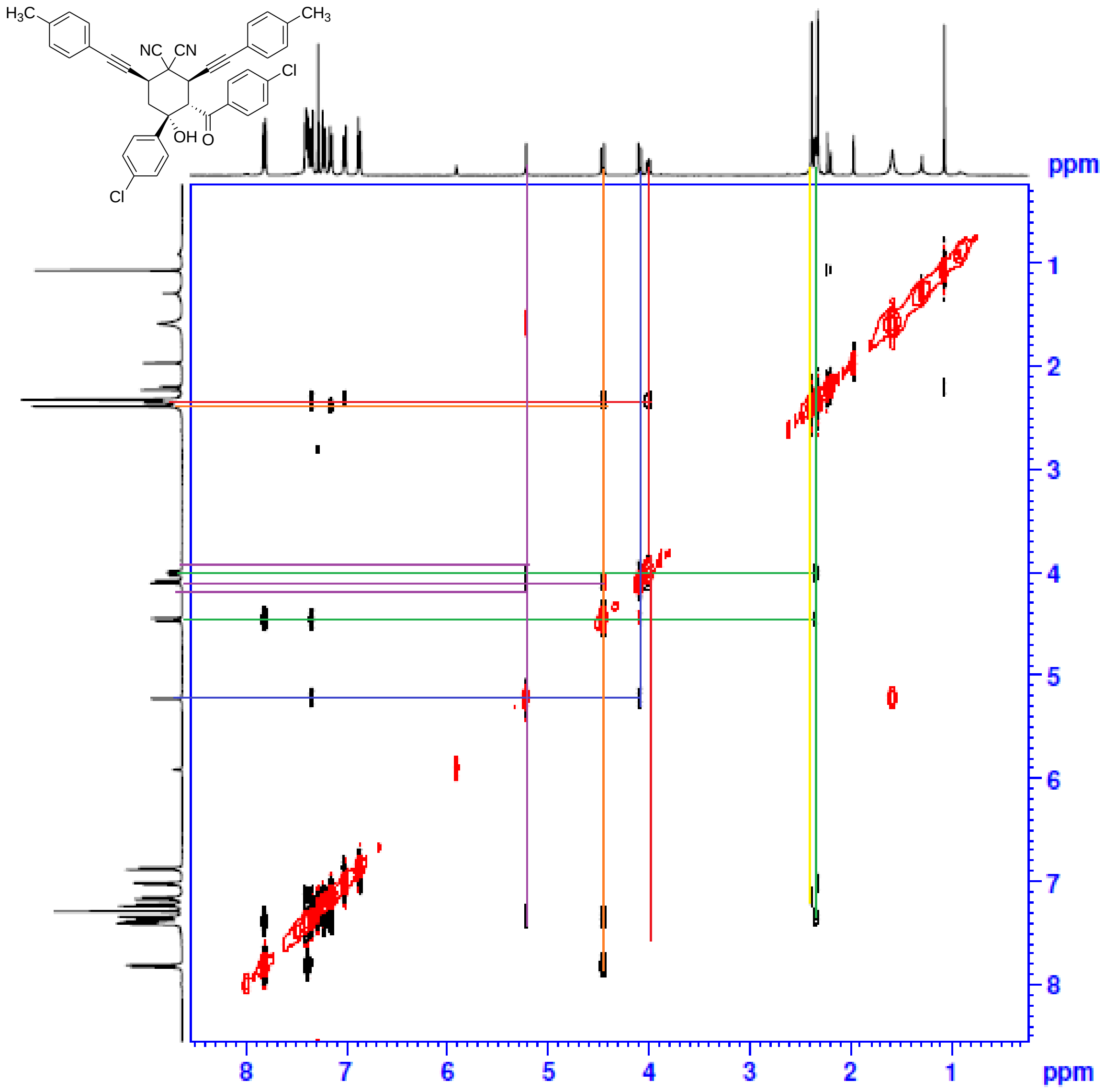

Figure S38. NOESY H-H NMR spectrum of the compound **2g** ($CDCl_3$).

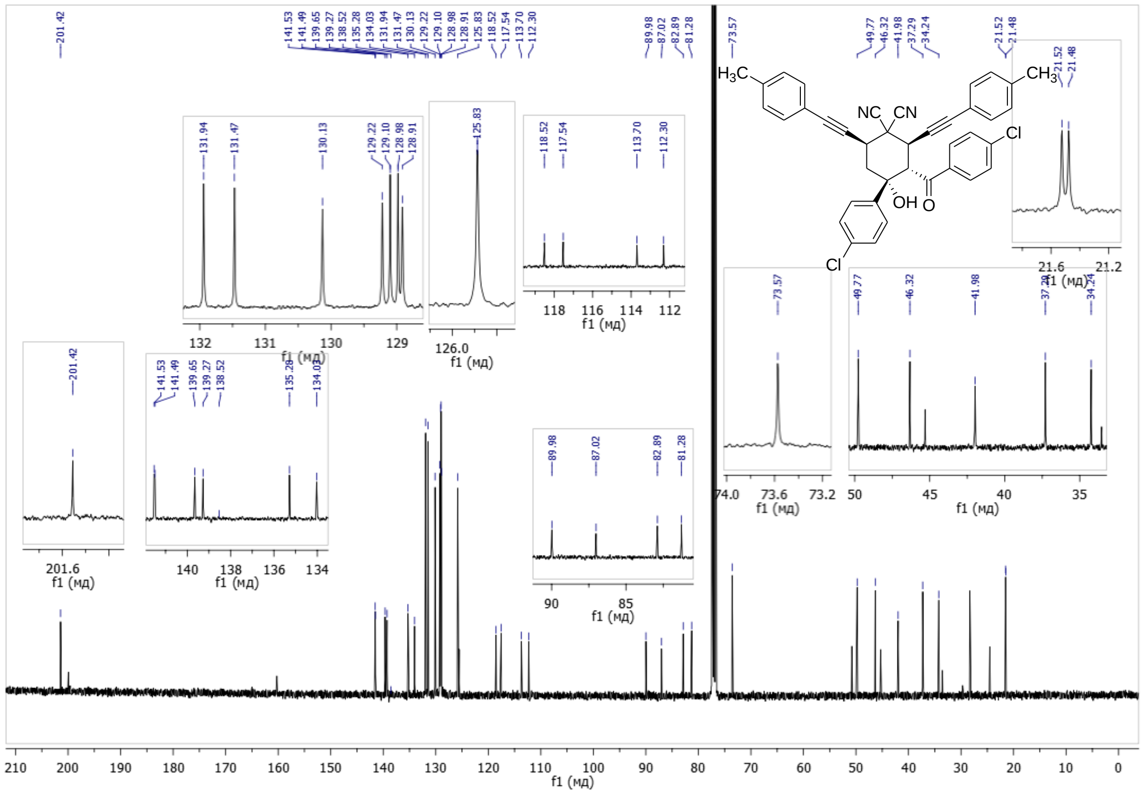

Figure S39. ^{13}C NMR spectrum of the compound **2g** ($CDCl_3$, 100 MHz).

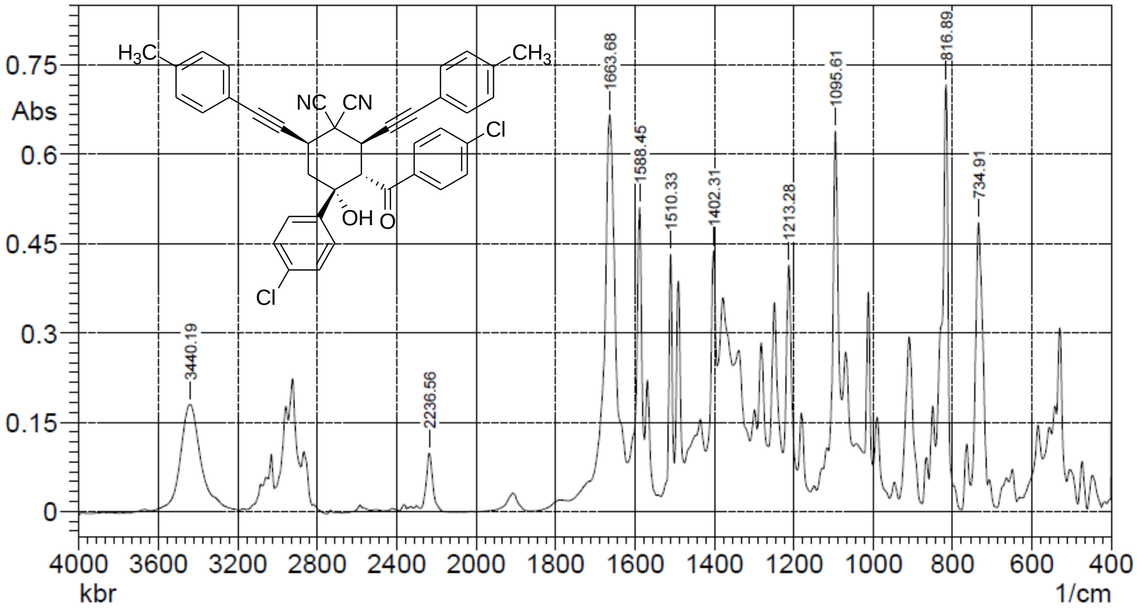

Figure S40. IR of the compound **2g** (KBr).

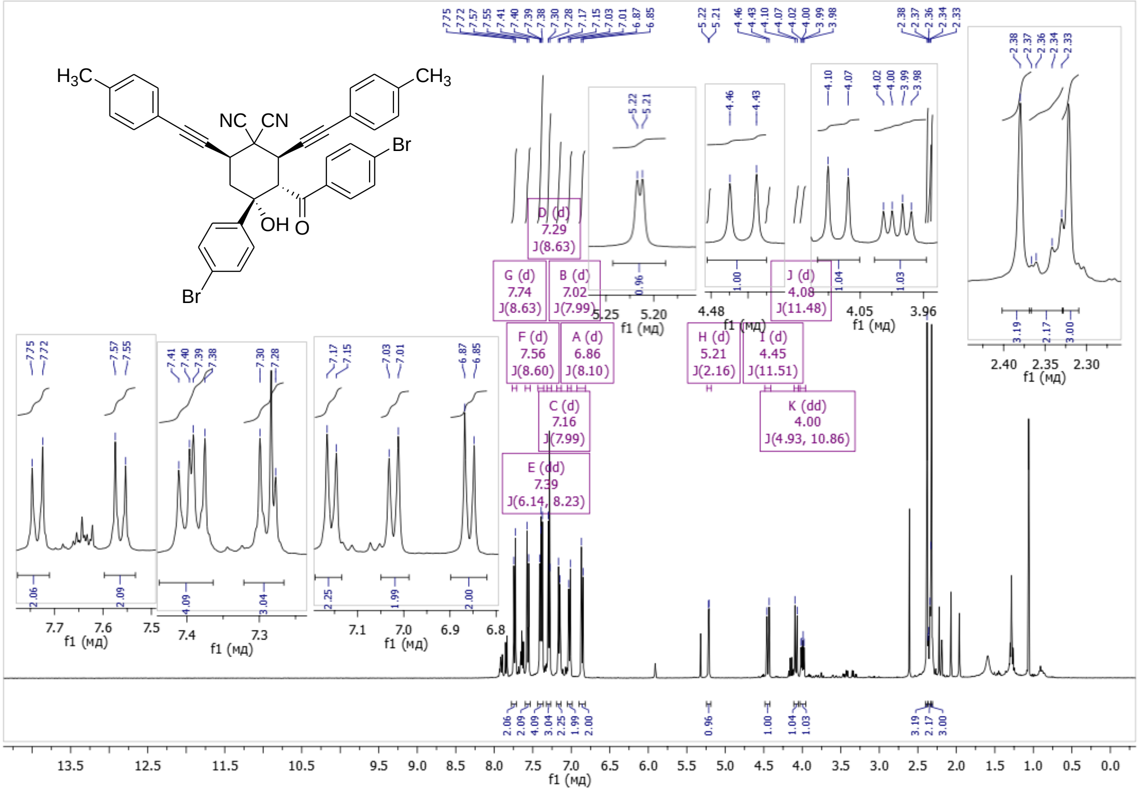

Figure S41. ^{1}H NMR spectrum of the compound **2h** ($CDCl_3$, 400 MHz).

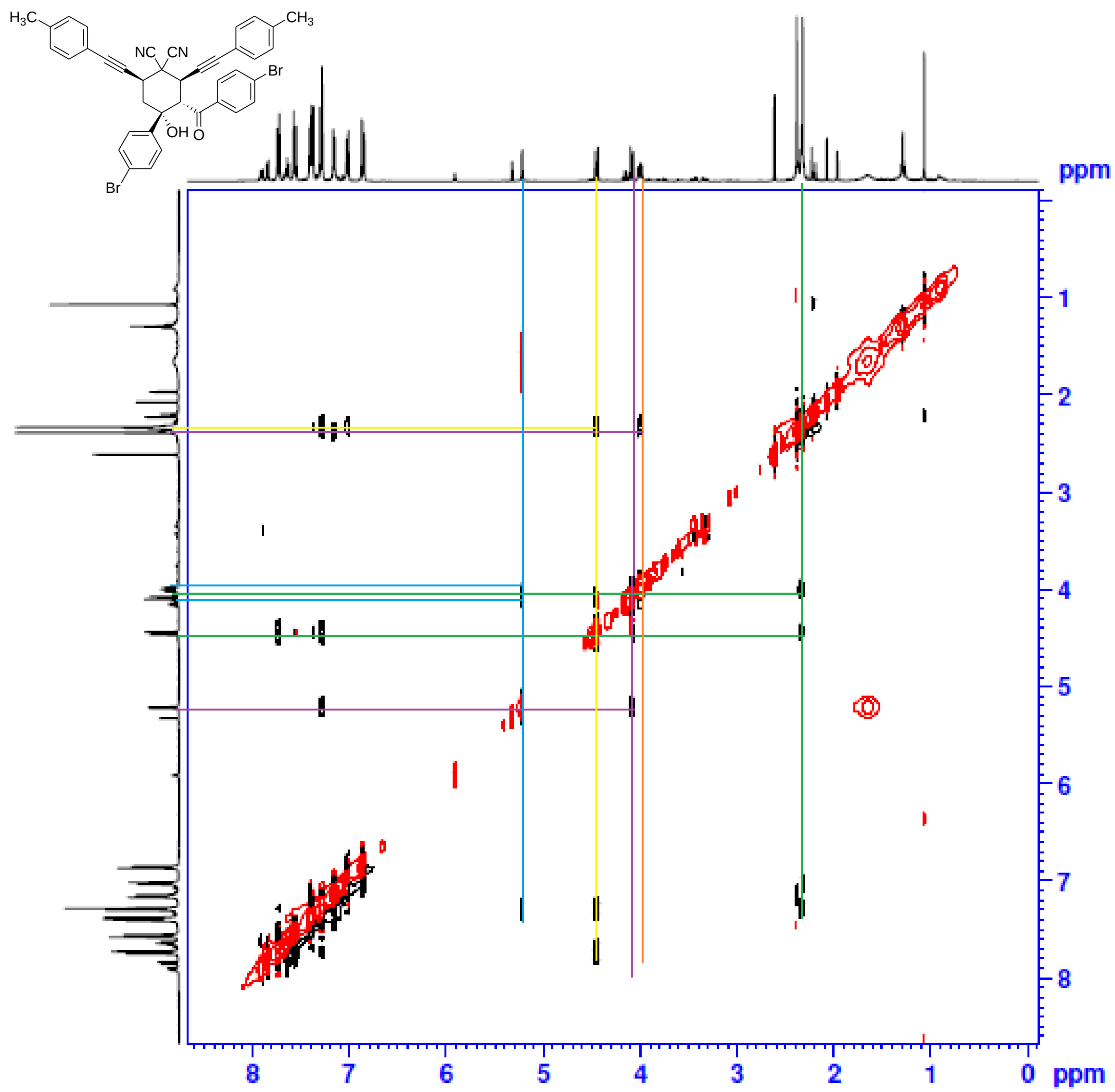

Figure S42. NOESY H-H NMR spectrum of the compound **2h** ($CDCl_3$).

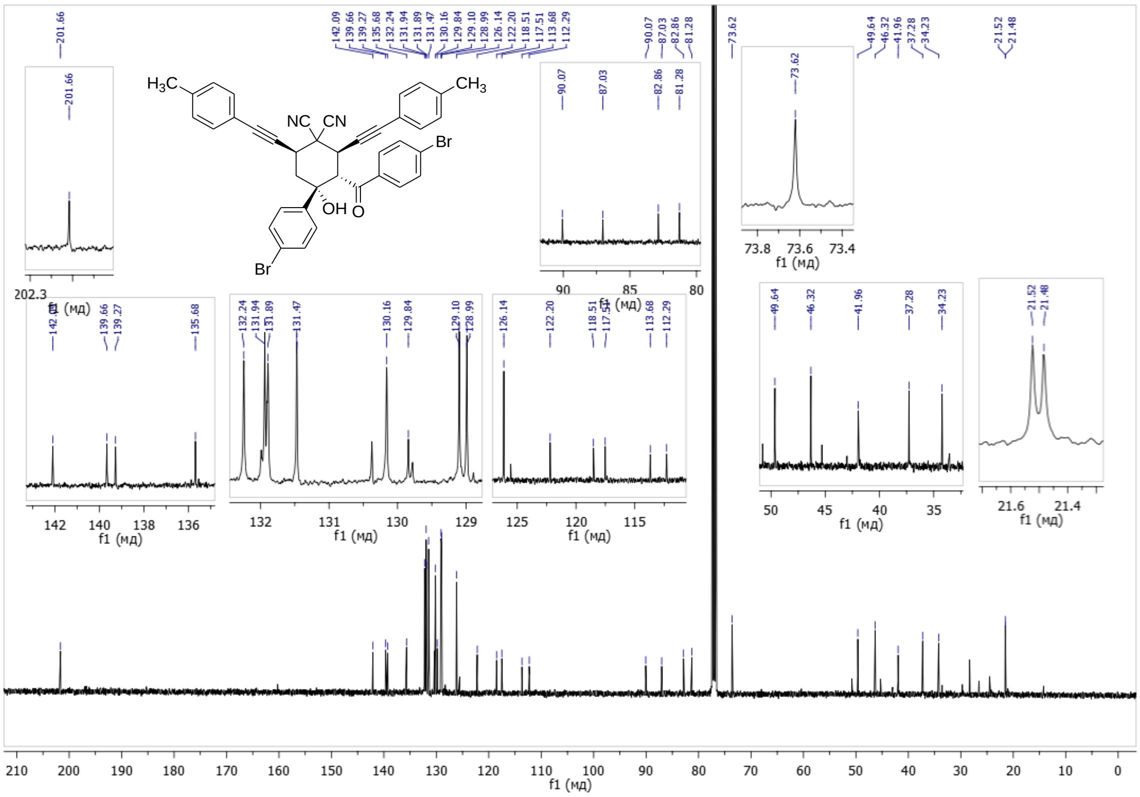

Figure S43. ^{13}C NMR spectrum of the compound **2h** ($CDCl_3$, 100 MHz).

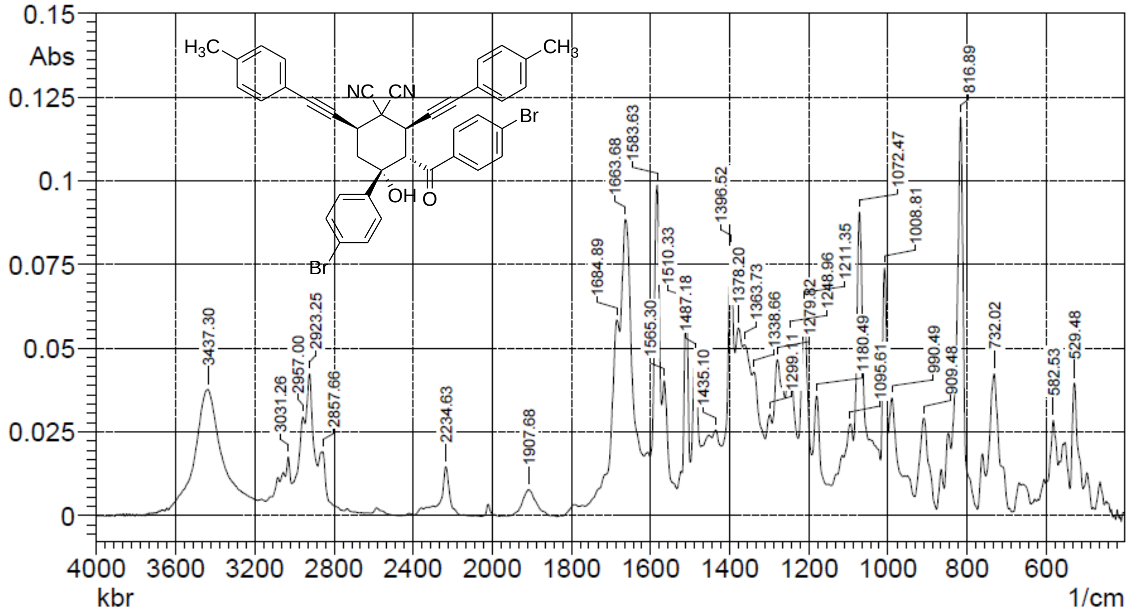

Figure S44. IR of the compound **2h** (KBr).

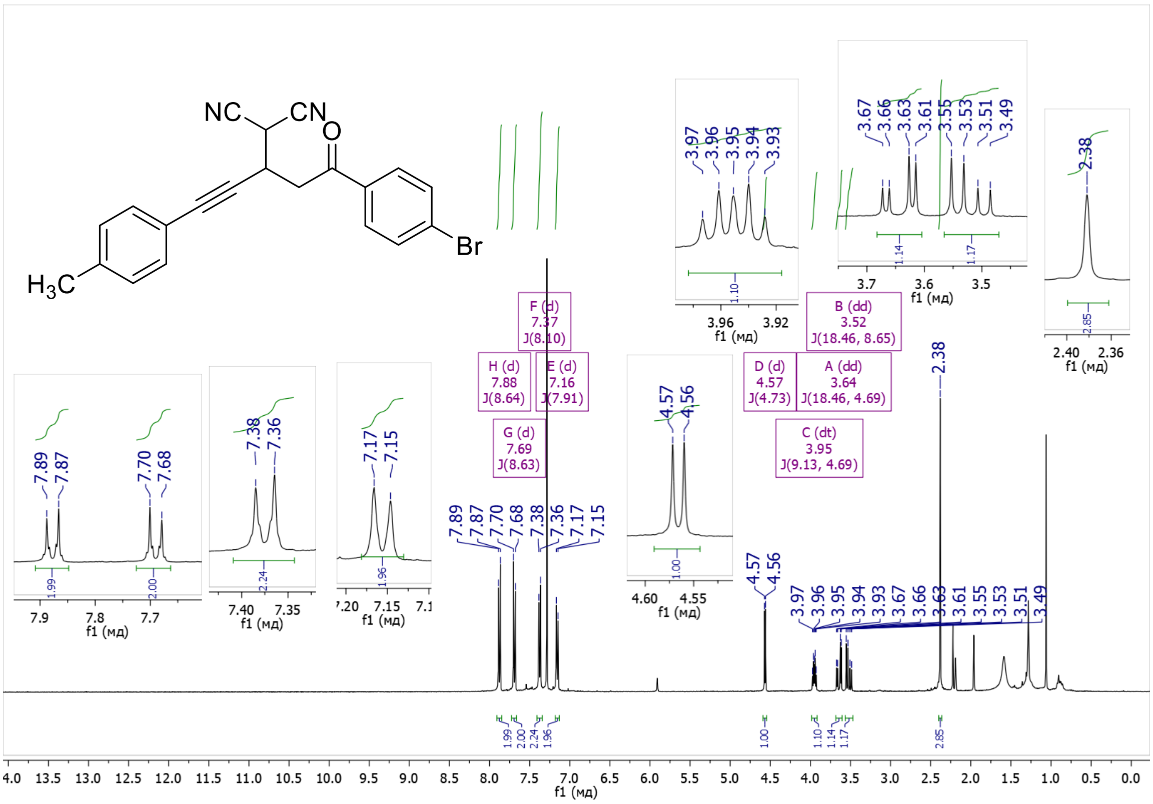

Figure S45. ^{1}H NMR spectrum of the compound **3h** ($CDCl_3$, 400 MHz).

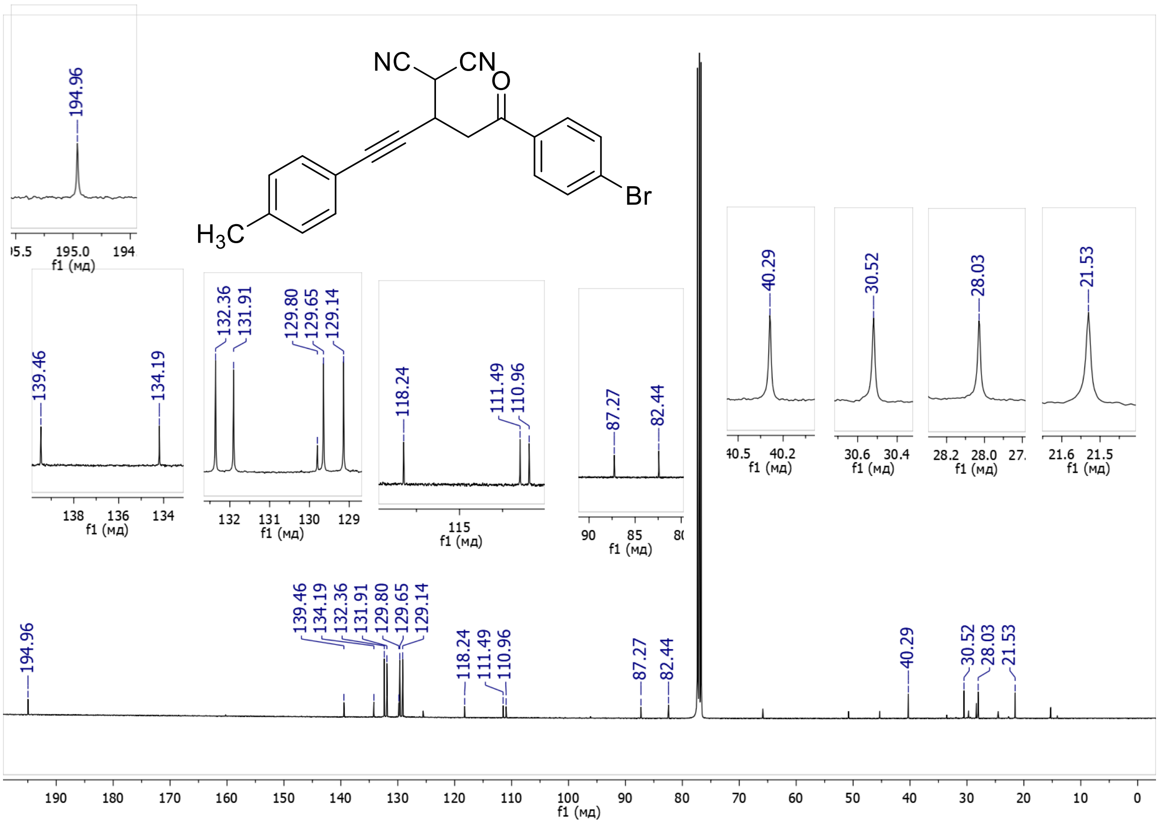

Figure S46. ^{13}C NMR spectrum of the compound **3h** ($CDCl_3$, 100 MHz).

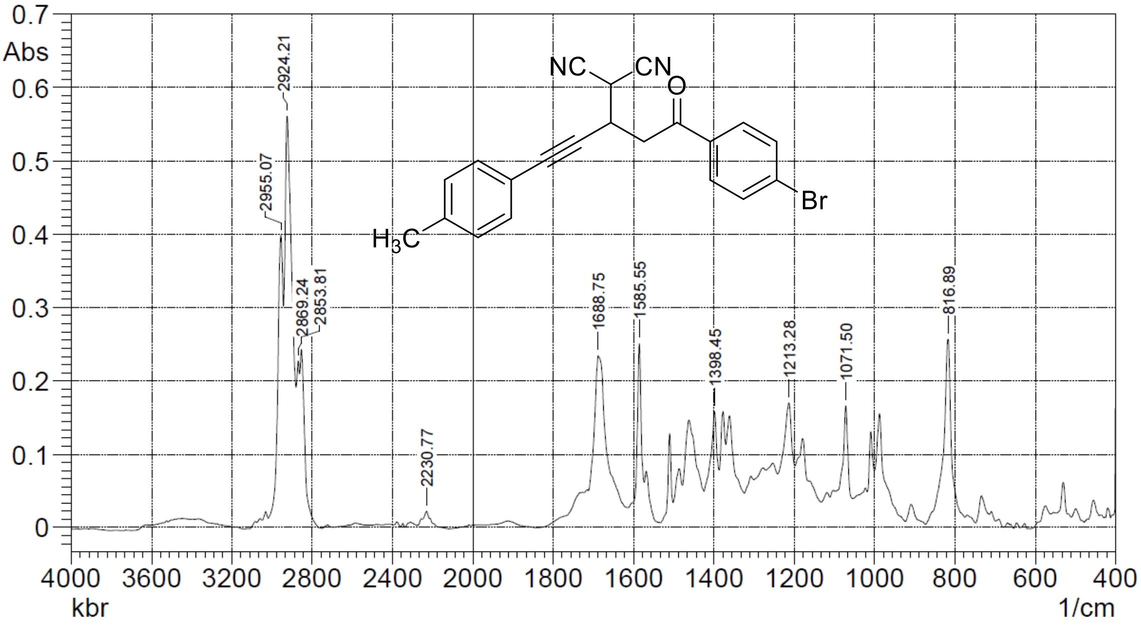

Figure S47. IR of the compound **3h** (KBr).

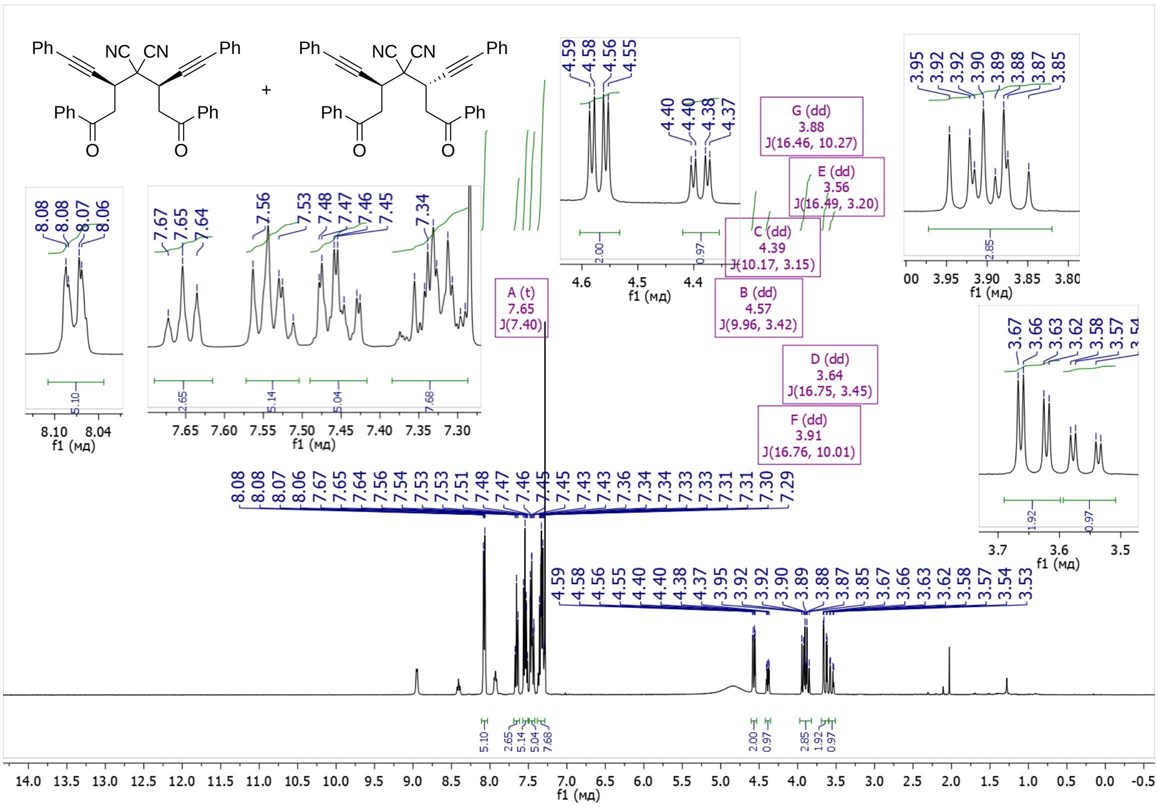

Figure S48. ^{1}H NMR spectrum of the compound **4a,b** ($CDCl_3$, 400 MHz).

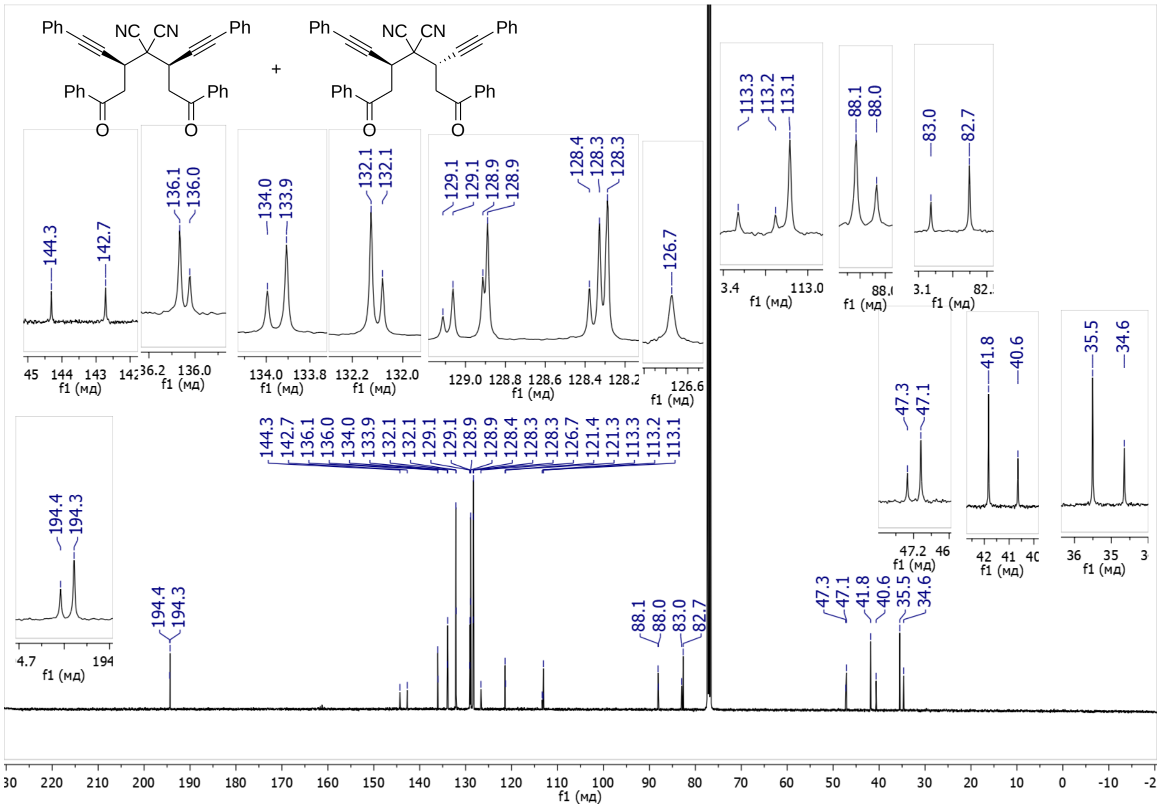

Figure S49. ^{13}C NMR spectrum of the compound **4a,b** ($CDCl_3$, 100 MHz).

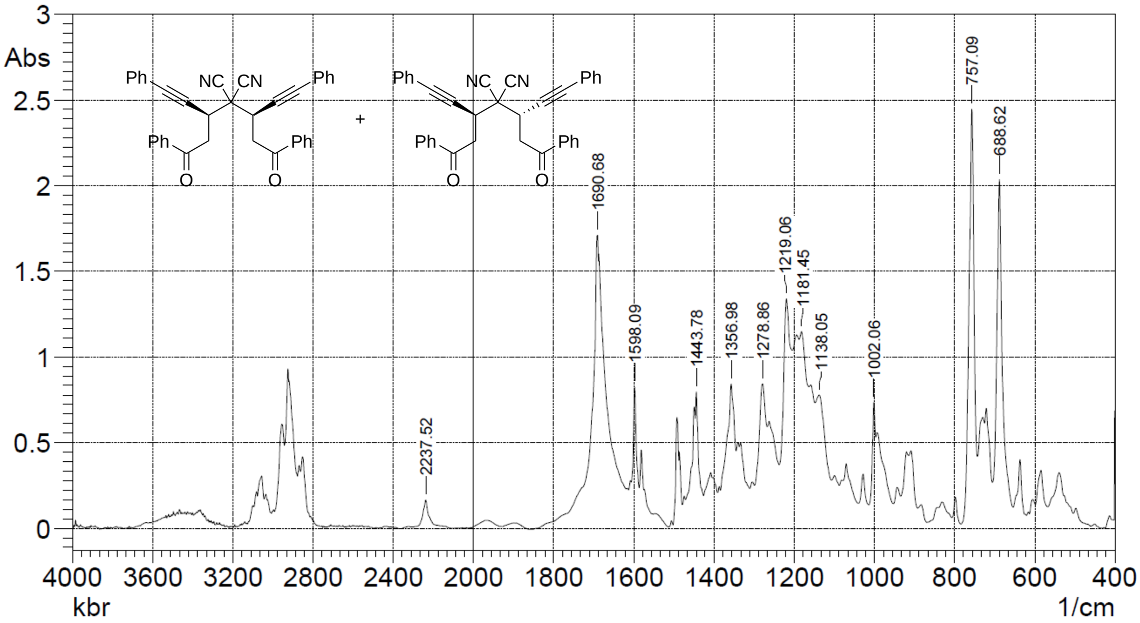

Figure S50. IR of the compound **4a,b** (KBr).

2. X-Ray data for compounds **2b**, **2e**, **3a**

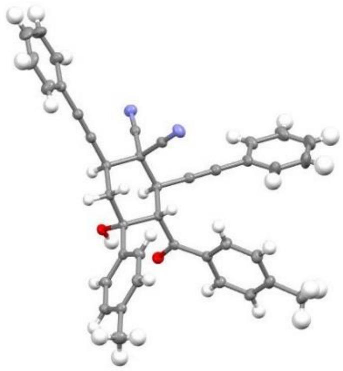

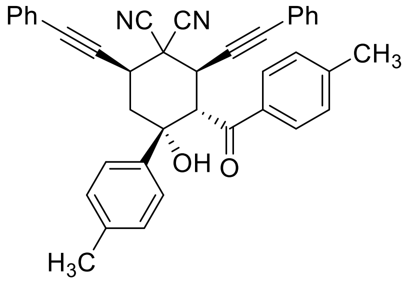

CCDC 1998357

Table S1 Crystal data and structure refinement for 2b.

Identification code	2b
Empirical formula	$C_{39}H_{30}N_2O_2$
Formula weight	558.65
Temperature/K	101.7(7)
Crystal system	tetragonal
Space group	$I4_1/a$
a/Å	22.45227(12)
b/Å	22.45227(12)
c/Å	24.6012(2)
α/°	90
β/°	90
γ/°	90
Volume/Å^3	12401.59(17)
Z	16
ρ_{calc}g/cm^3	1.197
μ/mm^{-1}	0.578
F(000)	4704.0
Crystal size/mm^3	0.3 × 0.3 × 0.3
Radiation	CuKα (λ = 1.54184)
2Θ range for data collection/°	5.328 to 140.738

Index ranges	$-23 \leq h \leq 26$, $-27 \leq k \leq 27$, $-29 \leq l \leq 30$
Reflections collected	25291
Independent reflections	5899 [R_{int} = 0.0442, R_{sigma} = 0.0300]
Data/restraints/parameters	5899/0/395
Goodness-of-fit on F^2	1.023
Final R indexes [I>=2σ (I)]	R_1 = 0.0399, wR_2 = 0.1050
Final R indexes [all data]	R_1 = 0.0433, wR_2 = 0.1078
Largest diff. peak/hole / e $Å^{-3}$	0.23/-0.21

Table S2 Fractional Atomic Coordinates (×10^4) and Equivalent Isotropic Displacement Parameters ($Å^2$ ×10^3) for 2b. U_{eq} is defined as 1/3 of of the trace of the orthogonalised U_{IJ} tensor.

Atom	*x*	*y*	*z*	U(eq)
O32	5617.5(4)	6752.1(4)	1127.2(3)	20.11(19)
O15	6351.5(4)	7332.4(3)	402.8(3)	23.9(2)
N36	7187.3(5)	4842.1(5)	1071.4(5)	31.1(3)
C6	6428.4(5)	6304.0(5)	593.4(5)	18.6(2)
C25	7986.5(5)	6438.4(5)	831.1(5)	24.8(3)
C37	6844.5(5)	6809.1(5)	-295.5(5)	20.9(2)
N34	7327.2(5)	6010.2(5)	2405.6(4)	28.9(2)
C33	7080.8(5)	5928.7(5)	2005.2(5)	21.9(3)
C40	7417.8(6)	6800.9(6)	-1312.7(5)	29.7(3)
C5	6838.7(5)	6360.5(5)	1099.9(5)	19.5(2)
C35	6988.5(5)	5266.5(5)	1252.7(5)	21.9(2)
C38	7041.7(6)	6275.9(5)	-524.2(5)	25.3(3)
C8	4911.0(5)	6684.0(5)	213.6(5)	24.4(3)
C28	9622.9(6)	6720.7(7)	889.0(7)	41.5(4)
C24	7469.4(5)	6415.1(5)	951.6(5)	22.2(2)

Table S2 Fractional Atomic Coordinates (×10^4) and Equivalent Isotropic Displacement Parameters (Å² ×10³) for 2b. U_{eq} is defined as 1/3 of of the trace of the orthogonalised U_{IJ} tensor.

Atom	*x*	*y*	*z*	U(eq)
C2	5695.1(5)	5706.6(5)	1126.2(5)	19.5(2)
C26	8606.0(6)	6462.7(6)	681.6(5)	28.4(3)
C4	6745.9(5)	5820.1(5)	1495.9(5)	19.9(2)
C10	4522.2(5)	6184.9(6)	-583.6(5)	24.1(3)
C41	7226.2(6)	7332.6(6)	-1079.5(5)	28.8(3)
C14	6532.7(5)	6851.6(5)	234.3(5)	19.5(2)
C3	6072.2(5)	5749.0(5)	1644.0(5)	19.8(2)
C1	5762.9(5)	6268.5(5)	776.5(5)	19.1(2)
C12	5349.7(5)	5769.7(5)	-73.9(5)	24.6(3)
C27	9027.4(6)	6698.3(6)	1038.0(6)	31.5(3)
C42	6945.2(6)	7340.1(5)	-581.1(5)	24.9(3)
C30	9388.4(8)	6271.2(10)	37.6(7)	57.2(5)
C18	5811.5(5)	4373.8(5)	2709.6(5)	24.0(3)
C7	5337.9(5)	6242.7(5)	291.0(5)	19.5(2)
C11	4947.3(6)	5742.8(5)	-502.7(5)	26.3(3)
C9	4515.7(6)	6656.7(6)	-219.6(5)	27.0(3)
C17	5909.7(5)	4838.8(5)	2318.9(5)	22.9(3)
C13	4079.5(7)	6155.3(7)	-1042.9(6)	35.2(3)
C39	7322.9(6)	6274.1(6)	-1027.5(5)	29.8(3)
C29	9803.0(7)	6505.3(9)	390.2(8)	51.6(5)
C16	5982.6(5)	5243.0(5)	2009.2(5)	22.2(3)
C23	5388.1(7)	3931.9(6)	2616.3(6)	34.9(3)
C19	6129.4(6)	4372.6(7)	3195.3(6)	35.4(3)
C43	7714.2(8)	6805.9(8)	-1862.7(6)	43.6(4)
C22	5287.1(8)	3497.8(7)	3003.5(7)	45.6(4)
C20	6019.3(7)	3936.2(8)	3581.0(7)	46.5(4)

Table S2 Fractional Atomic Coordinates (×10^{4}) and Equivalent Isotropic Displacement Parameters (Å^{2}×10^{3}) for 2b. U_{eq} is defined as 1/3 of of the trace of the orthogonalised U_{IJ} tensor.

**Atom**	*x*	*y*	*z*	**U(eq)**
C21	5599.0(8)	3500.5(7)	3485.2(7)	46.2(4)
C31	8790.4(7)	6249.4(9)	177.9(6)	45.0(4)

Table S3 Anisotropic Displacement Parameters (Å^{2}×10^{3}) for 2b. The Anisotropic displacement factor exponent takes the form: $-2\pi^2[h^2a^{*2}U_{11}+2hka^*b^*U_{12}+\ldots]$.

**Atom**	U_{11}	U_{22}	U_{33}	U_{23}	U_{13}	U_{12}
O32	20.5(4)	17.4(4)	22.4(4)	-1.6(3)	1.6(3)	0.4(3)
O15	27.9(4)	17.1(4)	26.8(4)	-0.5(3)	2.2(3)	0.1(3)
N36	27.8(6)	26.6(6)	39.0(6)	-2.9(5)	0.3(5)	3.7(4)
C6	17.8(5)	17.1(5)	20.7(6)	-0.3(4)	0.6(4)	0.0(4)
C25	22.2(6)	26.1(6)	26.1(6)	3.6(5)	-0.2(5)	-1.3(5)
C37	19.5(5)	21.9(6)	21.3(6)	0.1(4)	-1.5(4)	-2.6(4)
N34	26.9(5)	31.7(6)	28.0(6)	3.1(4)	-3.5(4)	-4.9(4)
C33	19.1(5)	20.6(6)	25.9(6)	3.4(4)	1.5(5)	-1.3(4)
C40	28.3(6)	36.5(7)	24.2(6)	0.5(5)	2.3(5)	-1.7(5)
C5	19.0(5)	17.2(5)	22.4(6)	1.5(4)	0.2(4)	-1.5(4)
C35	18.0(5)	23.0(6)	24.8(6)	2.5(5)	-0.8(4)	-1.1(4)
C38	28.7(6)	21.1(6)	26.0(6)	0.8(5)	2.7(5)	-1.5(5)
C8	26.0(6)	22.8(6)	24.3(6)	-3.1(5)	-0.9(5)	3.5(5)
C28	21.4(7)	39.8(8)	63.2(10)	11.6(7)	-2.1(6)	-2.7(6)
C24	21.3(6)	22.2(6)	23.1(6)	1.8(4)	-1.8(5)	-1.8(4)
C2	16.6(5)	18.5(5)	23.5(6)	0.0(4)	1.5(4)	-0.9(4)
C26	21.1(6)	31.9(7)	32.2(7)	9.0(5)	3.8(5)	-0.1(5)
C4	17.8(5)	19.1(5)	22.8(6)	1.5(4)	-0.4(4)	0.0(4)
C10	21.6(6)	27.0(6)	23.8(6)	2.4(5)	-0.4(5)	-4.9(5)
C41	31.9(7)	27.1(6)	27.3(6)	6.1(5)	1.3(5)	-4.8(5)

Table S3 Anisotropic Displacement Parameters (Å^2 ×10^3) for 2b. The Anisotropic displacement factor exponent takes the form: -2π^2[h^{2}a*^{2}U$_{11}$+2hka*b*U$_{12}$+…].

Atom	U_{11}	U_{22}	U_{33}	U_{23}	U_{13}	U_{12}
C14	17.1(5)	18.3(5)	23.0(6)	-0.7(4)	-3.0(4)	-1.3(4)
C3	17.4(5)	18.8(5)	23.3(6)	0.6(4)	1.9(4)	-0.4(4)
C1	17.6(5)	16.9(5)	22.7(6)	-1.3(4)	0.7(4)	0.4(4)
C12	23.7(6)	20.8(6)	29.2(6)	-1.3(5)	-1.5(5)	1.7(5)
C27	22.3(6)	27.6(6)	44.7(8)	4.3(6)	0.3(5)	-0.4(5)
C42	27.3(6)	21.3(6)	26.2(6)	0.3(5)	-0.2(5)	-2.6(5)
C30	35.5(9)	98.3(15)	37.9(9)	10.8(9)	15.1(7)	11.1(9)
C18	23.1(6)	22.9(6)	25.9(6)	3.8(5)	3.4(5)	1.5(5)
C7	17.6(5)	19.9(5)	20.9(6)	1.5(4)	1.8(4)	-2.3(4)
C11	29.5(6)	22.1(6)	27.4(6)	-5.2(5)	-1.5(5)	-3.3(5)
C9	25.5(6)	27.4(6)	28.3(6)	0.8(5)	-2.6(5)	6.5(5)
C17	20.4(6)	23.0(6)	25.5(6)	1.5(5)	0.6(4)	-0.4(4)
C13	36.7(7)	35.2(7)	33.6(7)	-2.1(6)	-9.5(6)	-1.1(6)
C39	33.9(7)	27.1(6)	28.4(7)	-3.7(5)	5.7(5)	0.9(5)
C29	21.3(7)	72.3(12)	61.3(11)	24.7(9)	11.8(7)	2.7(7)
C16	18.1(5)	23.7(6)	24.7(6)	-0.1(5)	0.2(4)	-0.4(4)
C23	39.5(8)	35.3(7)	29.9(7)	1.9(6)	1.4(6)	-11.3(6)
C19	28.2(7)	45.3(8)	32.6(7)	9.8(6)	-3.4(5)	-5.9(6)
C43	50.5(9)	48.5(9)	31.7(8)	5.7(6)	11.9(7)	4.2(7)
C22	57.1(10)	33.6(8)	46.1(9)	4.1(7)	12.0(7)	-16.1(7)
C20	37.9(8)	66.2(11)	35.4(8)	23.3(7)	-3.5(6)	2.6(7)
C21	52.2(9)	41.2(8)	45.2(9)	23.7(7)	13.2(7)	3.7(7)
C31	29.7(7)	74.7(12)	30.7(8)	3.6(7)	3.4(6)	2.0(7)

Table S4 Bond Lengths for 2b.

Atom	Atom	Length/Å	Atom	Atom	Length/Å
O32	C1	1.4248(13)	C2	C3	1.5324(16)

Table S4 Bond Lengths for 2b.

Atom	Atom	Length/Å	Atom	Atom	Length/Å
O15	C14	1.2257(14)	C2	C1	1.5347(15)
N36	C35	1.1430(17)	C26	C27	1.394(2)
C6	C5	1.5547(15)	C26	C31	1.391(2)
C6	C14	1.5321(15)	C4	C3	1.5640(15)
C6	C1	1.5627(15)	C10	C11	1.3913(18)
C25	C24	1.1992(18)	C10	C9	1.3871(18)
C25	C26	1.4398(17)	C10	C13	1.5064(18)
C37	C38	1.3948(17)	C41	C42	1.3790(18)
C37	C14	1.4826(16)	C3	C16	1.4622(16)
C37	C42	1.4022(17)	C1	C7	1.5299(16)
N34	C33	1.1444(17)	C12	C7	1.3907(17)
C33	C4	1.4813(16)	C12	C11	1.3904(18)
C40	C41	1.3925(19)	C30	C29	1.377(3)
C40	C39	1.3917(19)	C30	C31	1.387(2)
C40	C43	1.5079(19)	C18	C17	1.4364(17)
C5	C24	1.4676(16)	C18	C23	1.3931(19)
C5	C4	1.5700(15)	C18	C19	1.3917(19)
C35	C4	1.4829(16)	C17	C16	1.1962(18)
C38	C39	1.3898(18)	C23	C22	1.381(2)
C8	C7	1.3916(17)	C19	C20	1.386(2)
C8	C9	1.3882(18)	C22	C21	1.377(3)
C28	C27	1.3871(19)	C20	C21	1.379(3)
C28	C29	1.380(3)			

Table S5 Bond Angles for 2b.

Atom	Atom	Atom	Angle/°	Atom	Atom	Atom	Angle/°
C5	C6	C1	109.85(9)	O15	C14	C37	120.72(10)

Table S5 Bond Angles for 2b.

Atom	Atom	Atom	Angle/°
C14	C6	C5	107.83(9)
C14	C6	C1	110.69(9)
C24	C25	C26	179.41(16)
C38	C37	C14	124.04(11)
C38	C37	C42	118.46(11)
C42	C37	C14	117.51(10)
N34	C33	C4	178.34(13)
C41	C40	C43	119.98(13)
C39	C40	C41	118.26(12)
C39	C40	C43	121.76(13)
C6	C5	C4	110.84(9)
C24	C5	C6	112.29(10)
C24	C5	C4	110.29(9)
N36	C35	C4	178.44(13)
C39	C38	C37	120.40(11)
C9	C8	C7	120.93(11)
C29	C28	C27	120.32(15)
C25	C24	C5	177.71(13)
C3	C2	C1	111.10(9)
C27	C26	C25	120.62(12)
C31	C26	C25	120.13(13)
C31	C26	C27	119.25(12)
C33	C4	C5	109.29(9)
C33	C4	C35	107.03(9)
C33	C4	C3	108.10(9)
C35	C4	C5	110.40(9)
C35	C4	C3	111.32(9)
C37	C14	C6	121.83(10)
C2	C3	C4	110.32(9)
C16	C3	C2	112.73(9)
C16	C3	C4	110.82(9)
O32	C1	C6	110.78(9)
O32	C1	C2	105.33(9)
O32	C1	C7	111.02(9)
C2	C1	C6	107.36(9)
C7	C1	C6	111.91(9)
C7	C1	C2	110.16(9)
C11	C12	C7	120.70(11)
C28	C27	C26	120.12(14)
C41	C42	C37	120.59(12)
C29	C30	C31	120.74(17)
C23	C18	C17	120.81(12)
C19	C18	C17	119.81(12)
C19	C18	C23	119.35(12)
C8	C7	C1	120.63(10)
C12	C7	C8	117.93(11)
C12	C7	C1	121.40(10)
C12	C11	C10	121.55(11)
C10	C9	C8	121.50(11)
C16	C17	C18	177.23(13)
C38	C39	C40	121.08(12)
C30	C29	C28	119.74(14)
C17	C16	C3	178.34(13)
C22	C23	C18	120.05(14)

Table S5 Bond Angles for 2b.

Atom	Atom	Atom	Angle/°	Atom	Atom	Atom	Angle/°
C3	C4	C5	110.59(9)	C20	C19	C18	119.83(14)
C11	C10	C13	121.89(11)	C21	C22	C23	120.46(15)
C9	C10	C11	117.37(11)	C21	C20	C19	120.43(15)
C9	C10	C13	120.74(11)	C22	C21	C20	119.87(14)
C42	C41	C40	121.21(12)	C30	C31	C26	119.83(16)
O15	C14	C6	117.45(10)				

Table S6 Hydrogen Atom Coordinates (Å×10^4) and Isotropic Displacement Parameters (Å^2×10^3) for 2b.

Atom	*x*	*y*	*z*	U(eq)
H6	6533	5943	391	22
H5	6724	6724	1295	23
H38	6985	5920	-339	30
H8	4890	7002	455	29
H28	9902	6882	1127	50
H2A	5280	5655	1224	23
H2B	5818	5361	918	23
H41	7289	7689	-1263	35
H3	5949	6109	1839	24
H12	5630	5468	-31	29
H27	8909	6841	1376	38
H42	6822	7701	-433	30
H30	9511	6126	-298	69
H11	4963	5422	-741	32
H9	4240	6962	-267	32
H13A	4237	5911	-1330	53
H13B	4006	6549	-1179	53
H13C	3713	5986	-913	53

Table S6 Hydrogen Atom Coordinates (Å×10[4]) and Isotropic Displacement Parameters (Å[2]×10[3]) for 2b.

Atom	*x*	*y*	*z*	U(eq)
H39	7450	5915	-1176	36
H29	10203	6518	293	62
H23	5173	3929	2293	42
H19	6415	4664	3261	42
H43A	7495	7060	-2104	65
H43B	7723	6409	-2007	65
H43C	8114	6952	-1827	65
H22	5006	3202	2938	55
H20	6230	3937	3906	56
H21	5527	3209	3746	55
H31	8513	6093	-64	54
H32	5756(8)	7079(8)	994(7)	41(5)

Crystal structure determination of [2b]

Crystal Data for $C_{39}H_{30}N_2O_2$ (M =558.65 g/mol): tetragonal, space group $I4_1/a$ (no. 88), a = 22.45227(12) Å, c = 24.6012(2) Å, V = 12401.59(17) Å^3, Z = 16, T = 101.7(7) K, μ(CuKα) = 0.578 mm^{-1}, $Dcalc$ = 1.197 g/cm^3, 25291 reflections measured (5.328° ≤ 2Θ ≤ 140.738°), 5899 unique (R_{int} = 0.0442, R_{sigma} = 0.0300) which were used in all calculations. The final R_1 was 0.0399 (I > 2σ(I)) and wR_2 was 0.1078 (all data).

Refinement model description

Number of restraints - 0, number of constraints - unknown.

Details:

1. Fixed Uiso
 At 1.2 times of:
  All C(H) groups, All C(H,H) groups
 At 1.5 times of:
  All C(H,H,H) groups
2.a Ternary CH refined with riding coordinates:
 C6(H6), C5(H5), C3(H3)
2.b Secondary CH2 refined with riding coordinates:
 C2(H2A,H2B)
2.c Aromatic/amide H refined with riding coordinates:
 C38(H38), C8(H8), C28(H28), C41(H41), C12(H12), C27(H27), C42(H42), C30(H30), C11(H11), C9(H9), C39(H39), C29(H29), C23(H23), C19(H19), C22(H22), C20(H20), C21(H21), C31(H31)

2.d Idealised Me refined as rotating group:
C13(H13A,H13B,H13C), C43(H43A,H43B,H43C)

2e

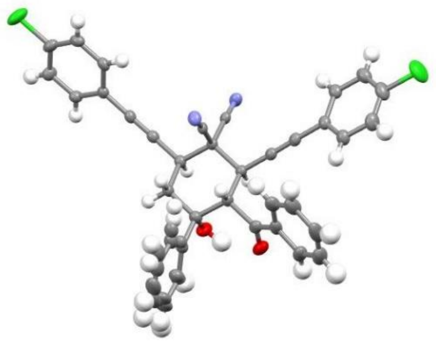

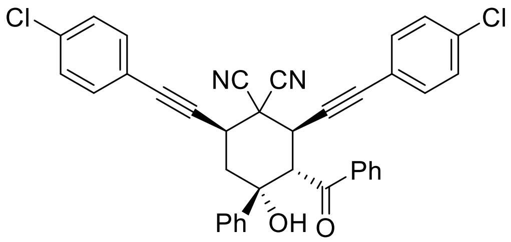

CCDC 1998289

Table S7 Crystal data and structure refinement for 2e.

Identification code	2e
Empirical formula	$C_{37}H_{24}Cl_2N_2O_2$
Formula weight	599.48
Temperature/K	100(2)
Crystal system	orthorhombic
Space group	$Pna2_1$
a/Å	6.71239(6)
b/Å	21.39848(16)
c/Å	21.00387(14)
α/°	90
β/°	90
γ/°	90
Volume/Å^3	3016.89(4)
Z	4
ρ_{calc}g/cm^3	1.320
μ/mm^{-1}	2.224
F(000)	1240.0
Crystal size/mm^3	0.35 × 0.32 × 0.25
Radiation	CuKα (λ = 1.54184)

2Θ range for data collection/°	5.896 to 141.022
Index ranges	$-7 \leq h \leq 8$, $-26 \leq k \leq 26$, $-25 \leq l \leq 25$
Reflections collected	62968
Independent reflections	5768 [R_{int} = 0.0366, R_{sigma} = 0.0161]
Data/restraints/parameters	5768/1/384
Goodness-of-fit on F^2	1.037
Final R indexes [I>=2σ (I)]	R_1 = 0.0295, wR_2 = 0.0710
Final R indexes [all data]	R_1 = 0.0301, wR_2 = 0.0717
Largest diff. peak/hole / e $Å^{-3}$	0.13/-0.36
Flack parameter	0.000(3)

Table S8 Fractional Atomic Coordinates (×10^4) and Equivalent Isotropic Displacement Parameters ($Å^2$×10^3) for 2e. U_{eq} is defined as 1/3 of of the trace of the orthogonalised U_{IJ} tensor.

Atom	*x*	*y*	*z*	U(eq)
Cl32	12278.5(11)	3075.4(4)	1592.6(4)	58.4(2)
Cl23	1049.0(16)	1378.5(4)	8405.3(4)	69.3(3)
O14	-1802(3)	4934.3(9)	5623.1(10)	43.9(5)
O33	-471(3)	4913.6(9)	4429.1(9)	43.1(5)
N37	1660(3)	2515.8(9)	4321.6(10)	33.0(4)
N35	6176(3)	3734.1(10)	5200.4(11)	33.9(5)
C34	4660(3)	3668.2(10)	4966.5(10)	23.8(4)
C6	2655(3)	3646.7(10)	4676.4(10)	22.6(4)
C2	2952(4)	4757.8(10)	4271.2(11)	27.0(5)
C26	7067(3)	3569.8(9)	2842.9(10)	24.7(4)
C17	1166(3)	2682.8(11)	6716.5(11)	28.6(5)
C25	5438(4)	3737.7(10)	3251.1(10)	26.1(5)
C1	2623(3)	4075.1(10)	4071.7(10)	24.2(4)

Table S8 Fractional Atomic Coordinates (×10^4) and Equivalent Isotropic Displacement Parameters (Å^2×10^3) for 2e. U_{eq} is defined as 1/3 of of the trace of the orthogonalised U_{IJ} tensor.

Atom	*x*	*y*	*z*	U(eq)
C36	2164(3)	2998.8(10)	4484.7(10)	24.7(5)
C24	4125(4)	3877.9(10)	3609.0(11)	26.2(5)
C31	8712(4)	3256.0(10)	3093.6(11)	27.7(5)
C30	10313(4)	3101.7(11)	2712.8(12)	31.1(5)
C18	1022(4)	2918.4(12)	7334.0(12)	34.7(5)
C28	8645(4)	3576.4(12)	1810.8(12)	33.7(5)
C16	1159(3)	3101.3(11)	6180.1(11)	28.3(5)
C22	1292(3)	2037.1(11)	6627.4(12)	31.1(5)
C4	1426(4)	4563.2(10)	5353.1(10)	26.5(5)
C21	1256(4)	1633.9(12)	7144.4(13)	38.1(6)
C27	7050(4)	3724.5(10)	2195.4(11)	28.6(5)
C13	-159(4)	4779.6(10)	5828.6(12)	33.8(5)
C3	1391(4)	4983.0(11)	4746.4(11)	32.2(5)
C5	1066(3)	3866.7(10)	5177.9(11)	23.9(4)
C29	10266(4)	3264.5(12)	2075.3(12)	33.8(5)
C15	1103(3)	3450.1(10)	5734.5(11)	25.4(4)
C38	272(5)	4810.5(11)	6518.7(12)	38.9(6)
C7B	1626(6)	5664.3(11)	4872(2)	29.1(11)
C8B	229(5)	6106.3(15)	4683(2)	44.3(13)
C9B	582(6)	6737.7(13)	4790(2)	57.2(16)
C10B	2331(7)	6926.9(11)	5087(2)	56.3(16)
C11B	3728(6)	6484.8(15)	5276.6(18)	47.8(13)
C12B	3375(6)	5853.5(14)	5169(2)	36.6(12)
C19	977(4)	2518.1(14)	7851.8(12)	42.0(6)
C39	2062(5)	4628.8(12)	6786.2(12)	43.2(7)
C43	-1249(5)	5044.1(13)	6915.2(15)	50.1(8)

Table S8 Fractional Atomic Coordinates (×10^4) and Equivalent Isotropic Displacement Parameters (Å^2×10^3) for 2e. U_{eq} is defined as 1/3 of of the trace of the orthogonalised U_{IJ} tensor.

Atom	*x*	*y*	*z*	U(eq)
C42	-957(7)	5088.4(15)	7559.5(16)	59.9(10)
C40	2351(6)	4672.5(13)	7442.6(13)	52.4(8)
C41	825(7)	4901.5(14)	7823.9(14)	61.9(11)
C20	1088(4)	1882.2(14)	7750.4(13)	44.3(7)
C7A	2222(11)	5659(2)	4998(4)	29.1(11)
C12A	4065(10)	5739(2)	5288(4)	36.6(12)
C11A	4691(9)	6334(3)	5465(3)	47.8(13)
C10A	3474(10)	6847(2)	5352(3)	56.3(16)
C9A	1630(10)	6767(2)	5062(4)	57.2(16)
C8A	1004(9)	6172(3)	4885(4)	44.3(13)

Table S9 Anisotropic Displacement Parameters (Å^2×10^3) for 2e. The Anisotropic displacement factor exponent takes the form: $-2\pi^2[h^2a^{*2}U_{11}+2hka^*b^*U_{12}+\ldots]$.

Atom	U_{11}	U_{22}	U_{33}	U_{23}	U_{13}	U_{12}
Cl32	44.4(4)	82.9(5)	47.8(4)	-20.3(4)	9.2(3)	21.6(4)
Cl23	107.8(7)	62.7(5)	37.4(4)	22.6(3)	-13.7(4)	-4.4(5)
O14	38.1(11)	42.2(10)	51.5(11)	-9.2(9)	12.4(9)	3.6(8)
O33	37.3(10)	52.7(11)	39.3(10)	7.4(9)	2.9(8)	16.8(9)
N37	35.3(11)	27.4(9)	36.2(10)	-6.8(9)	9.3(9)	-5.0(8)
N35	29.2(11)	37.7(11)	34.8(11)	4.1(9)	-2.4(9)	-2.9(9)
C34	26.0(12)	22.9(10)	22.4(10)	2.7(8)	4.6(9)	-1.4(9)
C6	24.2(10)	20.8(10)	22.6(10)	-1.6(8)	2.3(9)	-1.8(8)
C2	34.3(13)	22.9(10)	23.9(10)	1.6(8)	3.4(9)	0.8(9)
C26	28.3(11)	21.5(10)	24.5(11)	-1.4(8)	1.2(9)	-2.3(9)
C17	26.2(11)	33.7(12)	26.1(11)	2.8(9)	-0.6(9)	-2.0(9)
C25	32.0(12)	24.7(11)	21.5(11)	1.7(8)	-0.2(9)	-2.4(9)

Table S9 Anisotropic Displacement Parameters ($Å^2 \times 10^3$) for 2e. The Anisotropic displacement factor exponent takes the form: $-2\pi^2[h^2a^{*2}U_{11}+2hka^*b^*U_{12}+…]$.

Atom	U_{11}	U_{22}	U_{33}	U_{23}	U_{13}	U_{12}
C1	26.1(11)	24.1(10)	22.4(10)	-0.4(8)	-1.3(9)	-1.4(8)
C36	24.1(11)	26.4(11)	23.5(10)	-0.2(9)	5.1(9)	0.0(9)
C24	31.1(12)	23.9(10)	23.6(10)	0.7(8)	-2.2(9)	-0.4(9)
C31	33.4(12)	22.9(10)	26.8(11)	3.5(9)	-3.4(10)	-3.7(9)
C30	30.4(12)	24.5(11)	38.4(13)	-0.6(9)	-4.1(10)	1.9(9)
C18	37.7(14)	36.1(13)	30.2(12)	0.1(10)	-0.4(10)	-5.8(11)
C28	38.8(14)	39.2(13)	23.1(11)	-4.4(10)	0.3(10)	4.8(11)
C16	26.5(12)	31.4(12)	27.1(11)	-2.8(9)	3.4(9)	-2.0(9)
C22	28.5(11)	35.2(12)	29.6(11)	2.2(10)	-3.1(10)	-1.6(9)
C4	33.5(12)	22.8(10)	23.2(11)	-3.0(8)	4.4(9)	-0.2(9)
C21	39.5(14)	34.1(12)	40.9(14)	5.4(11)	-6.7(12)	-2.0(11)
C27	32.2(12)	28.4(11)	25.1(11)	-0.3(9)	-1.5(10)	5.5(9)
C13	38.9(14)	23.5(11)	39.0(13)	-5.4(10)	10.8(11)	-3.2(10)
C3	39.9(14)	25.7(11)	31.0(12)	0.1(9)	4.4(10)	3.0(10)
C5	23.5(10)	24.2(10)	24.1(10)	-1.2(9)	2.6(9)	0.8(8)
C29	30.3(13)	35.7(12)	35.4(12)	-9.7(10)	4.4(11)	5.3(10)
C15	24.3(11)	26.3(10)	25.7(11)	-3.5(9)	2.5(9)	-2.1(9)
C38	61.1(18)	22.8(11)	32.7(13)	-5.0(10)	17.6(13)	-3.6(11)
C7B	38(3)	23.9(12)	25(2)	-0.9(12)	4(2)	0.3(15)
C8B	57(4)	28.6(17)	48(3)	1.7(19)	11(2)	6(2)
C9B	80(5)	25.1(16)	66(4)	1(2)	23(3)	12(3)
C10B	91(5)	28(2)	50(3)	-8(2)	23(3)	-10(3)
C11B	66(4)	39(3)	38(3)	-8.2(19)	8(2)	-14(2)
C12B	48(4)	29.5(19)	33(2)	-5.5(17)	5(2)	-6(2)
C19	49.1(16)	52.7(15)	24.1(11)	0.4(12)	-1.7(11)	-5.5(13)
C39	69.7(19)	29.3(12)	30.8(13)	-6.6(10)	12.5(13)	1.6(13)
C43	69(2)	33.7(14)	47.6(16)	-7.4(12)	28.1(16)	1.1(13)

Table S9 Anisotropic Displacement Parameters (Å^2 ×10^3) for 2e. The Anisotropic displacement factor exponent takes the form: $-2\pi^2[h^2a^{*2}U_{11}+2hka^*b^*U_{12}+…]$.

Atom	U_{11}	U_{22}	U_{33}	U_{23}	U_{13}	U_{12}
C42	92(3)	44.5(17)	43.4(17)	-7.8(14)	32.4(18)	0.3(17)
C40	90(2)	35.6(14)	31.4(13)	-6.5(11)	6.8(15)	0.7(15)
C41	120(3)	36.7(15)	28.6(14)	-7.5(12)	25.0(18)	-8.8(18)
C20	49.6(16)	49.7(16)	33.6(14)	13.5(12)	-7.7(12)	-5.2(13)
C7A	38(3)	23.9(12)	25(2)	-0.9(12)	4(2)	0.3(15)
C12A	48(4)	29.5(19)	33(2)	-5.5(17)	5(2)	-6(2)
C11A	66(4)	39(3)	38(3)	-8.2(19)	8(2)	-14(2)
C10A	91(5)	28(2)	50(3)	-8(2)	23(3)	-10(3)
C9A	80(5)	25.1(16)	66(4)	1(2)	23(3)	12(3)
C8A	57(4)	28.6(17)	48(3)	1.7(19)	11(2)	6(2)

Table S10 Bond Lengths for 2e.

Atom	Atom	Length/Å	Atom	Atom	Length/Å
Cl32	C29	1.737(2)	C4	C13	1.531(3)
Cl23	C20	1.748(3)	C4	C3	1.559(3)
O14	C13	1.230(3)	C4	C5	1.554(3)
O33	C3	1.424(3)	C21	C20	1.384(4)
N37	C36	1.140(3)	C13	C38	1.480(4)
N35	C34	1.139(3)	C3	C7B	1.490(3)
C34	C6	1.478(3)	C3	C7A	1.637(5)
C6	C1	1.566(3)	C5	C15	1.470(3)
C6	C36	1.481(3)	C38	C39	1.382(4)
C6	C5	1.572(3)	C38	C43	1.409(4)
C2	C1	1.536(3)	C7B	C8B	1.3900
C2	C3	1.525(3)	C7B	C12B	1.3900
C26	C25	1.435(3)	C8B	C9B	1.3900
C26	C31	1.395(3)	C9B	C10B	1.3900

Table S10 Bond Lengths for 2e.

Atom	Atom	Length/Å	Atom	Atom	Length/Å
C26	C27	1.400(3)	C10B	C11B	1.3900
C17	C18	1.395(3)	C11B	C12B	1.3900
C17	C16	1.439(3)	C19	C20	1.379(4)
C17	C22	1.397(3)	C39	C40	1.395(4)
C25	C24	1.197(3)	C43	C42	1.371(5)
C1	C24	1.462(3)	C42	C41	1.378(5)
C31	C30	1.380(3)	C40	C41	1.389(5)
C30	C29	1.384(4)	C7A	C12A	1.3900
C18	C19	1.385(4)	C7A	C8A	1.3900
C28	C27	1.378(3)	C12A	C11A	1.3900
C28	C29	1.392(4)	C11A	C10A	1.3900
C16	C15	1.198(3)	C10A	C9A	1.3900
C22	C21	1.387(4)	C9A	C8A	1.3900

Table S11 Bond Angles for 2e.

Atom	Atom	Atom	Angle/°	Atom	Atom	Atom	Angle/°
N35	C34	C6	174.5(2)	C2	C3	C7A	104.9(4)
C34	C6	C1	109.19(17)	C4	C3	C7A	103.9(3)
C34	C6	C36	110.11(18)	C7B	C3	C2	110.6(3)
C34	C6	C5	109.42(17)	C7B	C3	C4	114.7(3)
C1	C6	C5	111.02(17)	C4	C5	C6	109.89(17)
C36	C6	C1	108.94(17)	C15	C5	C6	109.86(18)
C36	C6	C5	108.15(17)	C15	C5	C4	112.99(18)
C3	C2	C1	112.34(19)	C30	C29	Cl32	119.22(19)
C31	C26	C25	119.9(2)	C30	C29	C28	121.6(2)
C31	C26	C27	119.1(2)	C28	C29	Cl32	119.1(2)
C27	C26	C25	121.0(2)	C16	C15	C5	178.5(2)

Table S11 Bond Angles for 2e.

Atom	Atom	Atom	Angle/°	Atom	Atom	Atom	Angle/°
C18	C17	C16	120.2(2)	C39	C38	C13	123.8(2)
C18	C17	C22	119.1(2)	C39	C38	C43	119.3(3)
C22	C17	C16	120.7(2)	C43	C38	C13	116.9(3)
C24	C25	C26	177.7(2)	C8B	C7B	C3	123.0(2)
C2	C1	C6	109.49(17)	C8B	C7B	C12B	120.0
C24	C1	C6	111.12(18)	C12B	C7B	C3	117.0(2)
C24	C1	C2	110.89(18)	C7B	C8B	C9B	120.0
N37	C36	C6	175.1(2)	C8B	C9B	C10B	120.0
C25	C24	C1	175.9(2)	C11B	C10B	C9B	120.0
C30	C31	C26	120.8(2)	C10B	C11B	C12B	120.0
C31	C30	C29	118.9(2)	C11B	C12B	C7B	120.0
C19	C18	C17	120.5(2)	C20	C19	C18	119.2(2)
C27	C28	C29	118.9(2)	C38	C39	C40	120.2(3)
C15	C16	C17	178.4(3)	C42	C43	C38	120.3(4)
C21	C22	C17	120.6(2)	C43	C42	C41	120.1(3)
C13	C4	C3	110.38(19)	C41	C40	C39	119.4(4)
C13	C4	C5	109.64(18)	C42	C41	C40	120.7(3)
C5	C4	C3	110.90(18)	C21	C20	Cl23	119.2(2)
C20	C21	C22	118.9(2)	C19	C20	Cl23	119.1(2)
C28	C27	C26	120.6(2)	C19	C20	C21	121.7(2)
O14	C13	C4	118.4(2)	C12A	C7A	C3	123.6(4)
O14	C13	C38	120.5(2)	C12A	C7A	C8A	120.0
C38	C13	C4	121.1(2)	C8A	C7A	C3	116.3(3)
O33	C3	C2	105.29(19)	C7A	C12A	C11A	120.0
O33	C3	C4	109.6(2)	C10A	C11A	C12A	120.0
O33	C3	C7B	106.1(2)	C11A	C10A	C9A	120.0
O33	C3	C7A	122.8(3)	C10A	C9A	C8A	120.0

Table S11 Bond Angles for 2e.

Atom	Atom	Atom	Angle/°	Atom	Atom	Atom	Angle/°
C2	C3	C4	110.02(19)	C9A	C8A	C7A	120.0

Table S12 Hydrogen Atom Coordinates (Å×10[4]) and Isotropic Displacement Parameters (Å[2]×10[3]) for 2e.

Atom	*x*	*y*	*z*	U(eq)
H33	-1403	4972	4689	65
H2A	4292	4799	4464	32
H2B	2909	5027	3888	32
H1	1280	4041	3868	29
H31	8730	3147	3532	33
H30	11429	2887	2885	37
H18	954	3357	7400	42
H28	8638	3685	1372	40
H22	1404	1873	6209	37
H4	2765	4601	5558	32
H21	1345	1195	7083	46
H27	5929	3933	2020	34
H5	-280	3834	4977	29
H8B	-966	5977	4480	53
H9B	-372	7040	4661	69
H10B	2573	7358	5161	68
H11B	4924	6614	5480	57
H12B	4330	5551	5299	44
H19	871	2679	8272	50
H39	3100	4474	6522	52
H43	-2483	5171	6735	60
H42	-1985	5248	7824	72
H40	3579	4547	7627	63

Table S12 Hydrogen Atom Coordinates (Å×10^4) and Isotropic Displacement Parameters ($Å^2$×10^3) for 2e.

Atom	*x*	*y*	*z*	U(eq)
H41	1012	4930	8271	74
H12A	4897	5388	5365	44
H11A	5951	6389	5663	57
H10A	3901	7253	5473	68
H9A	798	7118	4985	69
H8A	-256	6117	4687	53

Table S13 Atomic Occupancy for 2e.

Atom	*Occupancy*	Atom	*Occupancy*	Atom	*Occupancy*
C7B	0.607(4)	C8B	0.607(4)	H8B	0.607(4)
C9B	0.607(4)	H9B	0.607(4)	C10B	0.607(4)
H10B	0.607(4)	C11B	0.607(4)	H11B	0.607(4)
C12B	0.607(4)	H12B	0.607(4)	C7A	0.393(4)
C12A	0.393(4)	H12A	0.393(4)	C11A	0.393(4)
H11A	0.393(4)	C10A	0.393(4)	H10A	0.393(4)
C9A	0.393(4)	H9A	0.393(4)	C8A	0.393(4)
H8A	0.393(4)				

Crystal structure determination of [2e]

Crystal Data for $C_{37}H_{24}Cl_2N_2O_2$ (M =599.48 g/mol): orthorhombic, space group Pna2_1 (no. 33), a = 6.71239(6) Å, b = 21.39848(16) Å, c = 21.00387(14) Å, V = 3016.89(4) $Å^3$, Z = 4, T = 100(2) K, μ(CuKα) = 2.224 mm^{-1}, $Dcalc$ = 1.320 g/cm^3, 62968 reflections measured (5.896° ≤ 2Θ ≤ 141.022°), 5768 unique (R_{int} = 0.0366, R_{sigma} = 0.0161) which were used in all calculations. The final R_1 was 0.0295 (I > 2σ(I)) and wR_2 was 0.0717 (all data).

Refinement model description

Number of restraints - 1, number of constraints - unknown.

Details:

1. Fixed Uiso
 At 1.2 times of:
  All C(H) groups, All C(H,H) groups
 At 1.5 times of:

All O(H) groups
2. Uiso/Uaniso restraints and constraints
Uanis(C7B) = Uanis(C7A)
Uanis(C8B) = Uanis(C8A)
Uanis(C9B) = Uanis(C9A)
Uanis(C10B) = Uanis(C10A)
Uanis(C11B) = Uanis(C11A)
Uanis(C12B) = Uanis(C12A)
3. Others
Sof(C7B)=Sof(C8B)=Sof(H8B)=Sof(C9B)=Sof(H9B)=Sof(C10B)=Sof(H10B)=Sof(C11B)=
Sof(H11B)=Sof(C12B)=Sof(H12B)=1-FVAR(1)

Sof(C7A)=Sof(C12A)=Sof(H12A)=Sof(C11A)=Sof(H11A)=Sof(C10A)=Sof(H10A)=Sof(C9A)
=
Sof(H9A)=Sof(C8A)=Sof(H8A)=FVAR(1)
4.a Ternary CH refined with riding coordinates:
C1(H1), C4(H4), C5(H5)
4.b Secondary CH2 refined with riding coordinates:
C2(H2A,H2B)
4.c Aromatic/amide H refined with riding coordinates:
C31(H31), C30(H30), C18(H18), C28(H28), C22(H22), C21(H21), C27(H27),
C8B(H8B), C9B(H9B), C10B(H10B), C11B(H11B), C12B(H12B), C19(H19), C39(H39),
C43(H43), C42(H42), C40(H40), C41(H41), C12A(H12A), C11A(H11A), C10A(H10A),
C9A(H9A), C8A(H8A)
4.d Fitted hexagon refined as free rotating group:
C7B(C8B,C9B,C10B,C11B,C12B), C7A(C12A,C11A,C10A,C9A,C8A)
4.e Idealised tetrahedral OH refined as rotating group:
O33(H33)

3a

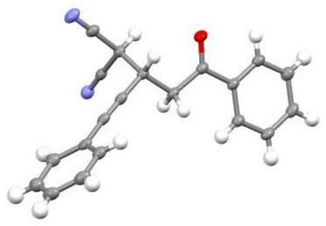

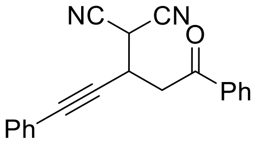

CCDC 1998291

Table S14 Crystal data and structure refinement for 3a.

Identification code	3a
Empirical formula	$C_{20}H_{14}N_2O$
Formula weight	298.33
Temperature/K	101.8(6)
Crystal system	triclinic
Space group	P-1
a/Å	5.4623(2)
b/Å	10.3098(3)
c/Å	14.3344(5)
α/°	96.478(3)
β/°	100.889(3)
γ/°	98.731(3)
Volume/Å^3	775.15(5)
Z	2
ρ_{calc}g/cm^3	1.278
μ/mm^{-1}	0.634
F(000)	312.0
Crystal size/mm^3	? × ? × ?
Radiation	CuKα (λ = 1.54184)
2Θ range for data collection/°	6.346 to 140.99

Index ranges	-6 ≤ h ≤ 6, -10 ≤ k ≤ 12, -17 ≤ l ≤ 17
Reflections collected	8381
Independent reflections	2951 [R_{int} = 0.0452, R_{sigma} = 0.0380]
Data/restraints/parameters	2951/0/209
Goodness-of-fit on F^2	1.081
Final R indexes [I>=2σ (I)]	R_1 = 0.0459, wR_2 = 0.1314
Final R indexes [all data]	R_1 = 0.0489, wR_2 = 0.1351
Largest diff. peak/hole / e Å^{-3}	0.22/-0.23

Table S15 Fractional Atomic Coordinates (×10^4) and Equivalent Isotropic Displacement Parameters (Å^2×10^3) for 3a. U_{eq} is defined as 1/3 of of the trace of the orthogonalised U_{IJ} tensor.

Atom	*x*	*y*	*z*	U(eq)
O17	1427.7(16)	2175.0(8)	9813.9(6)	28.7(3)
C9	-2725(2)	1592.5(11)	8296.6(9)	23.0(3)
N14	-7306(2)	-642.1(10)	8997.9(8)	30.1(3)
C2	-1852(2)	4470.8(12)	10995.6(9)	26.4(3)
C8	-2711(2)	2572.0(11)	9189.6(9)	23.5(3)
C19	-10534(3)	1347.9(13)	5742.8(10)	30.3(3)
C1	-42(2)	3686.3(11)	10851.8(9)	22.5(3)
C4	605(3)	5380.3(12)	12573.8(9)	28.4(3)
C18	-8101(2)	2102.0(12)	6015.8(9)	25.5(3)
C6	2085(2)	3762.9(12)	11577.7(9)	25.1(3)
C7	-294(2)	2756.8(11)	9945.3(9)	23.2(3)
C10	-2996(2)	128.4(11)	8508.6(9)	23.2(3)
C23	-7416(3)	3139.8(12)	5511.8(9)	29.1(3)
C20	-12250(3)	1641.9(14)	4986.8(11)	36.0(3)
C13	-5426(2)	-314.2(11)	8777.5(8)	23.9(3)

Table S15 Fractional Atomic Coordinates (×10^4) and Equivalent Isotropic Displacement Parameters (Å^2×10^3) for 3a. U_{eq} is defined as 1/3 of of the trace of the orthogonalised U_{IJ} tensor.

Atom	*x*	*y*	*z*	U(eq)
C16	-6297(2)	1852.0(11)	6817.0(9)	25.6(3)
C5	2400(3)	4597.4(13)	12437.6(9)	29.2(3)
N12	-2639(2)	-1411.3(11)	6975.6(9)	36.6(3)
C3	-1506(2)	5319.5(12)	11855.2(10)	28.8(3)
C21	-11570(3)	2677.7(14)	4497.0(10)	35.9(3)
C22	-9157(3)	3420.6(13)	4760.3(10)	33.8(3)
C11	-2789(2)	-754.1(12)	7654.6(9)	26.5(3)
C15	-4704(2)	1722.3(11)	7484.8(9)	25.2(3)

Table S16 Anisotropic Displacement Parameters (Å^2×10^3) for 3a. The Anisotropic displacement factor exponent takes the form: $-2\pi^2[h^2a^{*2}U_{11}+2hka^*b^*U_{12}+\ldots]$.

Atom	U_{11}	U_{22}	U_{33}	U_{23}	U_{13}	U_{12}
O17	27.1(5)	27.2(5)	32.9(5)	-4.0(4)	10.5(4)	8.8(4)
C9	26.1(6)	18.8(6)	25.6(6)	0.8(5)	10.1(5)	4.7(4)
N14	31.4(6)	24.4(5)	34.8(6)	2.6(5)	9.5(5)	4.3(4)
C2	27.7(6)	23.1(6)	29.8(7)	0.1(5)	10.0(5)	6.2(5)
C8	28.1(6)	17.8(6)	26.2(6)	-0.2(5)	9.9(5)	5.9(5)
C19	36.6(7)	26.4(6)	30.8(7)	2.7(5)	12.8(5)	8.4(5)
C1	26.3(6)	16.9(5)	26.5(6)	1.9(5)	11.5(5)	3.7(4)
C4	38.4(7)	21.4(6)	26.7(6)	-0.9(5)	13.9(5)	3.6(5)
C18	34.6(7)	22.0(6)	23.0(6)	-1.6(5)	10.9(5)	12.0(5)
C6	29.6(6)	20.1(6)	28.4(7)	3.0(5)	10.6(5)	7.4(5)
C7	26.0(6)	17.3(5)	28.3(7)	2.0(5)	11.8(5)	3.8(4)
C10	26.5(6)	20.2(6)	23.1(6)	-0.6(5)	6.8(5)	5.7(5)
C23	37.0(7)	24.8(6)	27.8(7)	0.7(5)	11.8(5)	9.0(5)
C20	34.1(7)	36.4(7)	36.7(8)	-0.5(6)	6.4(6)	9.8(6)

Table S16 Anisotropic Displacement Parameters (Å^2 ×10^3) for 3a. The Anisotropic displacement factor exponent takes the form: $-2\pi^2[h^2a^{*2}U_{11}+2hka^*b^*U_{12}+\ldots]$.

Atom	U_{11}	U_{22}	U_{33}	U_{23}	U_{13}	U_{12}
C13	30.2(6)	17.4(5)	23.9(6)	0.1(5)	5.2(5)	6.3(5)
C16	34.2(7)	19.1(6)	25.6(6)	-0.3(5)	11.6(5)	7.6(5)
C5	34.4(7)	26.6(6)	26.8(7)	1.0(5)	7.5(5)	6.6(5)
N12	50.7(7)	28.5(6)	32.5(6)	-2.2(5)	12.0(5)	13.9(5)
C3	32.9(7)	22.3(6)	34.6(7)	-0.1(5)	15.2(5)	8.3(5)
C21	44.6(8)	37.7(8)	28.5(7)	3.0(6)	6.4(6)	20.2(6)
C22	49.1(8)	30.1(7)	28.3(7)	7.5(5)	14.5(6)	15.9(6)
C11	31.6(6)	20.8(6)	29.3(7)	2.7(5)	9.0(5)	8.9(5)
C15	32.6(7)	18.1(6)	26.6(6)	0.3(5)	11.4(5)	5.7(5)

Table S17 Bond Lengths for 3a.

Atom	Atom	Length/Å	Atom	Atom	Length/Å
O17	C7	1.2218(15)	C4	C5	1.3888(18)
C9	C8	1.5364(15)	C4	C3	1.3816(19)
C9	C10	1.5639(16)	C18	C23	1.4005(18)
C9	C15	1.4643(17)	C18	C16	1.4397(17)
N14	C13	1.1446(17)	C6	C5	1.3873(17)
C2	C1	1.3988(17)	C10	C13	1.4726(17)
C2	C3	1.3922(18)	C10	C11	1.4735(16)
C8	C7	1.5128(17)	C23	C22	1.3841(19)
C19	C18	1.3947(19)	C20	C21	1.385(2)
C19	C20	1.386(2)	C16	C15	1.2004(18)
C1	C6	1.3915(17)	N12	C11	1.1450(17)
C1	C7	1.4957(16)	C21	C22	1.381(2)

Table S18 Bond Angles for 3a.

Atom	Atom	Atom	Angle/°	Atom	Atom	Atom	Angle/°

Table S18 Bond Angles for 3a.

Atom	Atom	Atom	Angle/°	Atom	Atom	Atom	Angle/°
C8	C9	C10	111.82(10)	O17	C7	C1	120.66(11)
C15	C9	C8	111.40(9)	C1	C7	C8	118.48(10)
C15	C9	C10	110.81(10)	C13	C10	C9	111.83(10)
C3	C2	C1	119.96(12)	C13	C10	C11	109.40(10)
C7	C8	C9	113.19(10)	C11	C10	C9	109.57(10)
C20	C19	C18	119.83(12)	C22	C23	C18	120.04(13)
C2	C1	C7	122.69(11)	C21	C20	C19	120.62(13)
C6	C1	C2	119.19(11)	N14	C13	C10	178.94(13)
C6	C1	C7	118.12(10)	C15	C16	C18	175.79(13)
C3	C4	C5	119.95(12)	C6	C5	C4	120.03(12)
C19	C18	C23	119.31(12)	C4	C3	C2	120.35(11)
C19	C18	C16	121.32(11)	C22	C21	C20	119.75(13)
C23	C18	C16	119.36(12)	C21	C22	C23	120.46(13)
C5	C6	C1	120.51(11)	N12	C11	C10	178.04(13)
O17	C7	C8	120.85(11)	C16	C15	C9	178.69(13)

Table S19 Hydrogen Atom Coordinates (Å×10^4) and Isotropic Displacement Parameters (Å^2×10^3) for 3a.

Atom	*x*	*y*	*z*	U(eq)
H9	-1089	1816	8113	28
H2	-3286	4426	10517	32
H8A	-2937	3425	8996	28
H8B	-4134	2254	9468	28
H19	-11004	650	6067	36
H4	823	5945	13148	34
H6	3306	3251	11486	30
H10	-1615	71	9041	28
H23	-5791	3640	5682	35

Table S19 Hydrogen Atom Coordinates (Å×10^4) and Isotropic Displacement Parameters (Å^2×10^3) for 3a.

Atom	*x*	*y*	*z*	U(eq)
H20	-13873	1139	4807	43
H5	3814	4632	12923	35
H3	-2701	5848	11946	35
H21	-12735	2872	3993	43
H22	-8698	4114	4430	41

Crystal structure determination of [3a]

Crystal Data for $C_{20}H_{14}N_2O$ (M =298.33 g/mol): triclinic, space group P-1 (no. 2), a = 5.4623(2) Å, b = 10.3098(3) Å, c = 14.3344(5) Å, α = 96.478(3)°, β = 100.889(3)°, γ = 98.731(3)°, V = 775.15(5) Å^3, Z = 2, T = 101.8(6) K, μ(CuKα) = 0.634 mm^{-1}, $Dcalc$ = 1.278 g/cm^3, 8381 reflections measured (6.346° ≤ 2Θ ≤ 140.99°), 2951 unique (R_{int} = 0.0452, R_{sigma} = 0.0380) which were used in all calculations. The final R_1 was 0.0459 (I > 2σ(I)) and wR_2 was 0.1351 (all data).

Refinement model description

Number of restraints - 0, number of constraints - unknown.

Details:

1. Fixed Uiso
 At 1.2 times of:
  All C(H) groups, All C(H,H) groups
2.a Ternary CH refined with riding coordinates:
 C9(H9), C10(H10)
2.b Secondary CH2 refined with riding coordinates:
 C8(H8A,H8B)
2.c Aromatic/amide H refined with riding coordinates:
 C2(H2), C19(H19), C4(H4), C6(H6), C23(H23), C20(H20), C5(H5), C3(H3),
 C21(H21), C22(H22)

3. HPLC data for mixture of **3a** and **4a,b**

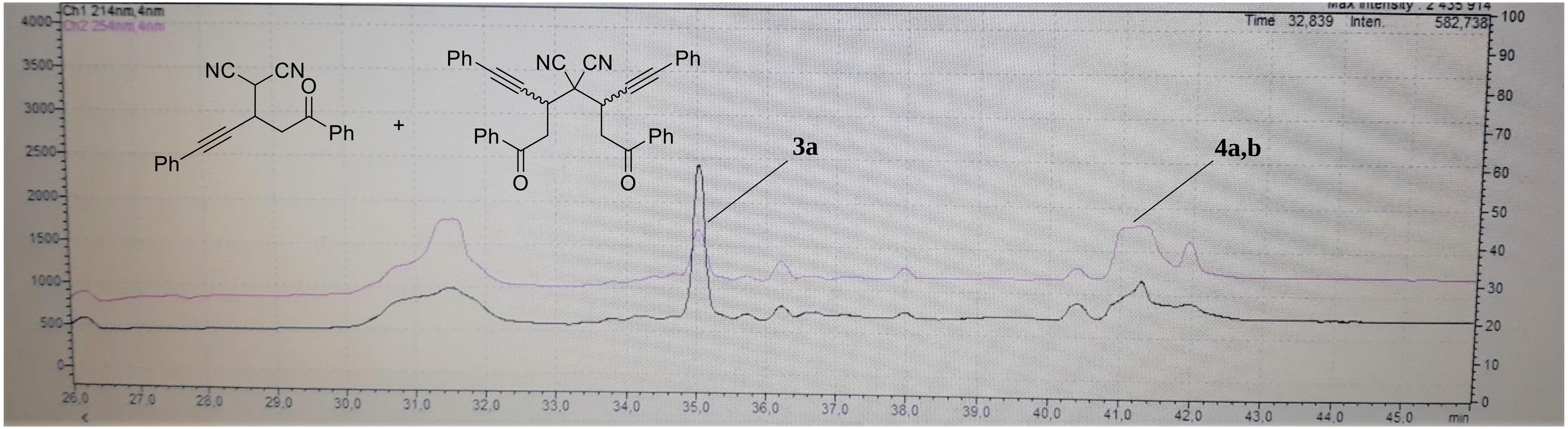

Figure S51. HPLC of the compounds **3a**, **4a,b**.

4. Photoluminescent spectra of cyclohexanes **2a**, **2b**, **2c**, **2e**

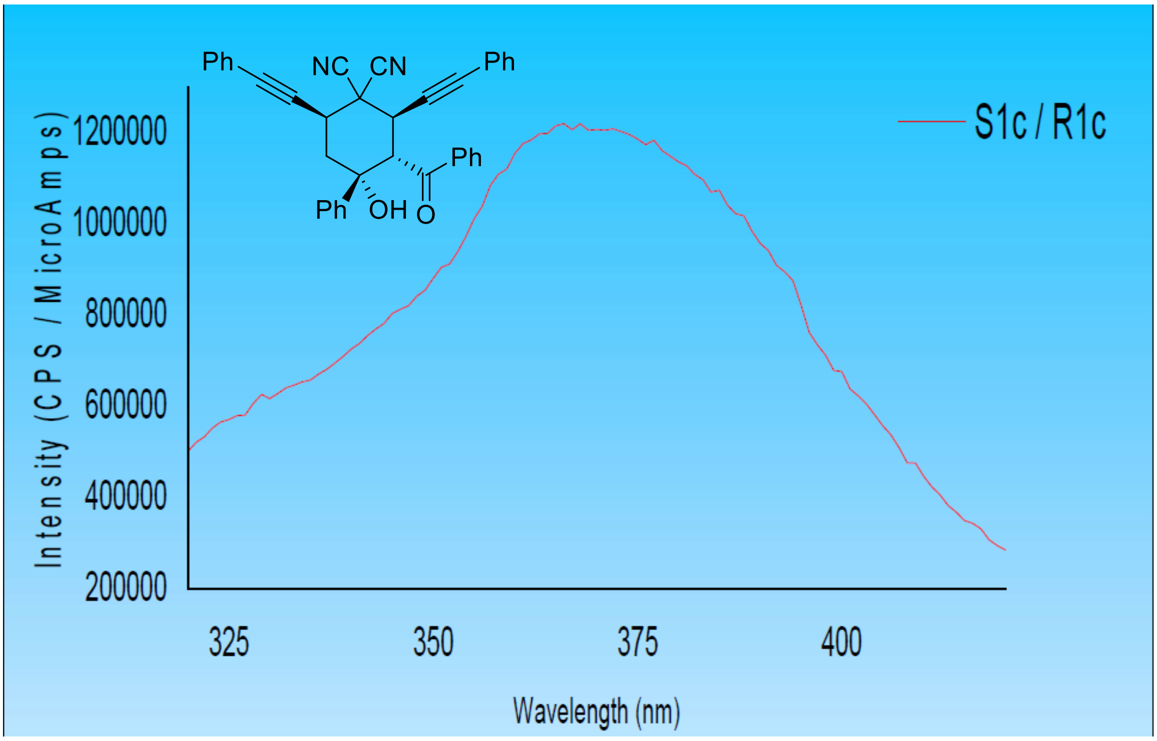

Figure S52. Photoluminescent spectrum of compound **2a** (KBr, excitation at 446 nm).

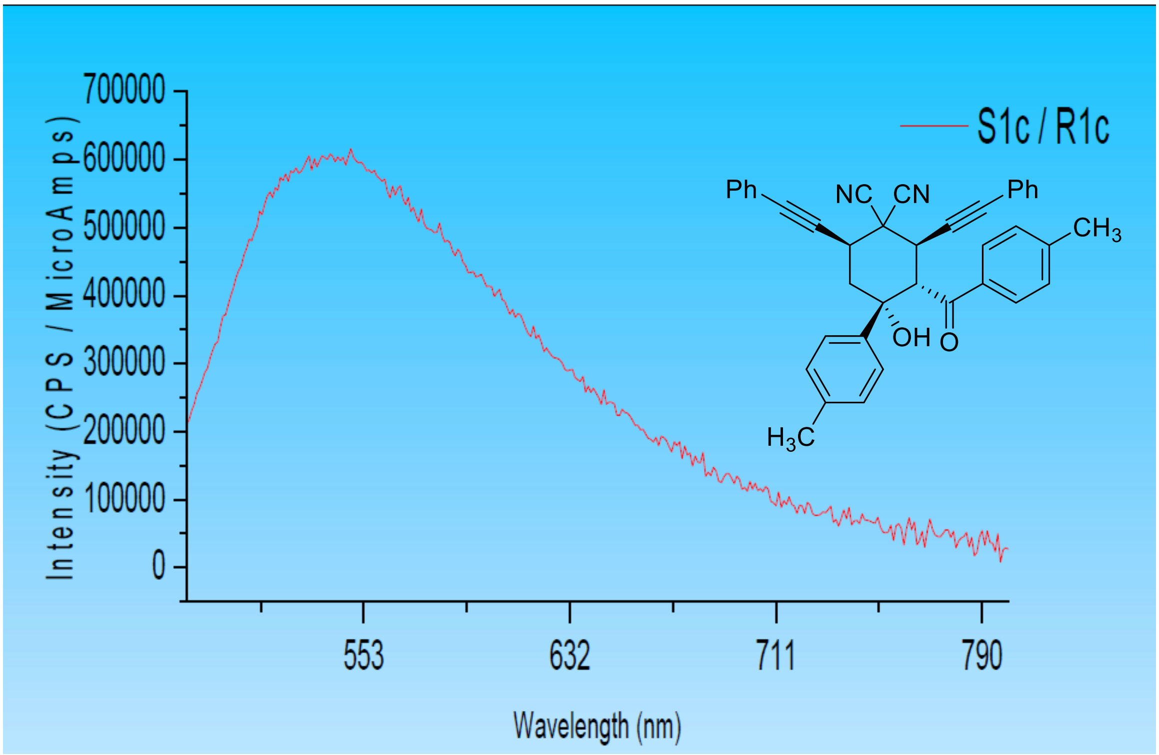

Figure S53. Photoluminescent spectrum of compound **2b** (KBr, excitation at 465 nm).

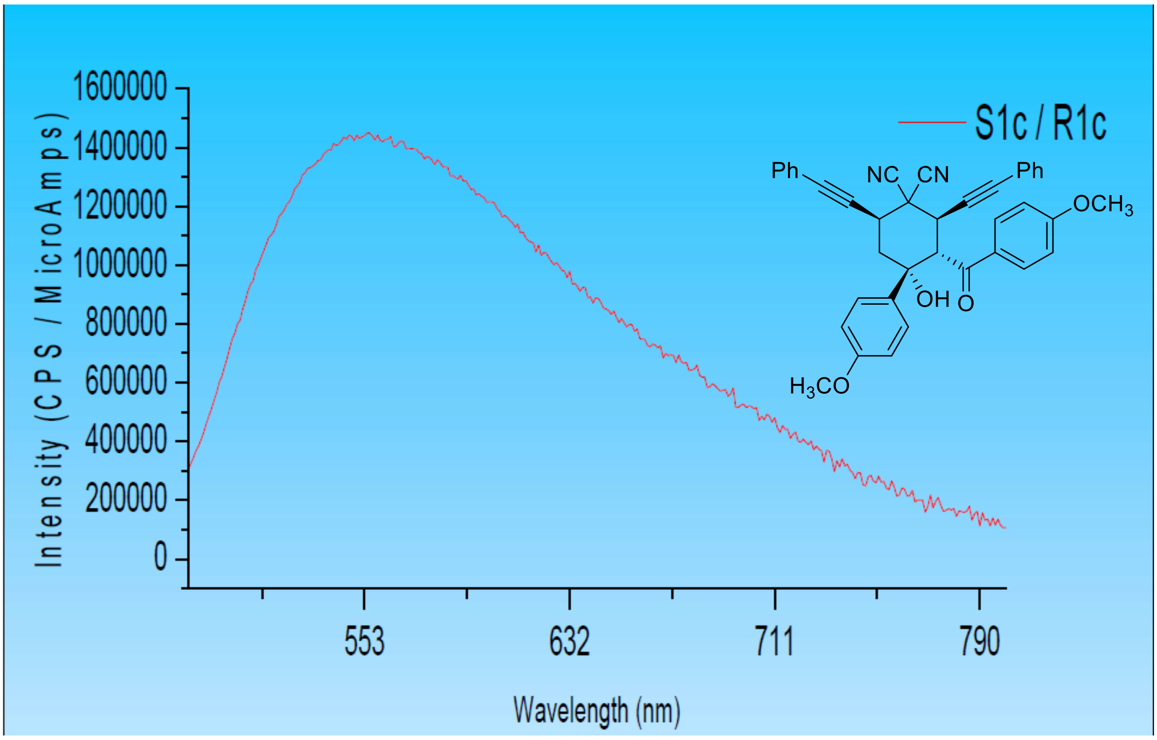

Figure S54. Photoluminescent spectrum of compound **2c** (KBr, excitation at 465 nm).

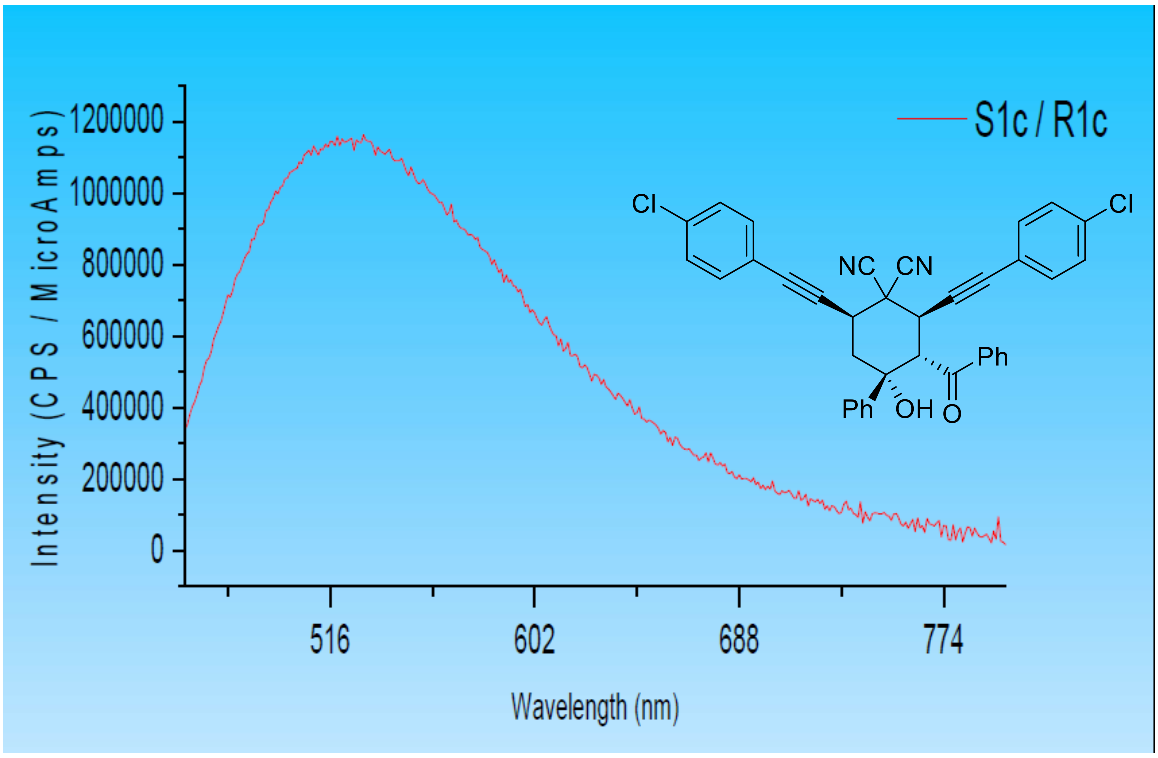

Figure S55. Photoluminescent spectrum of compound **2c** (KBr, excitation at 436 nm).

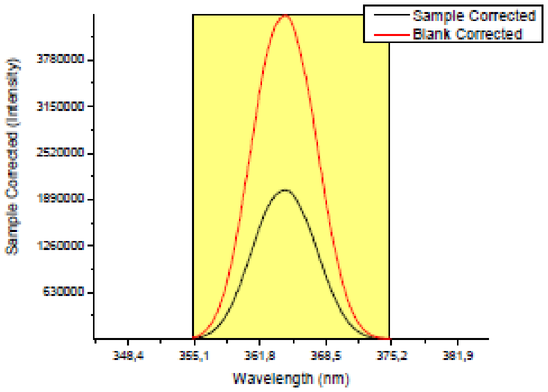

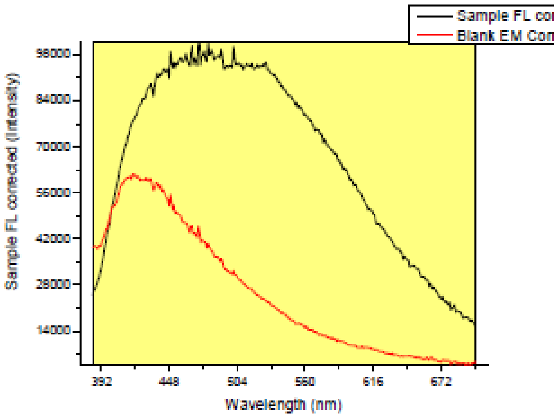

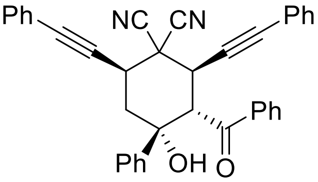

Excitation / Scatter			Emission / Fluorescence		
Enter Start and End Wavelengths			Enter Start and End Wavelengths		
Start Wavelength	355,000	Area Balance Factor	Start Wavelength	385,050	
End Wavelength	374,990	49,7	End Wavelength	700,030	
Area		Err	Area		Err
La 1,60E+07		3999,38083	***Ea*** 2,10E+07		92,18426
Lc 3,51E+07		5924,46797	***Ec*** 8052237,24		57,0955

Quantum Yield	**1,36**
Abs Error±	0,001
Relative Err ±	3,74211E-4

Sample Source
DfltEm9G

HORIBA *Blank Source*
DfltEm11G

Figure S56. Quantum yield data of compound **2a** (KBr).

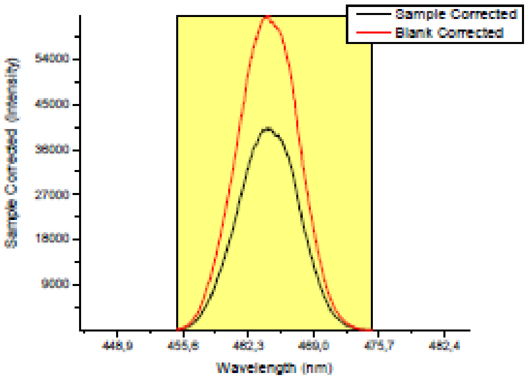

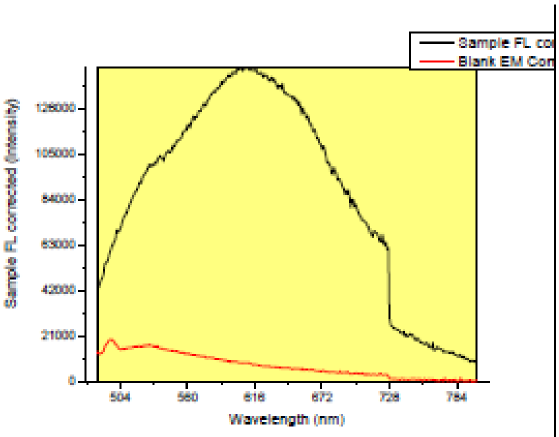

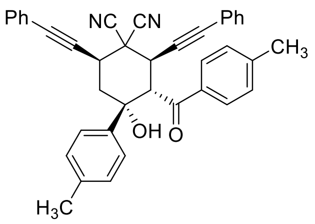

Excitation / Scatter				Emission / Fluorescence		
Enter Start and End Wavelengths				Enter Start and End Wavelengths		
Start Wavelength	455,000	Area Balance Factor		Start Wavelength	485,190	
End Wavelength	474,990	2869,3		End Wavelength	800,170	
Area		Err		Area		Err
La 313235,46		559,67442		*Ea* 2,72E+07		1,8175
Lc 487950,04		698,53421		*Ec* 2291941,84		0,52763

Sample Source DfltEm4G	***Quantum Yield***	**4,97**	HORIBA *Blank Source* DfltEm5G
	Abs Error±	0,025	
	Relative Err ±	0,00512	

Figure S57. Quantum yield data of compound **2b** (KBr).

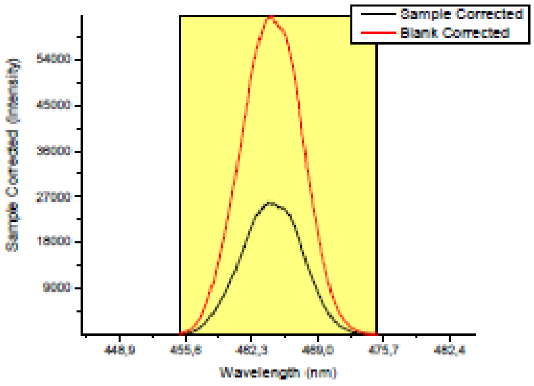

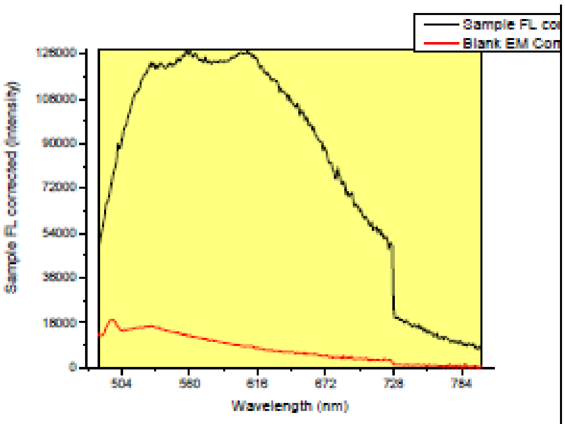

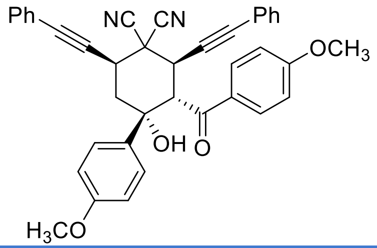

Excitation / Scatter			Emission / Fluorescence		
Enter Start and End Wavelengths			Enter Start and End Wavelengths		
Start Wavelength	455,000	Area Balance Factor	Start Wavelength	485,190	
End Wavelength	474,990	2869,3	End Wavelength	800,170	
Area		Err	Area		Err
La 202280,46		449,756	*Ea* 2,53E+07		1,75459
Lc 487950,04		698,53421	*Ec* 2291941,84		0,52763

Sample Source DfltEm8G	***Quantum Yield***	**2,81**	HORIBA *Blank Source* DfltEm5G
	Abs Error±	0,008	
	Relative Err ±	0,00291	

Figure S58. Quantum yield data of compound **2c** (KBr).

5. Data of DFT calculations of stereoisomers of **2a**

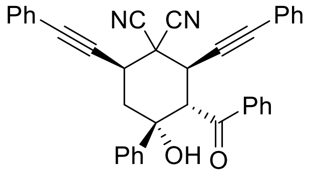

2a1

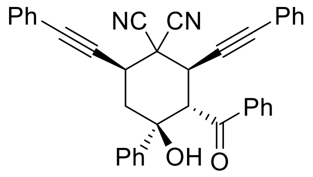

2a2

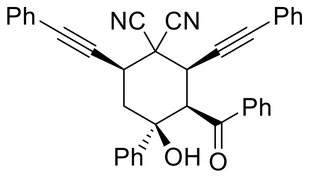

2a3

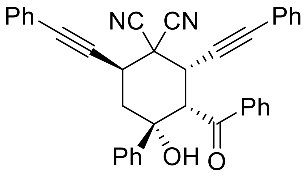

2a4

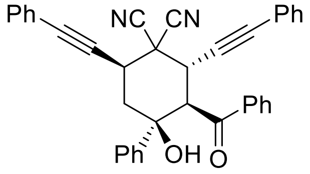

2a5

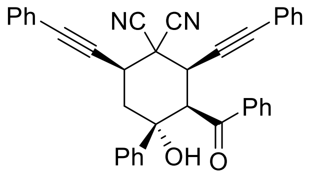

2a6

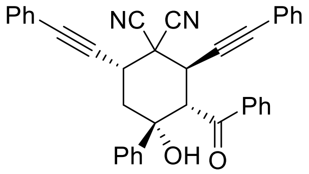

2a7

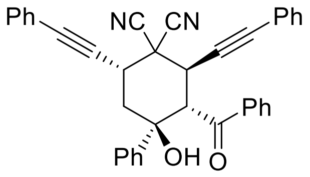

2a8

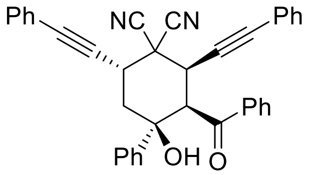

2a9

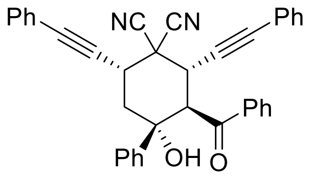

2a10

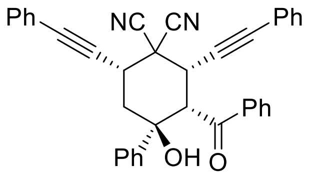

2a11

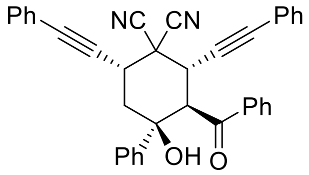

2a12

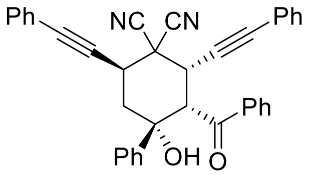

2a13

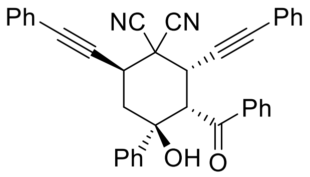

2a14

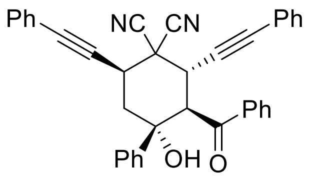

2a15

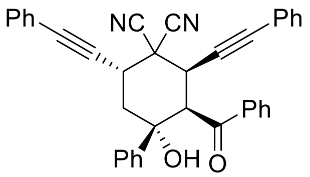

2a16

Table S20. Relative energies of diastereomers of 2a, kJ/mol

Pairs of enantiomers		**ΔG, kJ/mol**
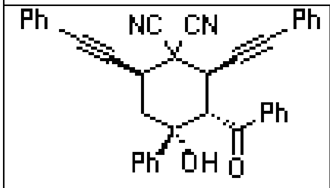<br>2a1	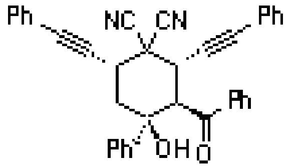<br>2a12	**min**
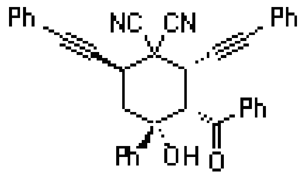<br>2a4	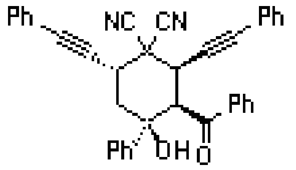<br>2a9	**7**
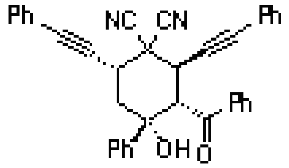<br>2a7	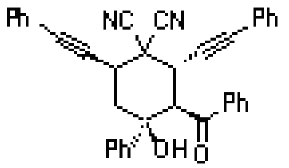<br>2a5	**12**
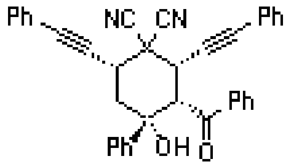<br>2a10	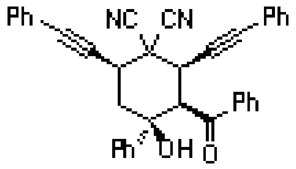<br>2a3	**28**
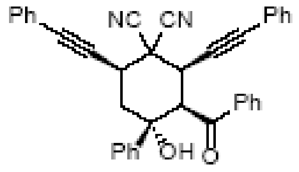<br>2a6	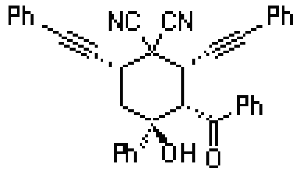<br>2a11	**28**
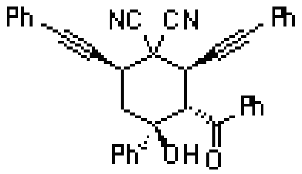<br>2a2	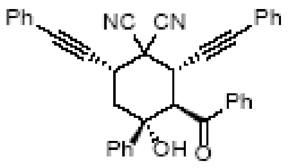<br>2a10	**38**

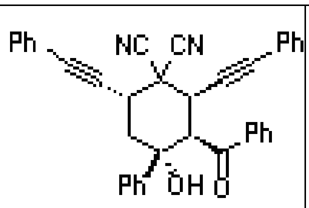<br>2a16	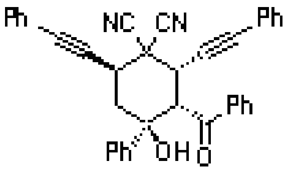<br>2a14	**41**
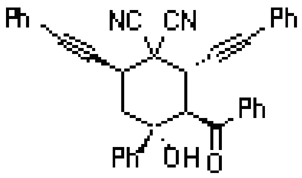<br>2a15	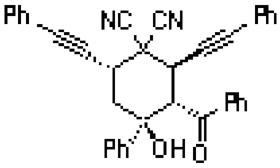<br>2a8	**49**

2a1
Energy **E(B3LYP) = -1685.40986436 h, G^{298} =-1684.95764h, μ= 6.67 D**

Cartesian coordinates, Å

N	atom	x	y	z
1	C	0.789521	-5.833920	0.833374
2	C	0.585762	-4.733393	1.669501
3	C	0.246150	-3.493222	1.127629
4	C	0.104786	-3.330792	-0.259855
5	C	0.304097	-4.441539	-1.088098
6	C	0.645666	-5.683182	-0.546412
7	C	-0.285173	-1.971053	-0.865132
8	C	0.746086	-0.864273	-0.441882
9	C	0.340959	0.518194	-1.035023
10	C	-1.117501	0.918333	-0.562365
11	C	-2.149021	-0.215345	-0.955135
12	C	-1.694586	-1.574936	-0.384104
13	C	1.301697	1.570508	-0.709936
14	C	2.088855	2.449868	-0.428528
15	C	3.015478	3.491238	-0.109774
16	C	2.844093	4.781605	-0.648542
17	C	3.746731	5.795585	-0.336105
18	C	4.828389	5.538461	0.511265
19	C	5.005344	4.260205	1.048599
20	C	4.107034	3.239709	0.744339
21	C	2.150499	-1.235945	-0.943785
22	O	2.280008	-1.514745	-2.139686
23	C	-1.505999	2.179261	-1.224283
24	N	-1.810822	3.152174	-1.777059
25	C	-1.131676	1.136415	0.899259
26	N	-1.131371	1.266707	2.051855
27	C	-3.505820	0.126860	-0.535773
28	C	-4.630471	0.392755	-0.166175
29	C	-5.959366	0.705117	0.260543

30	C	-6.343273	0.502082	1.600791
31	C	-7.639723	0.804431	2.011741
32	C	-8.568156	1.310658	1.097560
33	C	-8.195142	1.515665	-0.233960
34	C	-6.900840	1.216831	-0.654117
35	O	-0.370726	-2.029272	-2.284080
36	C	3.319019	-1.281365	-0.029376
37	C	3.272672	-0.850294	1.308707
38	C	4.413979	-0.913952	2.107517
39	C	5.609258	-1.413101	1.584561
40	C	5.666152	-1.845436	0.254901
41	C	4.532283	-1.776610	-0.545947
42	H	4.241315	2.245916	1.160129
43	H	2.002796	4.976599	-1.306345
44	H	5.845022	4.057678	1.707276
45	H	3.606221	6.787796	-0.755157
46	H	5.530731	6.331368	0.752073
47	H	2.361064	-0.449093	1.736185
48	H	4.562208	-2.105955	-1.578747
49	H	4.367842	-0.576577	3.138430
50	H	6.594644	-2.234166	-0.152561
51	H	6.494891	-1.465921	2.211232
52	H	0.738689	-0.787443	0.645102
53	H	-5.619955	0.108272	2.308113
54	H	-6.608028	1.374811	-1.687511
55	H	-7.926089	0.644410	3.047295
56	H	-8.913941	1.909448	-0.946843
57	H	-9.578227	1.544777	1.421587
58	H	-1.736520	-1.558340	0.708942
59	H	-2.398704	-2.339897	-0.724385
60	H	0.085684	-2.655234	1.801145
61	H	0.182367	-4.330970	-2.159728
62	H	0.689959	-4.837925	2.745938
63	H	0.797387	-6.532563	-1.207066
64	H	1.055091	-6.799300	1.255135
65	H	0.275247	0.403282	-2.122703
66	H	-2.106127	-0.278731	-2.047960
67	H	0.550966	-1.984828	-2.618913

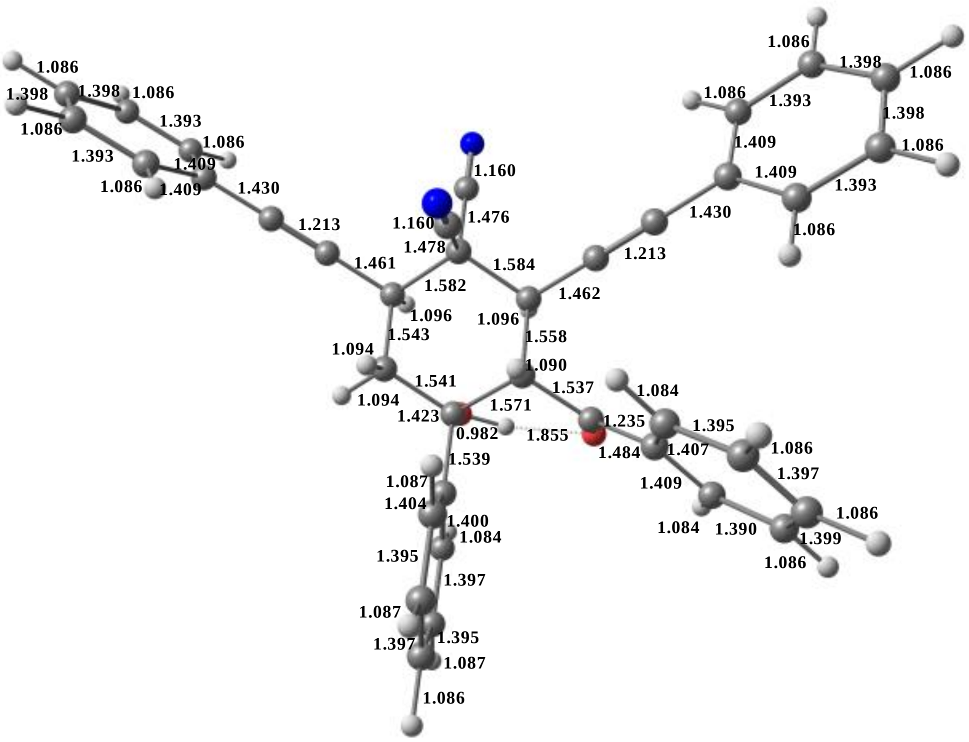
1.086
1.398
1.086
1.086
1.398
1.398
1.086
1.086
1.393
1.393
1.086
1.086
1.398
1.393
1.409
1.409
1.086
1.086
1.409
1.409
1.160
1.430
1.409
1.393
1.476
1.430
1.213
1.160
1.086
1.478
1.213
1.461
1.584
1.582
1.462
1.096
1.096
1.543
1.558
1.094
1.090
1.541
1.537
1.094
1.084
1.571
1.423
1.235
1.395
0.982
1.855
1.086
1.484
1.407
1.539
1.397
1.087
1.409
1.086
1.404
1.400
1.084
1.390
1.399
1.084
1.086
1.395
1.397
1.087
1.395
1.397
1.087
1.086

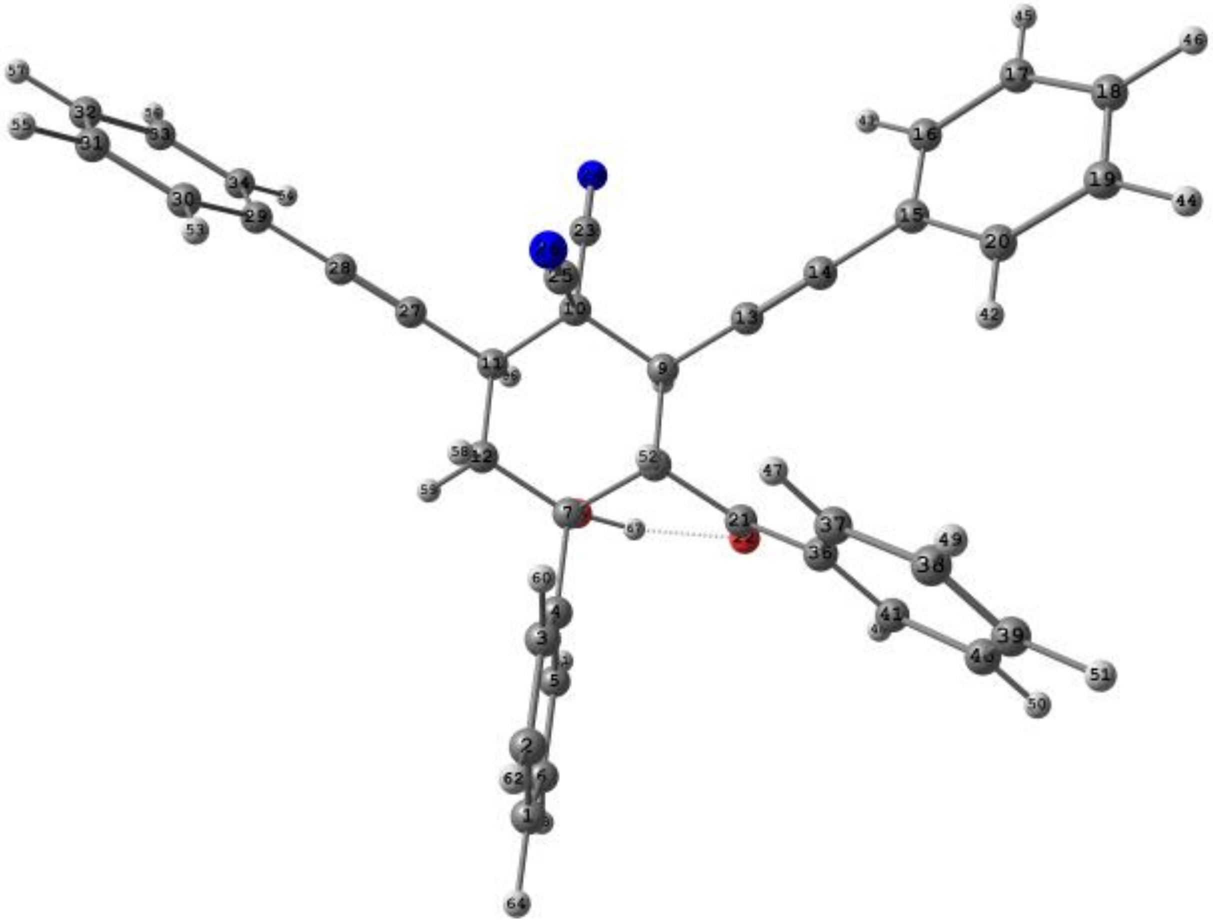

2a2

Energy **E(B3LYP) =-1685.39403493 h, G^{298} =-1684.943124h, μ= 6.96 D**

Cartesian coordinates, Å

N	atom	x	y	z
1	C	-2.656713	4.906350	0.999088
2	C	-3.048087	3.665635	0.458811
3	C	-4.303825	3.553559	-0.169547
4	C	-5.146230	4.660245	-0.251695
5	C	-4.750837	5.888306	0.286379
6	C	-3.505750	6.007368	0.910585
7	C	-2.178742	2.533572	0.547246
8	C	-1.443565	1.572682	0.632144
9	C	-0.531887	0.429281	0.701738
10	C	-1.020690	-0.738076	-0.209253
11	C	-2.426033	-1.176924	0.245603
12	C	-3.390513	-1.764346	-0.732976
13	C	-3.252543	-1.658389	-2.128959
14	C	-4.222271	-2.198667	-2.974364
15	C	-5.332633	-2.855291	-2.440874
16	C	-5.479208	-2.967804	-1.053603
17	C	-4.520845	-2.420910	-0.208236
18	H	-4.607614	2.598658	-0.587197
19	H	-1.689035	4.995084	1.483133
20	H	-6.113070	4.564065	-0.737415
21	H	-3.194990	6.960048	1.329831
22	H	-5.410257	6.748923	0.219523
23	H	-2.397609	-1.157822	-2.566954
24	H	-4.624976	-2.493601	0.868906
25	H	-4.108929	-2.104174	-4.050140
26	H	-6.341042	-3.480191	-0.636214
27	H	-6.083044	-3.278311	-3.102701
28	C	0.937859	0.886672	0.338216
29	C	1.398549	1.850957	1.357370
30	N	1.757065	2.583723	2.181678
31	C	0.960848	1.563070	-0.976595
32	N	0.973759	2.065794	-2.021688
33	H	-1.091817	-0.334003	-1.222587
34	C	1.900137	-0.365211	0.317001
35	C	3.282227	0.023308	0.044596
36	C	4.427312	0.340479	-0.199248
37	C	5.779791	0.710863	-0.480143
38	C	6.492733	0.072208	-1.513779
39	C	6.414840	1.717261	0.273721
40	C	7.810995	0.434064	-1.782439
41	C	7.732781	2.073623	-0.004100
42	C	8.434080	1.434105	-1.030412
43	H	6.005822	-0.703418	-2.096806
44	H	5.867449	2.211247	1.070547
45	H	8.352735	-0.064932	-2.580866
46	H	8.213680	2.851407	0.582191
47	H	9.461847	1.714015	-1.243330

48	C	1.382680	-1.390060	-0.706027
49	H	1.343862	-0.933165	-1.701067
50	H	2.088930	-2.222014	-0.760957
51	C	-0.018042	-1.954026	-0.373900
52	O	-0.375322	-2.639715	-1.580075
53	C	0.084463	-2.969679	0.780354
54	C	0.596562	-4.242539	0.465095
55	C	-0.232140	-2.703639	2.120217
56	C	0.782267	-5.211251	1.449279
57	C	-0.042886	-3.674988	3.108596
58	C	0.465147	-4.930468	2.780548
59	H	0.845778	-4.473083	-0.565411
60	H	-0.667318	-1.758509	2.414053
61	H	1.175828	-6.185942	1.174196
62	H	-0.303208	-3.441958	4.137337
63	H	0.609072	-5.683340	3.550401
64	H	-0.481324	0.087658	1.738419
65	H	1.838619	-0.799341	1.323916
66	O	-2.743255	-1.054730	1.423928
67	H	-1.135527	-3.216402	-1.394174

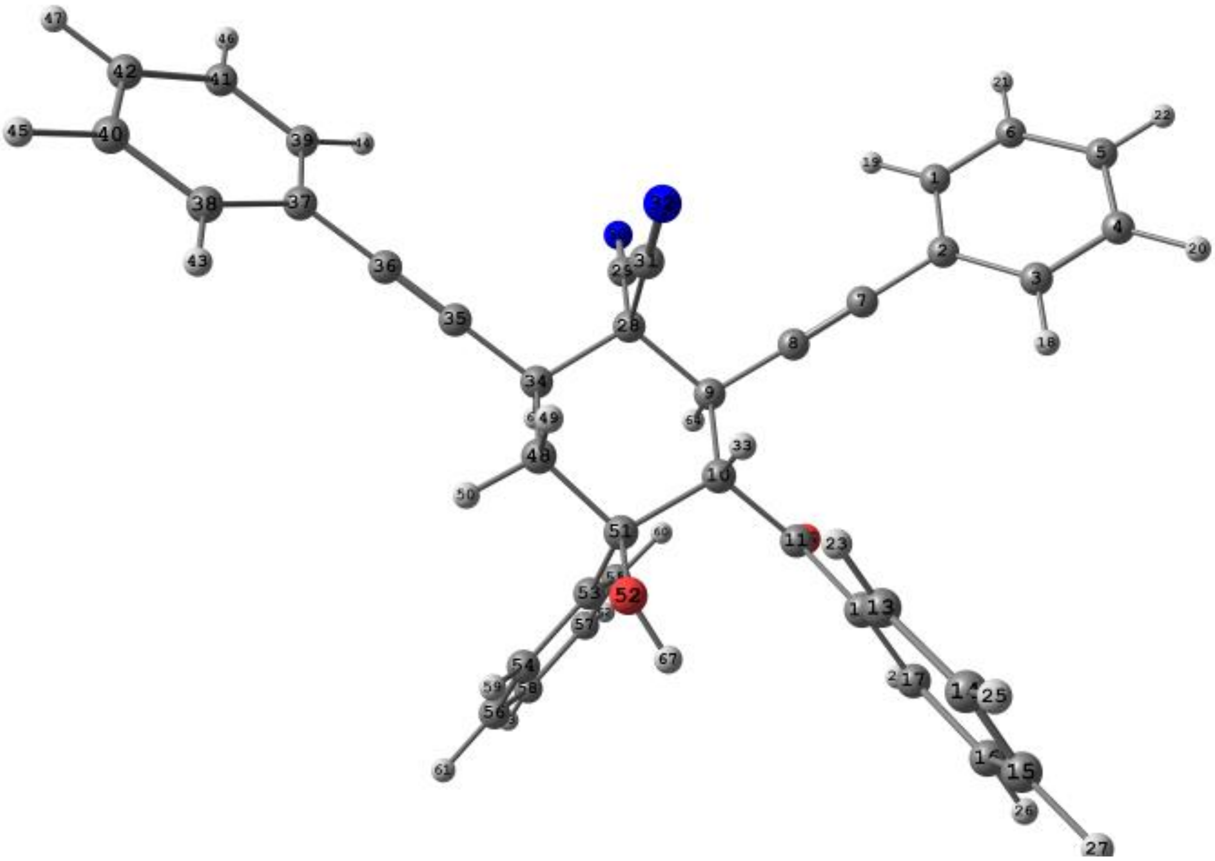

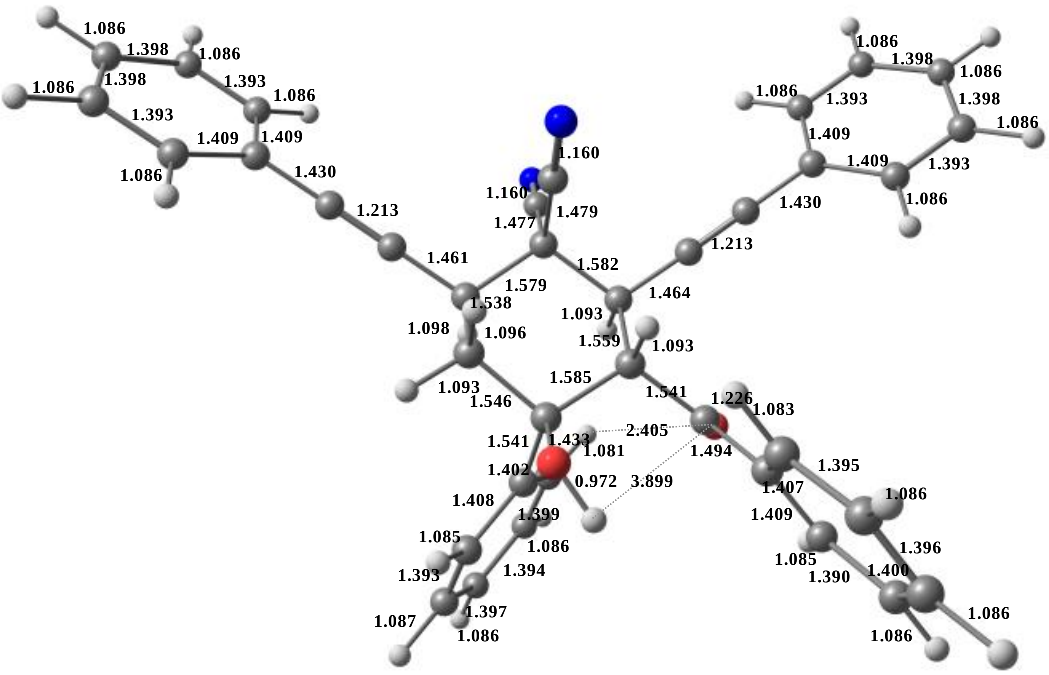

1.086
1.398
1.086
1.086
1.398
1.393
1.086
1.393
1.409
1.409
1.086
1.430
1.213
1.461
1.160
1.160
1.477
1.479
1.579
1.582
1.538
1.098
1.096
1.093
1.559
1.093
1.093
1.546
1.585
1.541
1.541
1.433
1.081
2.405
1.226
1.083
1.494
1.402
0.972
3.899
1.408
1.399
1.085
1.086
1.393
1.394
1.397
1.087
1.086
1.395
1.407
1.086
1.409
1.085
1.396
1.390
1.400
1.086
1.086
1.086
1.398
1.086
1.086
1.393
1.398
1.086
1.409
1.409
1.393
1.430
1.086
1.213
1.464

2a4

Energy **E(B3LYP) = -1685.40627784 h, G^{298} =-1684.954972 h, μ= 4.73 D**

Cartesian coordinates, Å

N	atom	x	y	z
1	C	-1.016764	5.170307	0.036990
2	C	-1.703914	4.009340	0.442085
3	C	-2.778222	4.123720	1.346234
4	C	-3.153872	5.374700	1.830663
5	C	-2.466199	6.522566	1.425873
6	C	-1.398576	6.416263	0.529798
7	C	-1.316683	2.727746	-0.058000
8	C	-0.994624	1.630838	-0.462935
9	C	-0.626117	0.337431	-1.035280
10	C	-1.163939	-0.907315	-0.247325
11	C	-2.687154	-0.770288	-0.088638
12	C	-3.600584	-1.195215	-1.183099
13	C	-3.161284	-1.845876	-2.350026
14	C	-4.073583	-2.207039	-3.340598
15	C	-5.431722	-1.926009	-3.178689
16	C	-5.880621	-1.280562	-2.020869
17	C	-4.973392	-0.918248	-1.032335
18	H	-3.308131	3.229113	1.658518
19	H	-0.188506	5.083213	-0.659464
20	H	-3.984023	5.453786	2.526758
21	H	-0.862101	7.306160	0.213233
22	H	-2.761126	7.495958	1.807307
23	H	-2.114118	-2.083529	-2.499949
24	H	-5.306667	-0.415053	-0.131394
25	H	-3.722800	-2.709958	-4.236528
26	H	-6.936493	-1.060770	-1.894078
27	H	-6.140054	-2.209040	-3.952017
28	C	0.950128	0.228701	-1.206580
29	C	1.459308	1.439095	-1.880338
30	N	1.858601	2.392651	-2.406021
31	C	1.270423	-0.928856	-2.067516
32	N	1.498041	-1.853945	-2.729639
33	H	-0.953465	-1.774168	-0.876662
34	C	1.636809	0.061973	0.205618
35	C	3.092933	0.024040	0.097317
36	C	4.301872	-0.038603	0.015204
37	C	5.727809	-0.102757	-0.075988
38	C	6.360132	-1.271371	-0.544328
39	C	6.517352	1.000799	0.303117
40	C	7.749478	-1.330316	-0.629922
41	C	7.906081	0.932092	0.214998
42	C	8.525558	-0.231160	-0.251035
43	H	5.754518	-2.123423	-0.837381
44	H	6.033355	1.902954	0.664293
45	H	8.227395	-2.235894	-0.992539
46	H	8.506038	1.788164	0.510282
47	H	9.608599	-0.280878	-0.318590

48	C	1.081787	-1.193850	0.912897
49	H	1.363257	-2.091696	0.354342
50	H	1.551083	-1.266719	1.898324
51	C	-0.444998	-1.139001	1.122192
52	O	-0.662388	-0.038098	1.994020
53	C	-0.912348	-2.465387	1.754195
54	C	-0.850936	-3.674766	1.043991
55	C	-1.377666	-2.489158	3.074251
56	C	-1.256271	-4.872385	1.635232
57	C	-1.783651	-3.686866	3.667311
58	C	-1.727381	-4.882803	2.950003
59	H	-0.479353	-3.698523	0.022851
60	H	-1.414686	-1.563172	3.636696
61	H	-1.201794	-5.796566	1.066409
62	H	-2.143846	-3.682219	4.692673
63	H	-2.044266	-5.814526	3.410395
64	H	-1.046973	0.283912	-2.046925
65	H	1.329127	0.943318	0.779696
66	O	-3.141692	-0.267922	0.940716
67	H	-1.623848	0.146081	1.948681

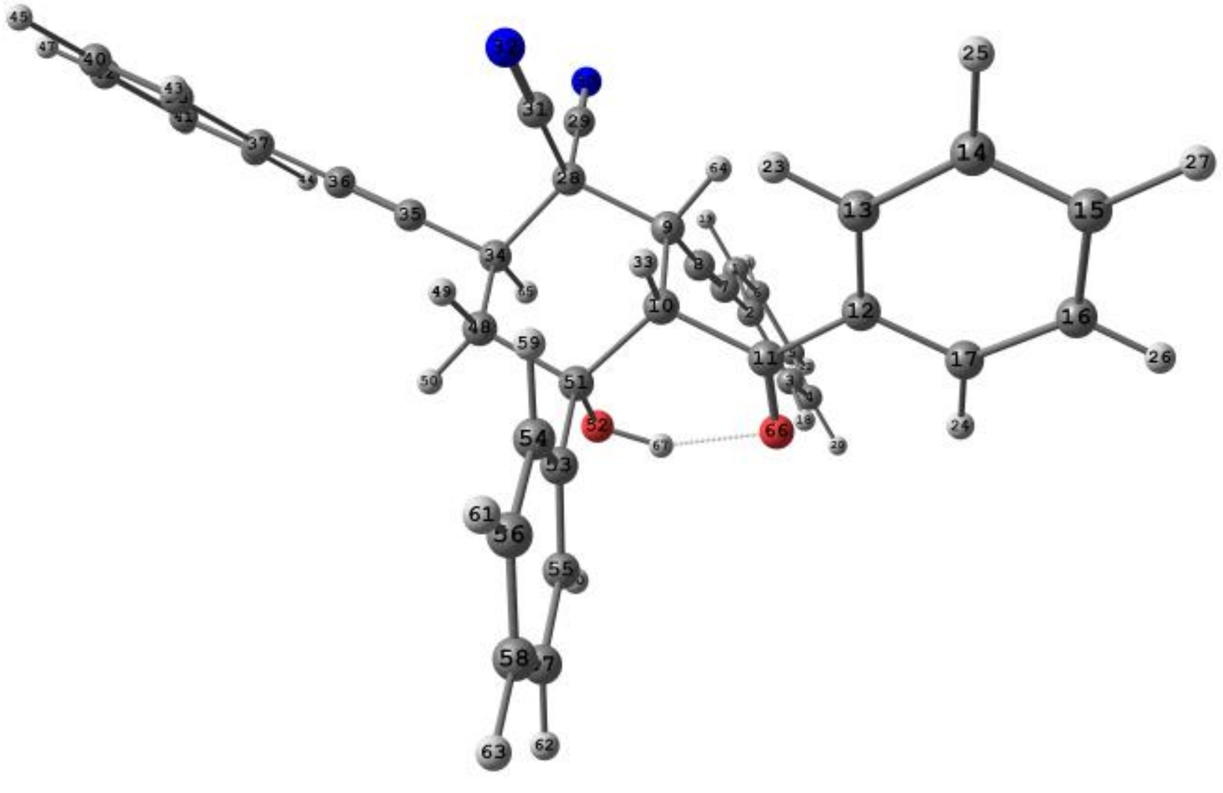

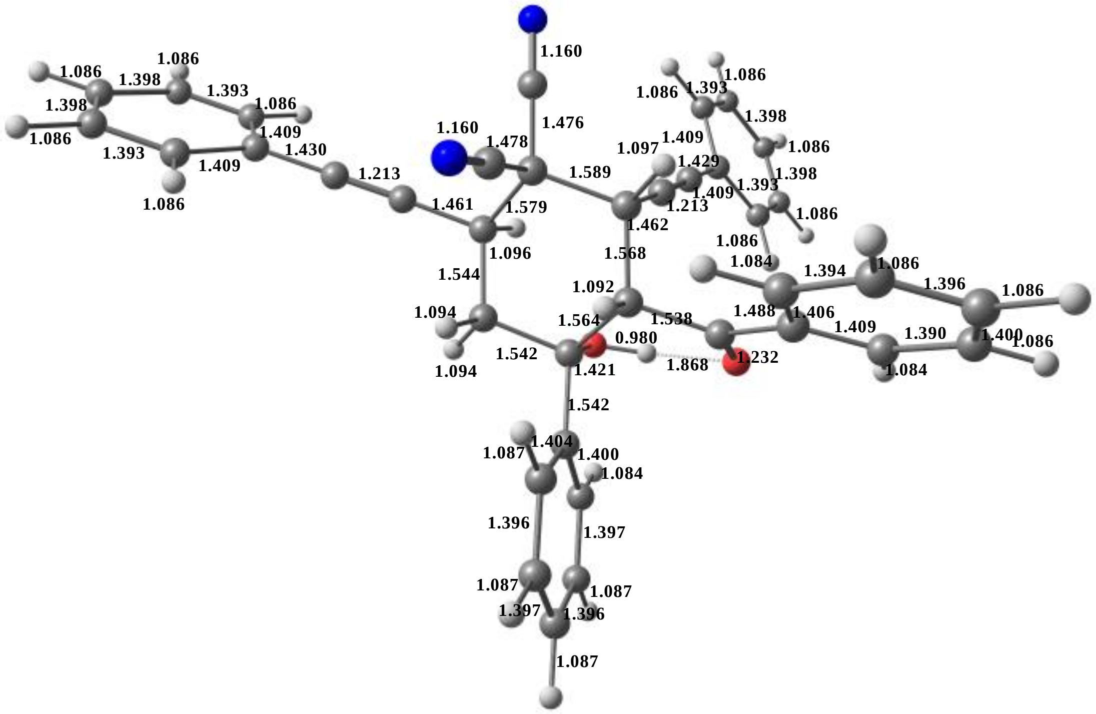
1.160
1.086
1.086
1.398
1.393
1.086
1.398
1.086
1.393
1.160
1.478
1.476
1.409
1.086
1.393
1.430
1.409
1.409
1.097
1.429
1.213
1.589
1.398
1.086
1.409
1.393
1.213
1.461
1.579
1.462
1.086
1.086
1.086
1.096
1.568
1.084
1.394
1.086
1.544
1.092
1.396
1.086
1.094
1.564
1.538
1.488
1.406
1.409
1.390
1.400
1.086
0.980
1.542
1.232
1.094
1.421
1.868
1.084
1.542
1.087
1.404
1.400
1.084
1.396
1.397
1.087
1.087
1.397
1.396
1.087

a6

Energy **E(B3LYP) = -1685.3982108 h, G^{298} =-1684.946998 h, μ= 8.83 D**

Cartesian coordinates, Å

N	atom	x	y	z
1	C	-1.118592	5.950046	0.032181
2	C	-0.272086	5.231169	0.877374
3	C	0.179413	3.961215	0.510950
4	C	-0.206844	3.389461	-0.710046
5	C	-1.059375	4.121402	-1.551982
6	C	-1.511296	5.389368	-1.185130
7	C	0.248120	1.991256	-1.151848
8	C	-0.858553	0.930796	-0.848866
9	C	-0.403767	-0.495678	-1.302449
10	C	1.041818	-0.929857	-0.813070
11	C	2.087302	0.223276	-1.105081
12	C	1.601724	1.575305	-0.541245
13	C	-1.397181	-1.521993	-0.993102
14	C	-2.218195	-2.374179	-0.725743
15	C	-3.186195	-3.381042	-0.418524
16	C	-2.876912	-4.743145	-0.601567
17	C	-3.821441	-5.721802	-0.299870
18	C	-5.081680	-5.358918	0.183941
19	C	-5.395959	-4.009190	0.366820
20	C	-4.457949	-3.022683	0.069937
21	C	-1.340111	0.983237	0.608298
22	O	-0.522154	0.856665	1.514735
23	C	1.434108	-2.117716	-1.604564
24	N	1.739513	-3.030117	-2.252541
25	C	1.085764	-1.357332	0.602414
26	N	1.175468	-1.760209	1.686123
27	C	3.416216	-0.130005	-0.610777
28	C	4.510718	-0.414282	-0.171424
29	C	5.796006	-0.755908	0.354709
30	C	5.902706	-1.371542	1.617578
31	C	7.154060	-1.704843	2.131273
32	C	8.312268	-1.429085	1.398752
33	C	8.214923	-0.817628	0.145488
34	C	6.967712	-0.482610	-0.377768
35	O	0.347182	1.941787	-2.586135
36	C	-2.782889	1.203424	0.923343
37	C	-3.808023	1.191269	-0.038972
38	C	-5.135838	1.387646	0.342583
39	C	-5.456034	1.607009	1.683996
40	C	-4.443898	1.623638	2.650038
41	C	-3.121490	1.419065	2.272937
42	H	-4.699206	-1.973548	0.210781
43	H	-1.897329	-5.021136	-0.978066
44	H	-6.374478	-3.723743	0.742361
45	H	-3.573482	-6.769603	-0.443314
46	H	-5.815794	-6.124777	0.417413
47	H	-3.591585	1.014734	-1.086271

48	H	-2.327278	1.424694	3.011443
49	H	-5.918552	1.371670	-0.409924
50	H	-4.689508	1.794863	3.694043
51	H	-6.490216	1.764889	1.976611
52	H	-1.682521	1.183716	-1.516270
53	H	5.000645	-1.583745	2.183246
54	H	6.889364	-0.008799	-1.351436
55	H	7.225497	-2.179963	3.105538
56	H	9.112738	-0.601806	-0.426681
57	H	9.286409	-1.689496	1.802802
58	H	1.533944	1.512751	0.545519
59	H	2.354710	2.332047	-0.786967
60	H	0.830948	3.422623	1.189734
61	H	-1.356820	3.696493	-2.505200
62	H	0.042638	5.656917	1.826172
63	H	-2.168119	5.939418	-1.853531
64	H	-1.467709	6.938299	0.318294
65	H	-0.295961	-0.419452	-2.390365
66	H	2.115470	0.309127	-2.196984
67	H	0.959811	2.643609	-2.866333

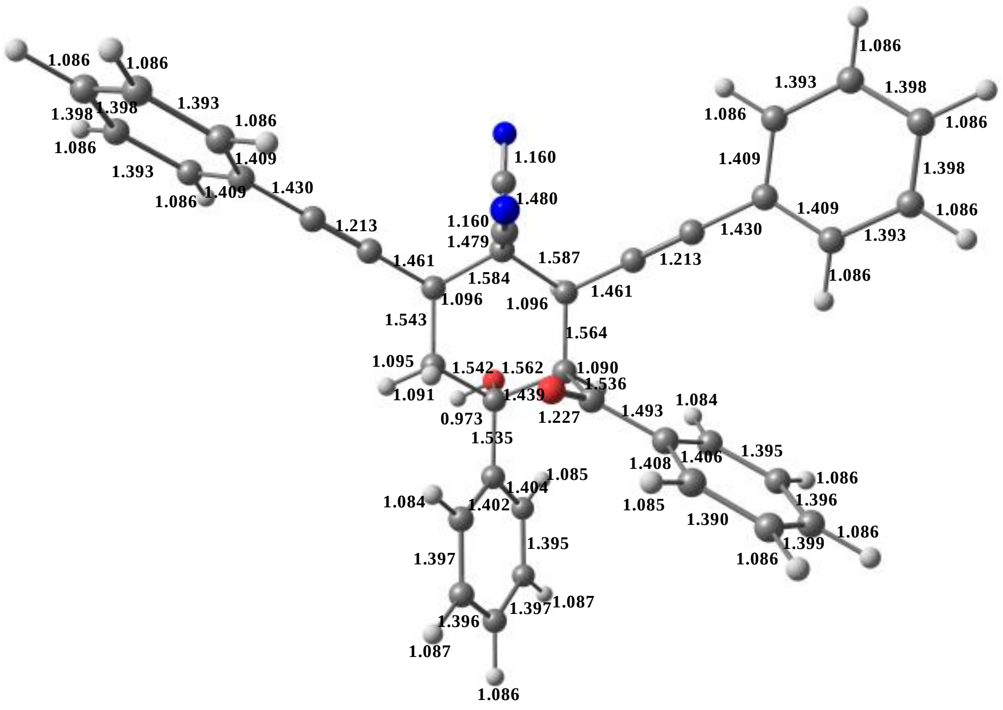

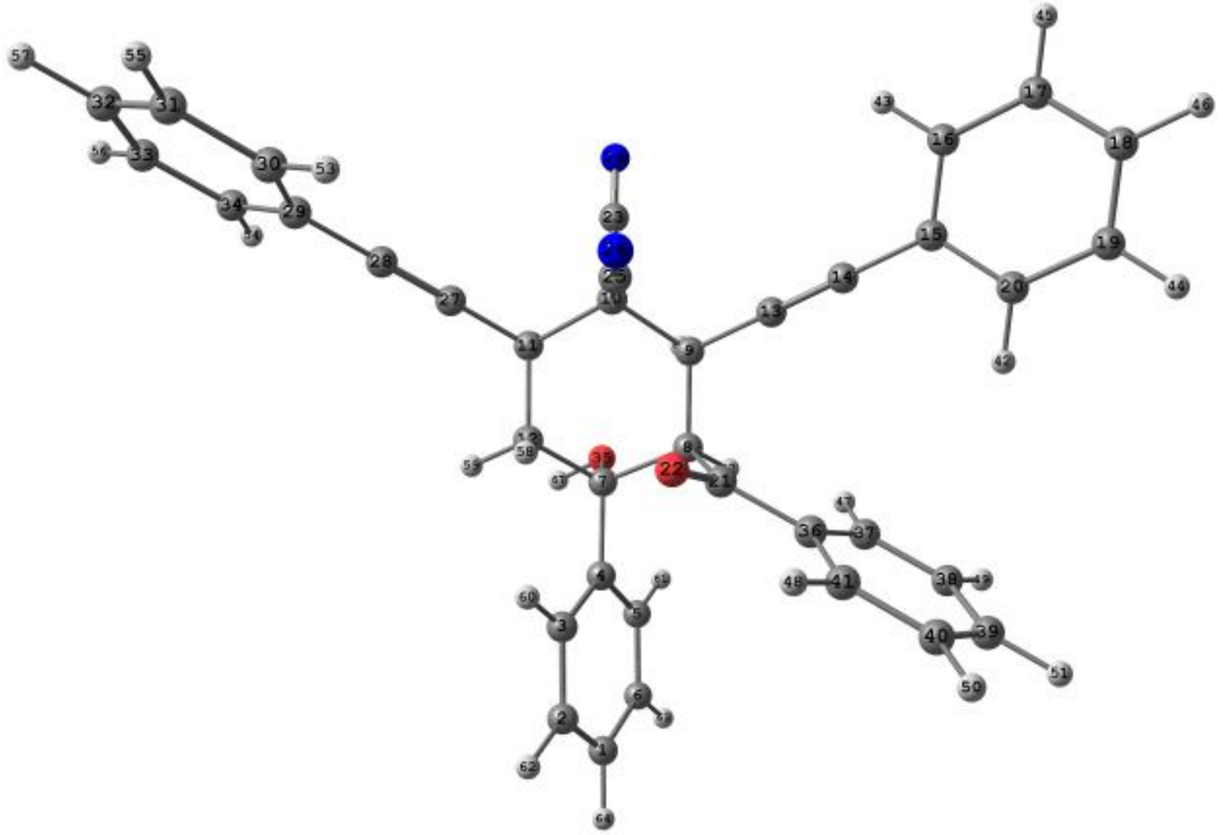

a7

Energy **E(B3LYP) =-1685.4051446 h, G^{298} =-1684.95293 h, μ= 6.23 D**

Cartesian coordinates, Å

N	atom	x	y	z
1	C	-0.595680	6.001544	0.401678
2	C	-1.080095	5.101173	1.353757
3	C	-0.640809	3.776705	1.355414
4	C	0.289904	3.327140	0.404830
5	C	0.773541	4.239599	-0.540207
6	C	0.333466	5.565552	-0.543586
7	C	0.796478	1.871294	0.403597
8	C	-0.413706	0.875341	0.295291
9	C	0.080793	-0.600867	0.372407
10	C	0.812838	-0.847652	1.753253
11	C	2.045468	0.141284	1.911860
12	C	1.576575	1.603409	1.707756
13	C	-0.996458	-1.567470	0.169993
14	C	-1.888285	-2.376599	0.021053
15	C	-2.927419	-3.341711	-0.162205
16	C	-2.610600	-4.710082	-0.272648
17	C	-3.622899	-5.650696	-0.448825
18	C	-4.958136	-5.243001	-0.519048
19	C	-5.279938	-3.886950	-0.411237
20	C	-4.275187	-2.938407	-0.232643
21	C	-1.172137	1.098273	-1.023063
22	O	-0.523645	1.106195	-2.073615
23	C	1.273780	-2.247775	1.830221
24	N	1.638072	-3.346868	1.895232
25	C	-0.132991	-0.630129	2.868341
26	N	-0.869560	-0.427310	3.741469
27	C	3.158359	-0.271049	1.057222
28	C	4.113276	-0.642442	0.408076
29	C	5.227200	-1.043459	-0.393904
30	C	5.455935	-0.432502	-1.642988
31	C	6.541972	-0.819932	-2.424718
32	C	7.411228	-1.818816	-1.976088
33	C	7.190879	-2.430986	-0.738876
34	C	6.108063	-2.048755	0.050462
35	O	1.707890	1.639055	-0.659799
36	C	-2.640604	1.319942	-1.045758
37	C	-3.464493	1.175887	0.085303
38	C	-4.839054	1.389102	-0.014772
39	C	-5.405237	1.755015	-1.238245
40	C	-4.594690	1.902532	-2.369318
41	C	-3.225435	1.682844	-2.274688
42	H	-4.522017	-1.884695	-0.147461
43	H	-1.572413	-5.022555	-0.217413
44	H	-6.316638	-3.567118	-0.465255
45	H	-3.369199	-6.703592	-0.531975
46	H	-5.744880	-5.979191	-0.657001
47	H	-3.053626	0.882009	1.044281

48	H	-2.585385	1.791265	-3.143366
49	H	-5.466210	1.272310	0.863764
50	H	-5.033316	2.187843	-3.320849
51	H	-6.475503	1.925705	-1.310831
52	H	-1.078766	1.057511	1.138856
53	H	4.776828	0.341821	-1.986398
54	H	5.934616	-2.521812	1.012150
55	H	6.710137	-0.342510	-3.385902
56	H	7.864355	-3.207594	-0.387673
57	H	8.256835	-2.119013	-2.588410
58	H	0.960663	1.889017	2.565674
59	H	2.457460	2.251746	1.716350
60	H	-1.029937	3.100059	2.112055
61	H	1.503001	3.906737	-1.269850
62	H	-1.800985	5.427903	2.098161
63	H	0.721010	6.256873	-1.287177
64	H	-0.938401	7.032538	0.399667
65	H	0.851986	-0.737519	-0.394416
66	H	2.375733	0.051159	2.954048
67	H	1.165701	1.504218	-1.466798

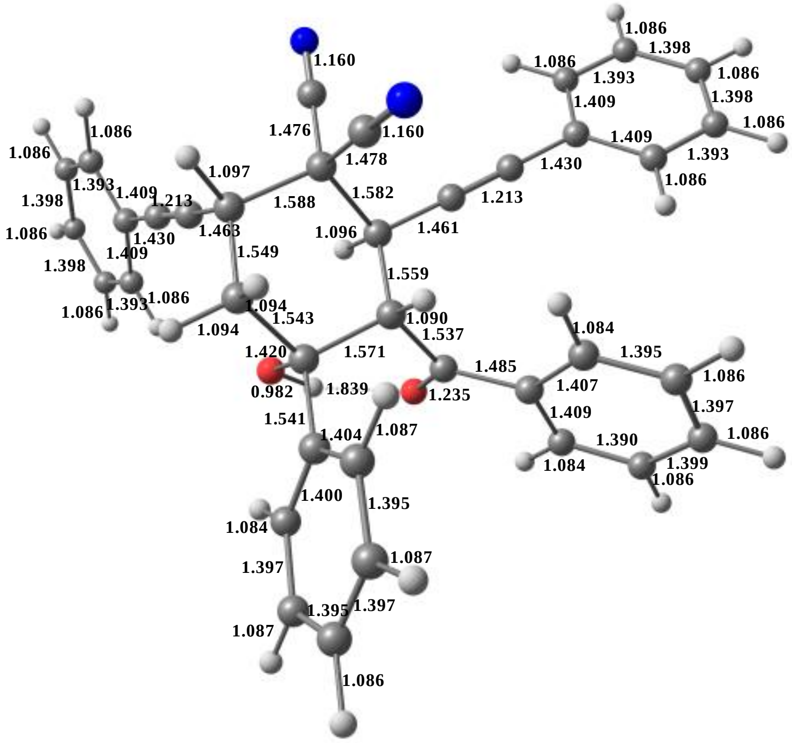

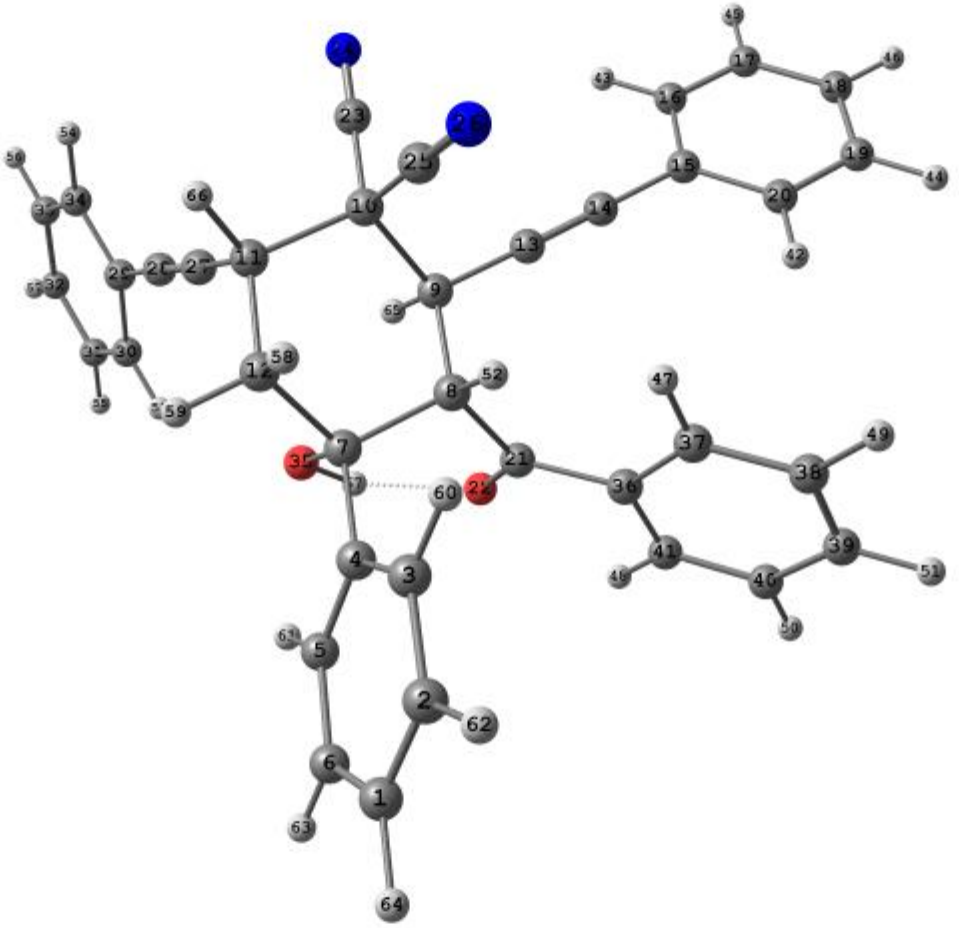

2a10

Energy **E(B3LYP) = -1685.39939797 h, G^{298} =-1684.946987 h, μ= 5.05 D**

Cartesian coordinates, Å

N	atom	x	y	z
1	C	-3.459711	2.816241	0.378532
2	C	-2.275018	2.545346	-0.334165
3	C	-2.115467	3.073849	-1.630434
4	C	-3.120511	3.855598	-2.195860
5	C	-4.293826	4.119273	-1.483045
6	C	-4.459649	3.597196	-0.196943
7	C	-1.244806	1.748272	0.252923
8	C	-0.378498	1.051130	0.736683
9	C	0.720214	0.341732	1.387805
10	C	1.797358	-0.250456	0.415160
11	C	2.297889	0.866694	-0.514831
12	C	3.368429	1.796665	-0.064382
13	C	4.058118	1.651376	1.152759
14	C	5.047305	2.564780	1.514884
15	C	5.361113	3.630189	0.668671
16	C	4.682413	3.784096	-0.545564
17	C	3.694720	2.876013	-0.908428
18	H	-1.203013	2.866077	-2.180908
19	H	-3.584353	2.410318	1.377662
20	H	-2.988056	4.259174	-3.195680
21	H	-5.369938	3.799545	0.360280
22	H	-5.075363	4.728622	-1.927848
23	H	3.842116	0.829716	1.826578
24	H	3.158861	2.984032	-1.845041
25	H	5.573972	2.441366	2.456204
26	H	4.925014	4.612707	-1.204223
27	H	6.133064	4.339482	0.953104
28	C	0.209051	-0.794783	2.372844
29	C	-0.846921	-0.260657	3.253805
30	N	-1.667802	0.155322	3.959577
31	C	1.338266	-1.193496	3.241875
32	N	2.235003	-1.511158	3.906117
33	H	2.626671	-0.570449	1.048522
34	C	-0.306989	-2.086714	1.607241
35	C	-1.641159	-1.908713	1.036376
36	C	-2.779790	-1.856782	0.621926
37	C	-4.100758	-1.766423	0.082670
38	C	-5.226552	-2.022787	0.889742
39	C	-4.291076	-1.420699	-1.270256
40	C	-6.509486	-1.933679	0.353358
41	C	-5.577339	-1.335467	-1.798445
42	C	-6.688894	-1.590902	-0.989905
43	H	-5.084174	-2.289755	1.932462
44	H	-3.424432	-1.222241	-1.893279
45	H	-7.370891	-2.132868	0.984560
46	H	-5.713075	-1.068436	-2.842675
47	H	-7.690544	-1.523013	-1.404861

48	C	0.774124	-2.568495	0.607988
49	H	1.611674	-2.974056	1.183663
50	H	0.359148	-3.394802	0.024035
51	C	1.303215	-1.504537	-0.376239
52	O	0.215704	-1.162318	-1.220216
53	C	2.469054	-2.120178	-1.180094
54	C	3.683789	-2.466351	-0.567025
55	C	2.315162	-2.378967	-2.547297
56	C	4.718242	-3.044213	-1.304955
57	C	3.349155	-2.957266	-3.287196
58	C	4.555955	-3.290078	-2.670235
59	H	3.836585	-2.297012	0.495781
60	H	1.376000	-2.129067	-3.027624
61	H	5.650288	-3.302705	-0.809844
62	H	3.207854	-3.147465	-4.347871
63	H	5.361278	-3.738934	-3.245088
64	H	1.240123	1.068623	2.024848
65	H	-0.392568	-2.864831	2.376082
66	O	1.780321	1.005996	-1.624341
67	H	0.492436	-0.343441	-1.681885

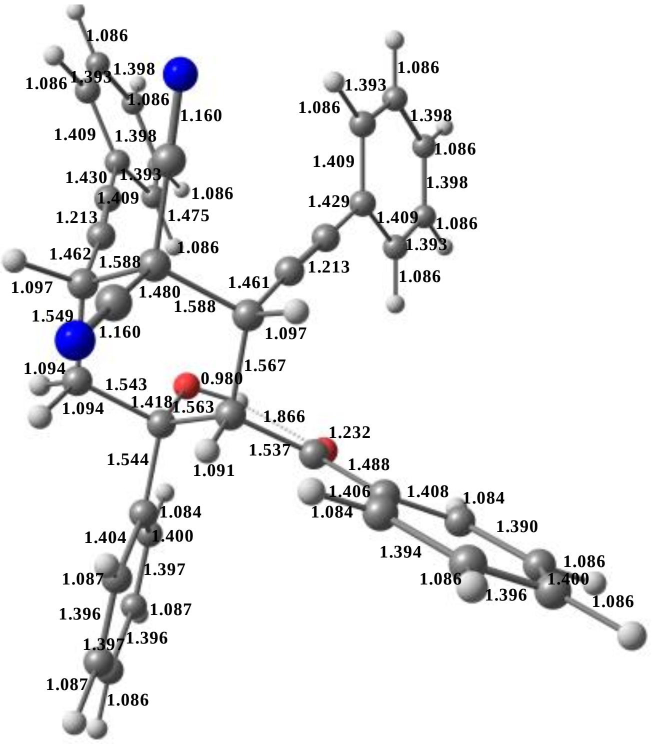

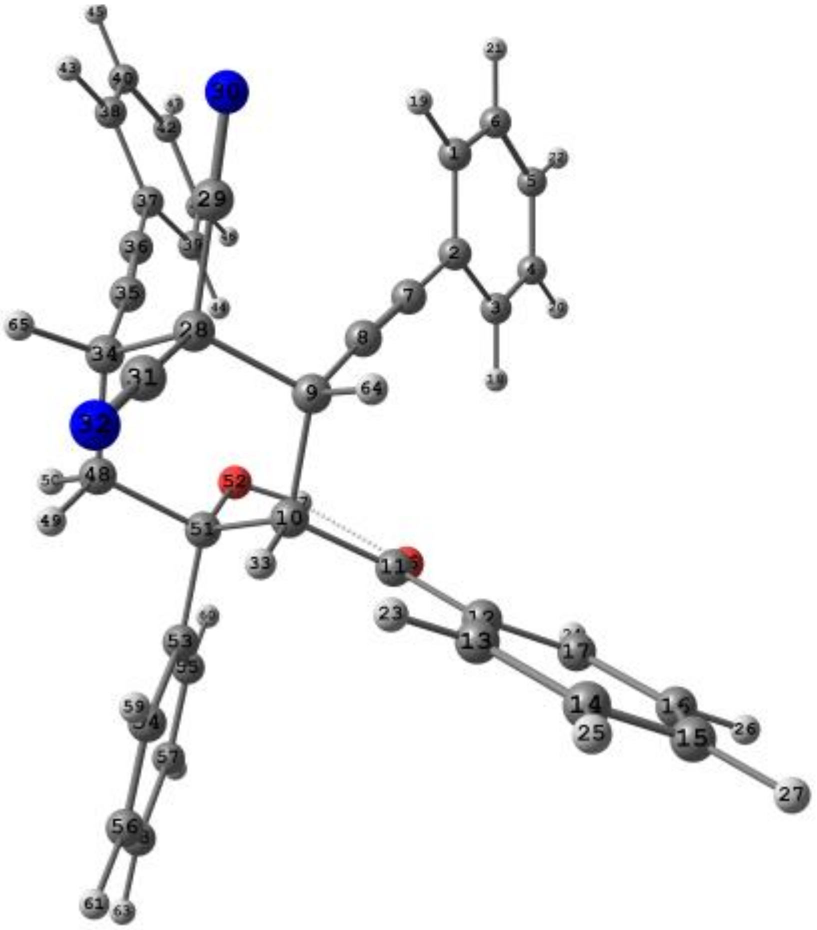

2a15

Energy **E(B3LYP) =-1685.3916302 h, G^{298} =-1684.938962 h, μ= 6.93 D**

Cartesian coordinates, Å

N	atom	x	y	z
1	C	-0.897640	5.368034	-0.204871
2	C	-1.710444	4.299331	0.220813
3	C	-2.838105	4.561750	1.022474
4	C	-3.143322	5.870894	1.387649
5	C	-2.333042	6.927636	0.962349
6	C	-1.211895	6.673046	0.167120
7	C	-1.389358	2.960187	-0.163788
8	C	-1.104237	1.828229	-0.499907
9	C	-0.785111	0.466882	-0.939100
10	C	-1.441128	-0.608308	0.010638
11	C	-1.641444	-1.933193	-0.749319
12	C	-2.815350	-2.072414	-1.666578
13	C	-3.804129	-1.085992	-1.831074
14	C	-4.865925	-1.291940	-2.711983
15	C	-4.956268	-2.482150	-3.436179
16	C	-3.978331	-3.470735	-3.280649
17	C	-2.917885	-3.266348	-2.405171
18	H	-3.464208	3.738204	1.351256
19	H	-0.028536	5.163947	-0.822341
20	H	-4.014649	6.066582	2.005751
21	H	-0.580954	7.492562	-0.164342
22	H	-2.574496	7.946797	1.250130
23	H	-3.765308	-0.152436	-1.280214
24	H	-2.151302	-4.022608	-2.276290
25	H	-5.622982	-0.522464	-2.829349
26	H	-4.045439	-4.397253	-3.843300
27	H	-5.785586	-2.640121	-4.119755
28	C	0.791627	0.300815	-1.073938
29	C	1.345787	1.477305	-1.778150
30	N	1.792339	2.395941	-2.327657
31	C	1.138205	-0.868809	-1.909858
32	N	1.433689	-1.756843	-2.594425
33	H	-2.430184	-0.214287	0.254860
34	C	1.460112	0.184229	0.355228
35	C	2.909360	0.032654	0.250881
36	C	4.110152	-0.113476	0.158743
37	C	5.526808	-0.276012	0.047756
38	C	6.147024	-1.458515	0.496724
39	C	6.318382	0.744068	-0.515897
40	C	7.527156	-1.612120	0.383444
41	C	7.697492	0.580385	-0.626797
42	C	8.305336	-0.595702	-0.178080
43	H	5.539924	-2.247050	0.930592
44	H	5.842944	1.655986	-0.863695
45	H	7.996140	-2.527432	0.733064
46	H	8.298898	1.372354	-1.063902
47	H	9.380944	-0.719727	-0.265730

48	C	0.818300	-0.963917	1.162434
49	H	1.003343	-1.910324	0.650981
50	H	1.309135	-1.000017	2.139020
51	C	-0.692378	-0.751692	1.379749
52	O	-0.878554	0.457117	2.139663
53	C	-1.336398	-1.853800	2.234755
54	C	-0.723758	-3.086728	2.491164
55	C	-2.599713	-1.607623	2.795146
56	C	-1.357180	-4.048446	3.282555
57	C	-3.237250	-2.569444	3.578832
58	C	-2.616905	-3.796323	3.826756
59	H	0.250921	-3.315292	2.076092
60	H	-3.076076	-0.646160	2.632883
61	H	-0.860630	-4.996493	3.470844
62	H	-4.215454	-2.356244	4.001510
63	H	-3.109004	-4.545733	4.440652
64	H	-1.200122	0.331286	-1.944929
65	H	1.234138	1.132578	0.858218
66	O	-0.840683	-2.851007	-0.623444
67	H	-0.951029	1.220227	1.539601

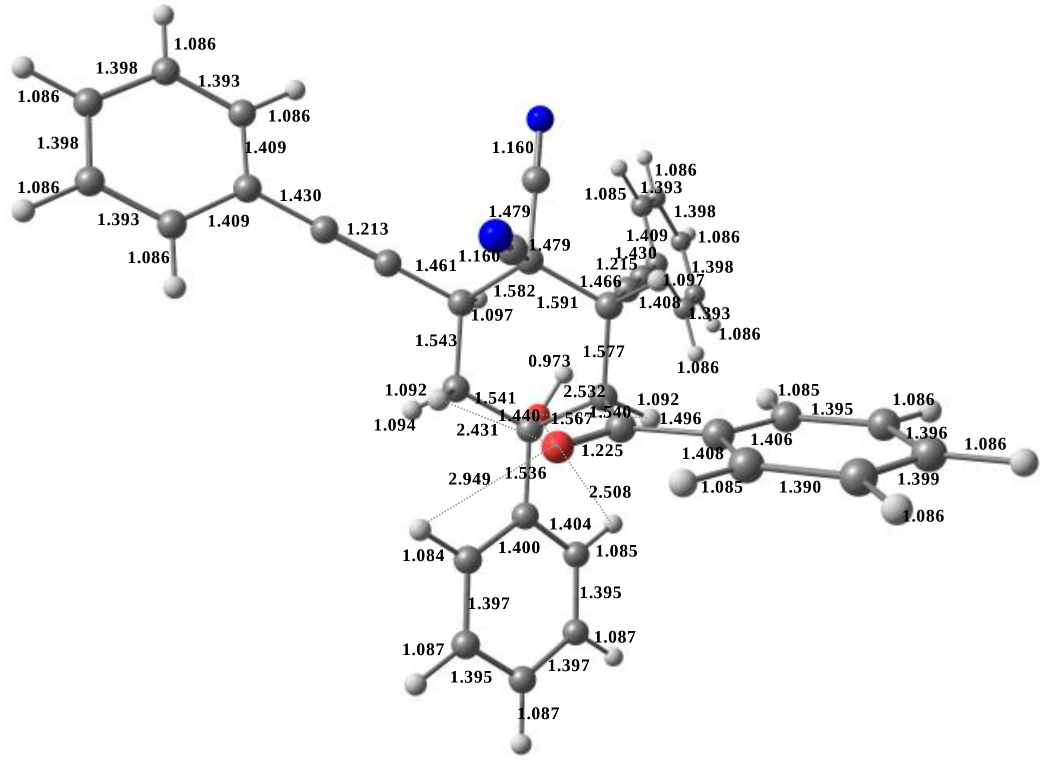

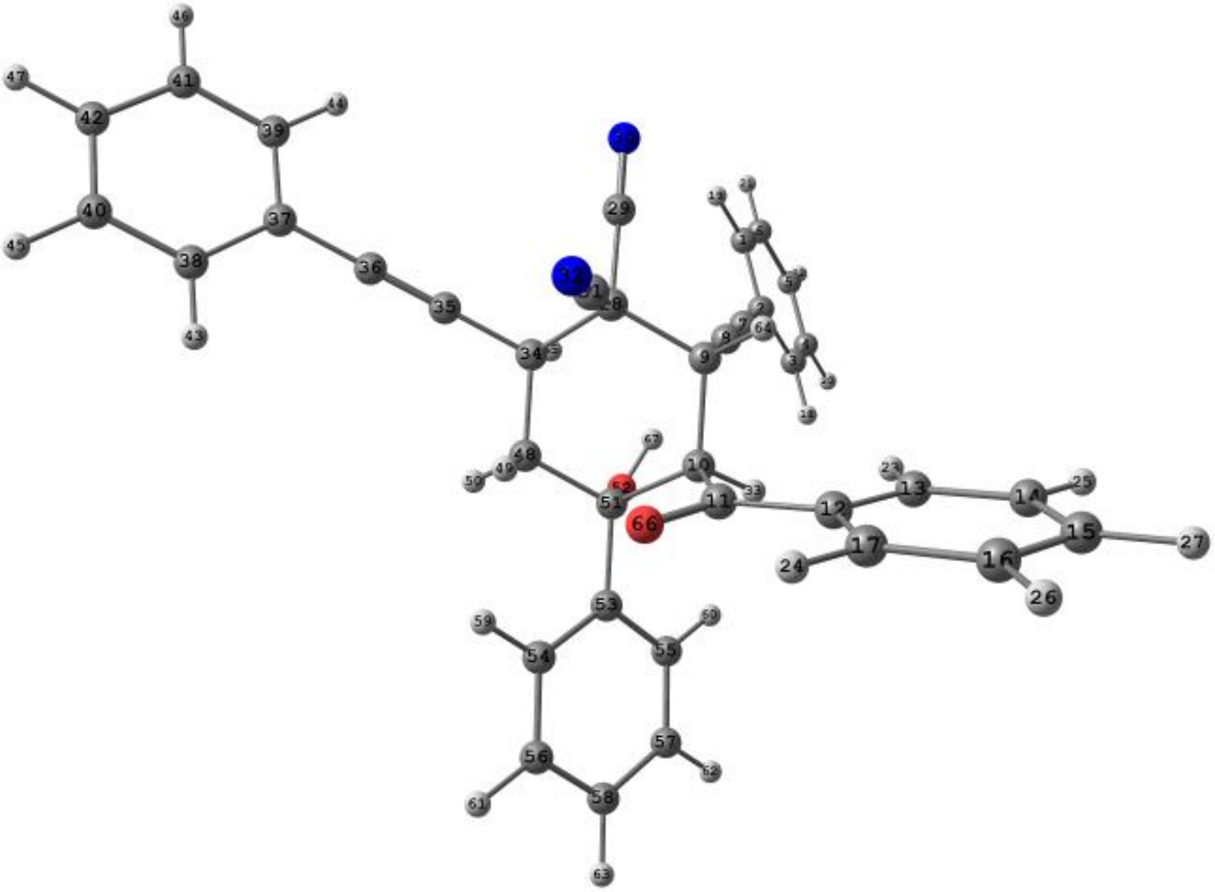

2**a16**

Energy **E(B3LYP) = -1685.39347046 h, G^{298} =-1684.942186 h, μ= 8.84 D**

Cartesian coordinates, Å

N	atom	x	y	z
1	C	-2.930985	-4.647204	-0.202539
2	C	-3.094206	-3.272648	-0.464719
3	C	-4.341113	-2.801388	-0.920082
4	C	-5.397813	-3.689846	-1.108010
5	C	-5.228888	-5.052265	-0.844841
6	C	-3.994528	-5.526893	-0.391967
7	C	-2.003426	-2.368359	-0.270235
8	C	-1.070586	-1.609426	-0.109732
9	C	0.049963	-0.694374	0.098117
10	C	-0.353625	0.801467	-0.125436
11	C	-1.604130	1.228957	0.655364
12	C	-2.887068	1.502217	-0.058357
13	C	-3.109848	1.200139	-1.413215
14	C	-4.343730	1.475729	-2.003753
15	C	-5.364971	2.063515	-1.254176
16	C	-5.153922	2.369943	0.094666
17	C	-3.928394	2.087082	0.687198
18	H	-4.469015	-1.742840	-1.124398
19	H	-1.970560	-5.012035	0.148300
20	H	-6.355662	-3.317727	-1.460265
21	H	-3.859430	-6.584921	-0.186392
22	H	-6.055686	-5.741261	-0.992046
23	H	-2.338679	0.734617	-2.015943
24	H	-3.751724	2.316603	1.732378
25	H	-4.504405	1.233683	-3.050044
26	H	-5.945996	2.828011	0.679817
27	H	-6.322220	2.282757	-1.718551
28	C	0.787772	-0.997755	1.467469
29	C	1.320373	-2.376947	1.390088
30	N	1.726661	-3.461684	1.328246
31	C	-0.114703	-1.005171	2.640692
32	N	-0.756822	-1.090206	3.602831
33	H	-0.549636	0.876686	-1.195404
34	C	1.980351	0.025565	1.716466
35	C	3.170950	-0.331623	0.942042
36	C	4.198458	-0.645618	0.378470
37	C	5.400272	-1.019682	-0.299828
38	C	6.295828	-0.037064	-0.765732
39	C	5.704123	-2.379682	-0.508095
40	C	7.466804	-0.410554	-1.422053
41	C	6.876043	-2.742906	-1.168306
42	C	7.760477	-1.761883	-1.625955
43	H	6.065354	1.012109	-0.607736
44	H	5.015523	-3.138969	-0.150218
45	H	8.150996	0.355243	-1.776384
46	H	7.100015	-3.794334	-1.324702
47	H	8.673842	-2.049060	-2.139062

48	C	1.499272	1.480554	1.504861
49	H	0.773644	1.707276	2.287442
50	H	2.357381	2.145917	1.648460
51	C	0.865715	1.746273	0.125341
52	O	1.766961	1.354983	-0.919609
53	C	0.463539	3.219477	-0.066139
54	C	0.134287	4.068281	1.000942
55	C	0.410081	3.736254	-1.370473
56	C	-0.235107	5.395560	0.769323
57	C	0.037265	5.060362	-1.602274
58	C	-0.286475	5.896995	-0.531833
59	H	0.156983	3.706010	2.022471
60	H	0.679113	3.096083	-2.203918
61	H	-0.481484	6.036068	1.611768
62	H	0.004718	5.438695	-2.620478
63	H	-0.573692	6.929568	-0.710172
64	H	0.820626	-0.889732	-0.656185
65	H	2.241808	-0.072298	2.777260
66	O	-1.533545	1.366557	1.873111
67	H	2.613653	1.812258	-0.781274

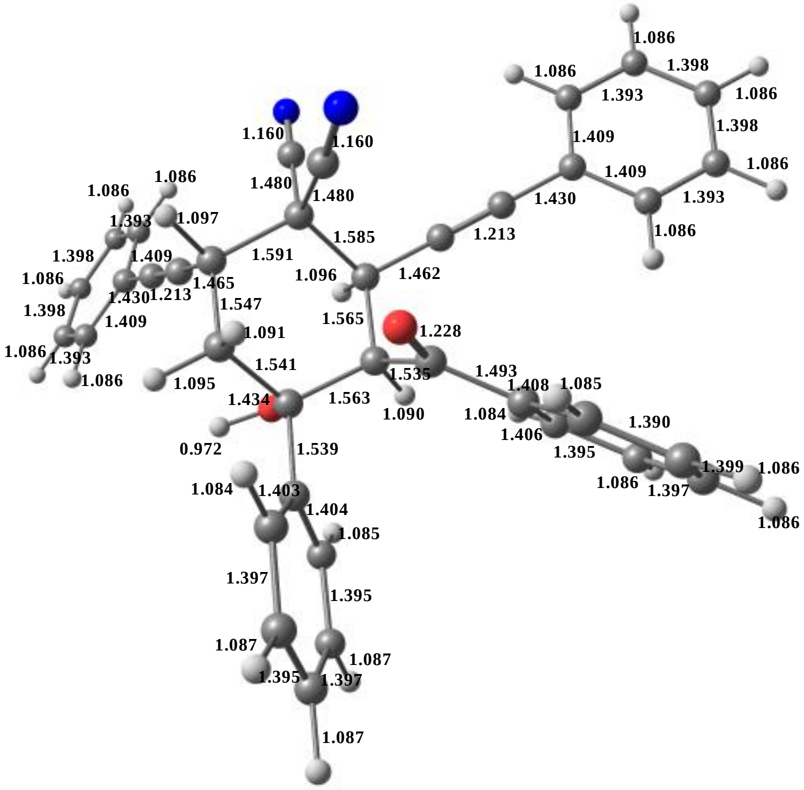

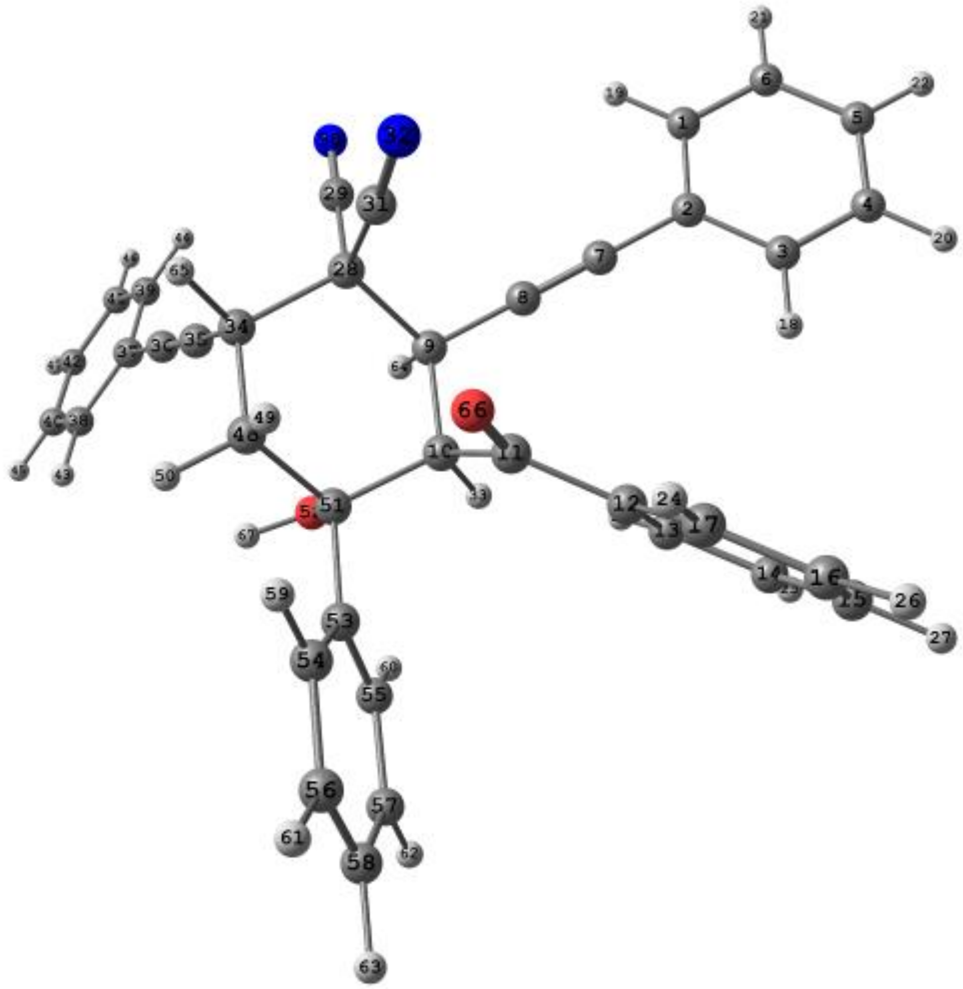

Supplement: Supplementary file 1 [file molecules-25-05920-s001.pdf]
